# Supplementary material for: Projected population level impact and cost-effectiveness of clinic and community-based tuberculosis screening approaches
Source: PLOS Glob Public Health. 2026 Sep 11;6(9):e0006738. doi: 10.1371/journal.pgph.0006738 (PMC13567835; doi:10.1371/journal.pgph.0006738)
Supplement: Supplementary file 1 [file pgph.0006738.s001.pdf]

Projected population level impact and cost-effectiveness of clinic and community-based tuberculosis screening approaches

S1 Appendix. Additional methods, results, and discussion

Contents

1 Supplementary Methods 4

1.1 Description of the mathematical model 4

1.1.1 Key 4

1.1.2 Use of expert opinion to inform model parameter ranges 4

1.1.3 People 5

1.1.4 Model initialisation 5

1.1.5 Model scheduling 5

1.1.6 Demography 6

1.1.7 TB symptoms 6

1.1.8 TB infectiousness 10

1.1.9 Clinic visiting and diagnosis 11

1.1.10 HIV/ART 16

1.1.11 Tuberculosis 17

1.1.12 Infection prevalence in child household contacts 28

1.1.13 Interventions 29

1.1.14 Calibration 32

1.1.15 Input parameters 35

1.1.16 Calibration targets table 44

1.2 Costing methods 50

1.3 CEA Outcomes 54

2 Supplementary Results 55

2.1 Model calibration 55

2.1.1 Fit to data 55

2.1.2 TB mortality rates in people with chronic conditions 55

2.2 Additional results figures 55

2.3 Partial rank correlation coefficients 58

2.4 Additional cost-effectiveness results 59
3 Additional discussion 68
4 CHEERS 2022 Checklist 70
5 References 73

1 Supplementary Methods

1.1 Description of the mathematical model

1.1.1 Key

Model parameter names are written in italics, with colour indicating whether the parameter is an input parameter, a parameter with a global model-wide value, calculated from input parameter(s) or other values, or an individual-level parameter, which can take a different value for each simulated person.

In variable names, the terms HIV0, HIV1, and HIV2, are used to refer to people who are HIV-, HIV+ART-, and HIV+ART+ respectively.

m/f is used in parameter names to indicate that the parameter value varies by sex. *hiv0/1/2* and *hiv01/2* are used in parameter names to indicate that the parameter value varies by HIV and ART status and ART status only respectively.

1.1.2 Use of expert opinion to inform model parameter ranges

It is a common misconception that adding additional complexity to mathematical models inevitably involves making additional assumptions, particularly if the data available to inform parameterisation are limited or of poor quality. This is usually not the case – instead, adding complexity makes implicit assumptions explicit, and allows the impact of the assumptions on the results to be explored. To give a simple example, if we choose to explicitly include sex in our model, we will make a range of explicit assumptions around how sex influences factors such as rates of developing disease, treatment rates, etc. If we do not explicitly include sex we still make assumptions, however we will be obliged to make the implicit assumptions that sex does not affect rates of developing disease, treatment rates, etc, and will not be able to explore the sensitivity of the results to the assumptions we made.

With that in mind, there are a numbers of areas in the model structure (particularly related to the impact on TB natural history of conditions other than TB that can cause TB symptoms) where we considered that it was important to include complexity, despite a lack of data to inform model parameterisation. For these areas, we relied on expert opinion to determine plausible ranges for the model parameters, favouring broad and inclusive ranges over more precise estimates. Ranges were obtained by consensus between three of the publication's authors, Alison Grant, Palwasha Khan, and Indira Govender, all of who are clinicians and TB epidemiologists with extensive experience of primary healthcare in South Africa, along with advice from other South Africa experts.

1.1.3 People

The main state variables assigned to people in the model were:

- Unique ID – *person_ID*
- Sex – *sex* (male, female)
- TB status – *TB_status* (uninfected, latent, subclinical disease, clinical disease, on treatment)
- Individual-level TB infectiousness – *infectiousness* (numeric, see Section 1.1.12 'Individual-level variation in infectiousness)
- HIV/ART status – *HIV_status* (HIV-, HIV+ART-, HIV+ART+)
- Whether they have a chronic health condition (other than or in addition to TB) that causes TB symptoms – *chronic_status*
- Their baseline clinic visiting rate – *my_baseline_clinic_rate* – and an adjusted rate that accounts for any higher clinic visiting rate in people with symptoms, and for higher clinic visiting rates for people on ART – *my_adjusted_clinic_rate*

Other state variables were used to track individuals' histories in the model, for the purpose of creating model output.

1.1.4 Model initialisation

To initialise the model, 20000 people were created. The newly created people were each assigned a *sex*, with a probability of *prop_male* of being male and 1 - *prop_male* of being female. They were then assigned a time to (non-HIV and non-TB) death, set equal to:

> minimum (*max_age* – *min_age*, (*min_background_mort_age_m/f* – *min_age* – ln (1 - random-float 1) / *background_mort_m/f*)

A random *infection_seed_proportion* were seeded with latent infection, with no risk of progression without reinfection. A random *tb_seed_proportion* were seeded with subclinical TB disease.

The model was run from 1850 to allow the population age distribution and TB incidence and mortality to reach equilibrium, before the introduction of HIV in *hiv_intro_year*.

1.1.5 Model scheduling

The majority of events in the model were simulated using continuous time.

The exceptions to this were the *Mtb* transmission process, which used a monthly time step, and the production of model output, which was done annually.

1.1.6 Demography

Individuals were introduced into the model at age 15 (*min_age*). People aged <15 were not modelled, as the risk of *Mtb* transmission from children is low[1], and the simulated intervention approaches considered screening in adults only.

A constant population size was simulated, with a new person created whenever someone died in the model.

There were four types of mortality in the model

- HIV mortality
- TB mortality
- Background mortality
- All individuals die upon reaching the age of 80 years (*max_age*)

The background mortality started at age *min_background_mort_age_m/f*, and had a rate of *background_mort_m/f* per year. The values of *min_background_mort_age_m/f* and *background_mort_m/f* were fixed, and chosen to give an approximate fit to the age distribution by sex in South Africa in 2021[2].

TB and HIV mortality are described in the sections on TB and HIV.

1.1.7 TB symptoms

We define TB symptoms in line with the 2018 South Africa TB prevalence survey: reporting one or more of cough (persistent of any duration), drenching night sweats, unexplained weight loss, and unexplained fever for at least two weeks[3]. Approximately 15% HIV-negative people and 23% of HIV-positive people without TB were symptom-screen positive in the 2018 South African TB prevalence survey[4], demonstrating that TB symptoms are relatively common even in people without TB.

Mathematical models of tuberculosis that explicitly model TB symptoms have typically divided the bacteriologically-positive TB state into two compartments, symptom-screen negative and symptom-screen positive (often referring to the compartments as subclinical or asymptomatic and clinical or symptomatic respectively). With this model structure, people enter the symptom-screen negative compartment when first developing bacteriologically-positive TB. This results in a model structure where the probability of reporting symptoms falls from frequently >10% in people without bacteriologically positive TB to zero in people after first they first develop bacteriologically positive TB. We therefore explicitly take into account in our model structure the presence of people with bacteriologically-positive TB and TB symptoms whose symptoms are not caused by TB.

Recent WHO guidance[5] defines asymptomatic TB as "*A person with TB disease who did not report symptoms suggestive of TB during screening*" – i.e. a pragmatic division into asymptomatic and symptomatic TB based on whether someone reports TB symptoms, regardless of the underlying cause of the symptoms. The slightly earlier International Consensus for Early TB framework (ICE-TB)[6] took a more conceptual approach to classifying early TB states, saying that "*we agreed that each conceptual state should reflect pathophysiological processes, rather than be bound solely by practical considerations, such as the ability to identify states with existing diagnostic tools*", and describing symptoms as "*caused by the host response to M. tuberculosis".* ICE-TB also favoured the term subclinical over asymptomatic to refer to people with TB who do not recognise and report symptoms. For the purposes of this manuscript we retain this distinction, using the terms **asymptomatic** and **symptomatic TB** to refer to people with TB who do not or do report symptoms during screening (in line with WHO guidance), and the terms **subclinical** and **clinical TB** to refer to people without and with reported symptoms that are *caused by TB* respectively. People with subclinical TB can therefore be symptomatic or asymptomatic, based on the presence of absence of TB symptoms caused by conditions other than TB.

There are a wide range of conditions that can cause TB symptoms that are not TB, ranging from minor transient conditions such as the common cold, to chronic conditions such as chronic obstructive pulmonary disease. Transient conditions are likely to have little effect on TB model dynamics, beyond temporarily making some people symptom-screen positive. Chronic conditions may have a greater effect on model dynamics – for instance potentially increasing TB mortality rates in people with some chronic conditions, but also potentially making them detectable through passive case finding from as soon as they develop bacteriologically-positive TB.

In the model, we simplify conditions that cause TB symptoms that are not TB into two categories: chronic and transient. We assume that the presence or absence of chronic conditions is a permanent characteristic of simulated individuals that can increase the rate of developing disease and alter disease progression. We assume that people with chronic conditions always report symptoms when asked. Transient conditions are not modelled as a characteristic of people. Instead, all people in the model have a small, non-zero probability of reporting TB symptoms due to transient conditions each time they are symptom screened.

1.1.7.1 Development and loss of TB symptoms caused by TB

Few data are available to inform the rate at which people with subclinical TB develop clinical TB, or the rate at which people with clinical TB regress to subclinical TB. A recent review estimated that, across national prevalence surveys and adjusted for under-diagnosis, 17.2% of people with prevalent

bacteriological TB reported a persistent cough (duration ≥2 weeks), and 37.5% reported a cough of any duration (with figures of 10.3% and 16.8% respectively for South Africa)[7]. From this, we can estimate that an average of 54% of people with bacteriological TB who reported a cough reported that they had had their cough for less than 2 weeks (39% for South Africa). In a community prevalence survey conducted in KwaZulu-Natal, South Africa, there was a mean reported symptom duration in people with bacteriologically positive TB of 2.8 weeks, and a median duration of 2 weeks[8].

These estimates suggest that there may be a high rate of movement between the symptomatic and asymptomatic states. However reported symptom duration may be affected by inaccurate recall, and the national estimates are based on cough only (as opposed to the four-symptom screen). The data are also for reported symptoms regardless of cause, not symptoms caused by TB, and rates of loss of symptoms may by higher for symptoms caused by other things. For these reasons, we used a wide range for the rates of movement from clinical to subclinical TB in HIV negative people (i.e. loss of TB symptoms caused by TB), ranging from no regression to regression at a mean rate of 1 per month. We assumed that the rate of regression must be lower in people living with HIV than in HIV-negative people, and lower in PLWHIV who are not on ART than in PLWHIV who are on ART.

Calibrating the model to data from the South Africa National prevalence survey and to WHO data and estimates, most notably estimates of the proportion of people with prevalent TB who are symptom-screen negative, restricts the values of the rates of movement from subclinical to clinical TB relative to the rates of movement from clinical to subclinical TB. For this reason, we used wide uninformative ranges around the parameter controlling the rate of movement from subclinical to clinical TB. We assumed that the rate of progression must be higher in people living with HIV than in HIV negative people, and higher in PLWHIV who are not on ART than in PLWHIV who are on ART.

We assumed that having a chronic condition that causes TB symptoms would decrease the rate of regression from clinical to subclinical TB, and increase the rate of progression from subclinical to clinical TB (see Section 1.1.7.2 for details).

1.1.7.2 Chronic conditions that cause TB symptoms

We assumed that chronic conditions that cause TB symptoms can affect TB natural history and care seeking in a number of ways:

- Alter the rate of developing TB following infection
- Alter the rate at which people move between subclinical TB and clinical TB
- Alter the rate of self-cure for people with subclinical TB

- Alter the TB mortality rate for people with clinical TB
- Lead to passive treatment seeking and being tested for TB in people with subclinical TB
- Alter the level of infectiousness per unit time

Few empirical data are available to inform these rates, due to the complex array of conditions that can cause TB symptoms, and the difficulties in distinguishing whether symptoms are caused by TB or other conditions in people with TB. We therefore relied on expert opinion (see Section 1.1.2) to obtain broad plausible ranges for parameters related to the above factors, as well as for the proportion of prevalent TB symptoms in people without bacteriologically positive TB that are due to chronic conditions.

Proportion of prevalent TB symptoms in people without bacteriologically positive TB in South Africa that are due to chronic conditions

It was considered plausible that 30% - 70% of prevalent TB symptoms in HIV- people without bacteriologically positive TB are due to chronic conditions (*proportion_symp_chronic_hiv0*), with the remaining 70% - 30% due to transient conditions. It was considered that the proportion due to chronic conditions in HIV+ people was likely to be higher by an absolute value of 10% (*abs_increased_prop_symp_chronic_hiv*):

proportion_symp_chronic_hiv12 = *proportion_symp_chronic_hiv0* + *abs_increased_prop_symp_chronic_hiv*

Rate of developing TB following infection, and TB progression

It was considered plausible that having a chronic condition that causes TB symptoms could increase the rate of developing TB following infection (*increased_TB_development_chronic*), and the rate of progression from subclinical to clinical TB, by a ratio of 1.3 to 1.9, relative to people with no chronic conditions (*relative_rate_sub_to_clin_chronic*).

It was considered plausible that having a chronic condition that causes TB symptoms could decrease the rate of self-cure from subclinical TB (*relative_self_cure_rate_chronic*), and the rate of regression from clinical to subclinical TB (*relative_rate_clin_to_sub_chronic*), by a ratio of 1/1.3 to 1/1.9, relative to people with no chronic conditions.

Overall, it was considered plausible that having a chronic condition that causes TB symptoms could increase the overall rate of TB mortality by a ratio of 1.7 to 3, relative to people with no chronic conditions. As we modelled TB mortality occurring from clinical TB only, and the proportion of prevalent bacteriological TB that was clinical was higher for people with chronic conditions in the

model, we modelled this through assuming that the rate of TB mortality in the clinical state was 1.3 – 1.9 times higher for people with chronic conditions than for people without (*relative_TB_mortality_rate_chronic*). After the model was calibrated, we checked in the fitting parameter sets that this gave an overall rate of TB mortality for people with chronic conditions compared to those without of approximately 1.7 to 3 (see Section 2.1.2).

Passive diagnosis

It was considered plausible that the rate of seeking care for TB symptoms caused by chronic conditions only could be between 0.5 and 2 times that of the rate of seeking care for TB symptoms caused by TB (*clinic_TBsymp_adjust_chronic*).

Alter the level of infectiousness per unit time

We assume that the probability of transmission per contact for people with subclinical TB and a chronic condition is dependent of whether they have a cough. If they do not, we assume that the probability of transmission per contact is the same as for people with subclinical TB and no chronic condition. If they do have a cough, we consider it plausible that the probability of transmission per contact could be between 10% and 50% higher than for people with subclinical TB and no chronic condition. As the type of TB symptom (cough vs not) was not explicitly simulated, this was simulated as a single transmission multiplier (relative to clinical TB) applied to all people with subclinical TB and a chronic condition:

*transmission_multiplier_chronic * 0.587 + reduced_transmission_sub * (1 – 0.587)*

Where 0.587 is the proportion of all people who reported TB symptoms in the South Africa TB prevalence survey who reported a cough[9].

1.1.8 TB infectiousness

The parameter in our model *reduced_transmission_sub* controls the relative infectiousness per unit time of people with subclinical TB and no chronic condition, relative to people with clinical TB, encompassing both differences in contact behaviour and in the probability of transmission per contact. From a physiological perspective, we consider it unlikely subclinical TB is more infectious per unit time than clinical TB. Having TB symptoms may reduce the amount of contact that someone has with other people however. We therefore set a range for *reduced_transmission_sub* of 0.2 to 1.3. Infectiousness in people with subclinical TB and chronic conditions is described in Section 1.1.7.2

1.1.9 Clinic visiting and diagnosis

1.1.9.1 Overview

We model two separate types of clinic visit: visits made seeking care for TB symptoms, and visits made for any other reason (not seeking care for TB symptoms). These types of clinic visit vary in the rates at which they occur, and in the probability of TB screening and testing occurring during the clinic visit. Clinic visiting by people with TB is explicitly modelled throughout the whole model run. Clinic visiting by people without TB is modelled from 1990 only, as it greatly increases the model runtime, and has negligible impact on TB dynamics in the baseline model.

Empirical data from clinic visit surveys conducted in South Africa suggest that even in people reporting TB symptoms, seeking care for those symptoms is the reason for the clinic visit in only 9%[10] or 35%[11] of visits. Therefore, we model a rate of clinic visiting not seeking care for TB symptoms in all simulated individuals, including those who do have TB symptoms (see Section 1.1.9.2). On these visits, we assume that there is no probability of diagnosis for people not on ART (in the absence of clinic-based interventions), and that diagnosis can occur through an active case finding pathway for people on ART.

In addition, simulated individuals with TB symptoms caused by chronic conditions and/or TB have a rate of seeking care for those symptoms in the model, and we simulate a rate of seeking care for TB symptoms caused by transient conditions for all individuals without TB symptoms caused by TB or chronic conditions. On each visit seeking care for TB symptoms, these individuals have a probability of diagnosis through active case finding (if on ART or in the clinic-based intervention scenarios), and also have an additional probability of diagnosis through passive case finding.

1.1.9.2 Rate of clinic visits made for any other reason (not seeking care for TB symptoms)

1.1.9.2.1 Individual-level baseline clinic visiting rates

1.1.9.2.1.1 Data

Data used to determine how rates of clinic visiting vary by sex and ART status, and between individuals, were taken from the Africa Health Research Institute (AHRI) demographic surveillance area (DSA) in KwaZulu-Natal, South Africa, from 1st February 2017 to 29th February 2020. The data sources are described in Randera-Rees *et al* 2021[12]. The data used were limited to people who were aged 15 years and over. It was not possible to distinguish in the data between people who did not visit a clinic at all, and people who visited a clinic but did not consent to their data being recorded. To reduce underestimation of clinic visiting rates, we therefore limited the analysis to people who

had consented to have at least one HIV test conducted as part of a DSA serosurvey – people who we considered were also likely to consent to their clinic visits being recorded.

ART interruptions were not explicitly simulated in the model, and periods of treatment interruption contributed only 8.3% of all person time for people who were or had previously been on ART in the dataset. In the analysis, we therefore considered individuals to be on ART from the first date at which a clinic visit for ART-related reasons was recorded, including during interruptions.

HIV status was unknown for 53% of person-time for people not on ART, and there was little difference in the mean rate of clinic visiting between people who were HIV positive and not on ART and of unknown HIV status (0.70 and 0.76 visits per year respectively). Visits for people who were known to be HIV negative were higher (1.4 per year), however it is likely that this is an overestimate – only people who had a recent negative HIV test at a clinic or in a DSA sero-survey were considered to be HIV negative. Limiting the analysis to people who were recorded as visiting a clinic at least once during the data collection period greatly reduced the difference between HIV negative people and people with unknown HIV status (to 2.7 and 2.2 visits per year respectively). Patterns of clinic visiting were therefore assumed to be the same in all people who were not considered to be on ART.

For each sex and ART category, a gamma distribution was fitted to the data on the annual rate of clinic visiting per person (**Error! Reference source not found.**).

Figure 1 Distribution of the annual rate of clinic visiting in the data and fitted gamma distribution, by sex and ART. The x-axis shows the cumulative proportion of who visit clinics at at least the given rate.

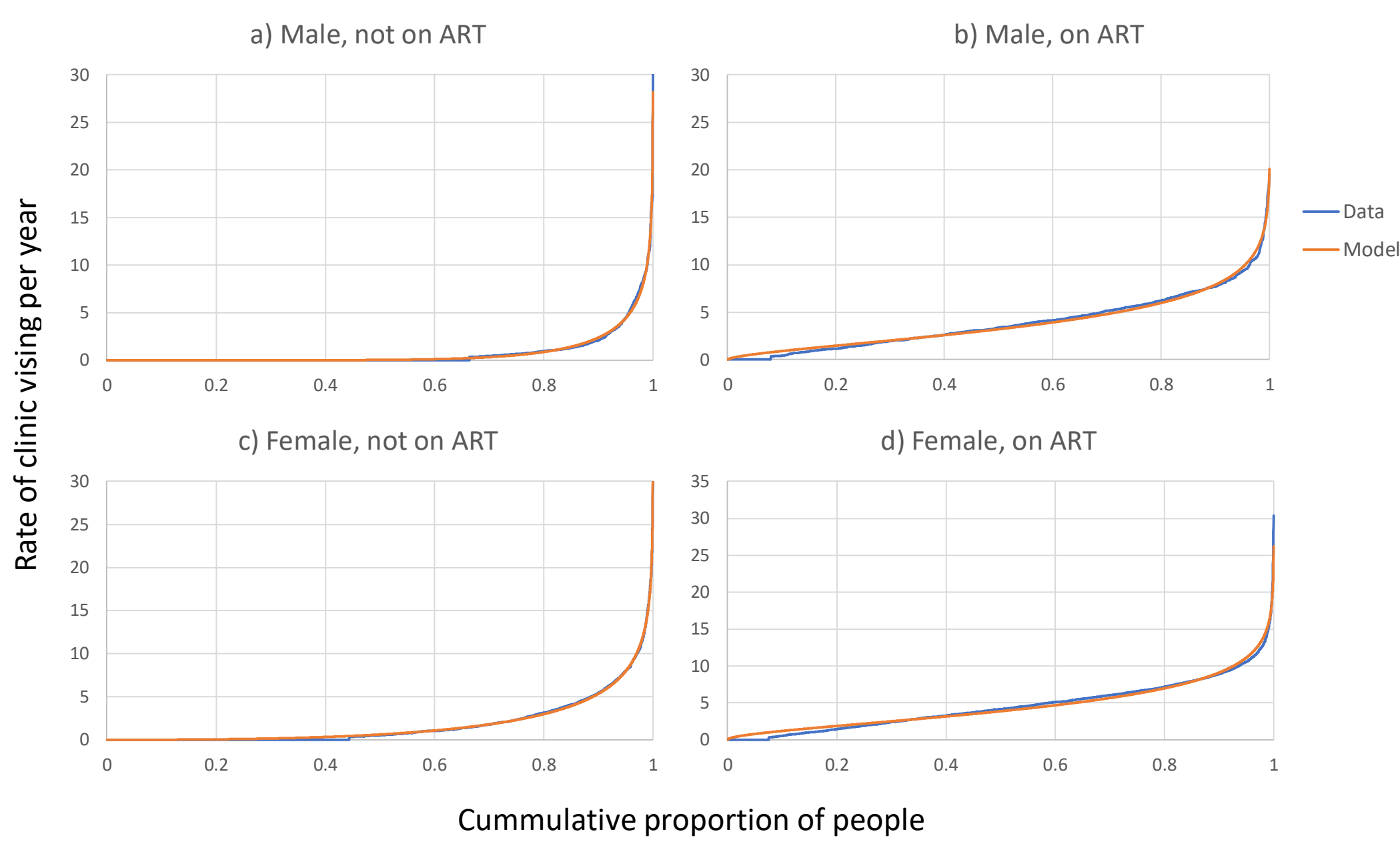

1.1.9.2.1.2 Model

In the model, an initial baseline rate at which a person visited clinics for reasons other than seeking care for TB symptoms was sampled at their creation at random from a gamma distribution, with the parameter values varying by sex (**Error! Reference source not found.**). The baseline rate is then multiplied by *clinic_visit_adjust*, a parameter that allows the models to be calibrated to National data on clinic visit rates (see Section 1.1.9.5). This gives an individual their final baseline rate of visiting clinics, *my_baseline_clinic_rate*. Upon starting ART, their *my_baseline_clinic_rate* and time of next clinic visit were resampled.

1.1.9.2.2 Adjustments to clinic visit rates in people with TB symptoms and/or TB

Empirical data suggest that people who report TB symptoms may have higher rates of visiting clinics, and that this may not be entirely explained by them seeking care for those symptoms. For instance, in one clinic exit survey in South Africa, 50% of respondents reported TB symptoms, with those symptoms the main reason for attending the clinic for only 35% of them[11]. It is plausible that the relative increase in visiting rates may be lower for people on ART compared to people who are HIV- or HIV+ART-, due to their high rates of clinic visiting for routine HIV care. We assume that having TB symptoms caused by chronic conditions and/or TB increases the rate of clinic visiting by a factor of:

- *clinic_other_symp_hiv01* in people on ART
- *clinic_other_symp_hiv2* = 1 + (*clinic_other_symp_hiv01* – 1) * *clinic_other _hiv2_vs_hiv01* in people who are not on ART

We further assume that having bacteriologically positive TB (regardless of symptoms) may be associated with an increased rate of clinic visiting, due to the existence of comorbidities (e.g. diabetes) that both increase clinic visiting rates, and increase the risk of developing TB. This is modelled by increasing the clinic visiting rate in people with TB by a factor of:

- *clinic_other_tb_hiv01* in people on ART
- *clinic_other_tb_hiv2* = 1 + (*clinic_other_tb_hiv01* – 1) * *clinic_other _hiv2_vs_hiv01* in people who are not on ART

Transient conditions causing TB symptoms are simulated as a probability of reporting symptoms when asked in people without TB symptoms caused by TB or chronic conditions, and not as an individual-level characteristic. They are therefore simulated in clinic attendees as an increased probability of reporting symptoms in clinic attendees compared to the general population, rather than as an increased rate of attending clinics in people with TB symptoms. The probability of a clinic attendee reporting symptoms due to transient conditions is calculated as:

prob_transient_clinic_hiv0/1/2 = (prob_transient_comm_hiv0/1/2 * clinic_other_symp_hiv01/2 / (prob_transient_comm_hiv0/1/2 * clinic_other_symp_hiv01/2 + (1 - prob_transient_comm_hiv0/1/2)))

where:

- *prob_transient_comm_hiv0* = (*prop_with_symp_hiv0* - *prop_with_symp_hiv0* * *proportion_symp_chronic_hiv0*)/(1 - *prop_with_symp_hiv0* * *proportion_symp_chronic*)
- *prob_transient_comm_hiv1/2* = (*prop_with_symp_hiv1/2* - *prop_with_symp_hiv1/2* * *proportion_symp_chronic_hiv12*)/(1 - *prop_with_symp_hiv1/2* * *proportion_symp_chronic_hiv12*)

and:

- *prop_with_symp_hiv1* = *prop_with_symp_hiv0* + *prop_with_symp_input_hiv1* * (1 - *prop_with_symp_hiv0*)
- *prop_with_symp_hiv2* = *prop_with_symp_hiv0* + *prop_with_symp_input_hiv1* * *prop_with_symp_input_hiv2* * (1 - *prop_with_symp_hiv0*)

1.1.9.3 Rate of clinic visiting seeking care for TB symptoms

We model a rate of seeking care for TB symptoms caused by TB that varies by HIV and ART status, equal to *clinic_TBsymp_HIV0/1/2*. The rate of seeking care for TB symptoms could be higher or lower in people whose symptoms are caused by chronic conditions only, and the rates are therefore multiplied by *clinic_TBsymp_adjust_chronic* in those individuals.

For people without TB symptoms caused by TB or chronic conditions, we model a rate of seeking treatment for TB symptoms caused by transient conditions equal to:

*prob_transient_comm_hiv0/1/2 * clinic_TBsymp_HIV0/1/2 * clinic_TBsymp_adjust_chronic * clinic_TBsymp_adjust_transient*

allowing for a lower rate of seeking treatment for TB symptoms caused by transient conditions compared to TB symptoms caused by TB and/or chronic conditions.

1.1.9.4 Screening and diagnosis

Supplementary material 2 shows the simulated active screening pathway simulated in the model in the baseline scenario. In brief, we assume that people on ART have a probability of receiving an Xpert Ultra if it has been at least a year since they last received an Xpert Ultra through active case

finding, or at least 6 months if they report TB symptoms. Individuals reporting TB symptoms who are not able to produce sputum are referred to a doctor, with an approximately 24.6% chance that they will be diagnosed with TB. The same screening process is followed when people first start ART. In line with expert opinion (see Section 1.1.2), we assume that there is a low probability of active screening for people not on ART, and therefore do not simulate active screening in that group.

We assume that there is an additional probability of diagnosis and starting treatment on each clinic visit made by people seeking care for TB symptoms, equal to *passive_diagnosis_prob*.

1.1.9.5 Calibration targets

National level data from South Africa reported a mean of 1.7 primary healthcare visits per year in 2022 for all ages[13], and there are some data to indicate that the mean rate of clinic visiting may not vary greatly between 0-14 year olds and 15+ year olds[14]. We therefore calibrated the model so that the overall mean rate of clinic visiting per year in 2022 was 1.5 – 1.9.

We calibrated the model to a 0.66 – 4.63 times higher prevalence of TB in clinic attendees relative to in people in the general population in 2022, informed by data from clinic-based and community-based TB surveys conducted in KwaZulu-Natal, South Africa[8,15]. The upper bound was increased from the crude estimate of 2.87 to account for the much lower proportion of people able to provide a sputum sample in the clinic-based survey compared to in the community-based survey. The lower bound was decreased from the crude estimate of 1.04 to account for the fact that only people who were screen positive were eligible to provide a sputum sample in the community survey.

Data on the prevalence of TB symptoms in clinic attendees in South Africa are limited, and estimates are highly variable. In one clinic exit survey, 64% of people reported symptoms, however only 19% of eligible people participated, and it is plausible that participation was higher among people with symptoms[10]. In a second clinic exit survey, 50% of people reported symptoms, however the authors reported that "*Among those screened for eligibility to the study, the proportion of participants reporting TB symptoms (50%) seems very high and we could not verify if study staff at times pre-screened participants*"[11]. In a third survey, only 6.4% of people reported symptoms[15]. In line with expert opinion (see Section 1.1.2), we calibrated the model so that a maximum of 30% of simulated clinic attendees reported TB symptoms. We did not set a lower bound for the prevalence of TB symptoms in clinic attendees, as the model structure ensured that it could not be lower than the prevalence in the general population (by HIV/ART status).

1.1.10 HIV/ART

Three HIV states were simulated in the model: HIV-, HIV+ART-, and HIV+ART+.

People created in the model at age 15 years were all HIV-. From the introduction of HIV in the model in 1995, HIV- people became HIV+ART- at a rate that varied by sex and over time. HIV incidence had the value *hiv_inc_early_m/f_annual* from 1995 to 2000, decreased/increased linearly from *hiv_inc_early_m/f_annual* to *hiv_inc_mid_m/f_annual* between 2000 and 2020, and decreased/increased linearly from *hiv_inc_mid_m/f_annual* to *hiv_inc_mid_m/f_annual* * *hiv_inc_m/f_late_reduction* between 2023 and 2040.

ART was introduced in the model in 2010. From the introduction of ART, HIV+ART- people became HIV+ART+ at a rate that varied by sex. To capture changes in estimated ART coverage over time, the values of the ART start rates in the model were changed in 2017, from *ART_start_rate_early_m/f_annual* to *ART_start_rate_late_m/f_annual*. People starting ART were actively screened for TB, as described in Section 1.1.9.4.

From 2010, all HIV+ART- people starting TB treatment were made HIV+ART+.

HIV mortality was simulated as a constant rate of (non-TB) HIV-related mortality for all HIV+ART- people (*HIV1_mortality_rate*), and all HIV+ART+ people (*HIV2_mortality_rate*). This was estimated from UNAIDS estimates[16] of HIV-related mortality in adults in 2021 (excluding estimated numbers of deaths due to TB), divided by the estimated number of adults living with HIV, and assuming that mortality rates were 52% lower for HIV+ people on ART than HIV+ people not on ART[17].

1.1.10.1 HIV and TB

We assume that PLWHIV who are on ART have 1.47 – 10.8 times the rate of developing TB following infection compared to HIV negative people[18,19] (*increased_develop_tb_rate_HIV2_vs_HIV0*). We assume that PLWHIV who are not on ART can have 1.89 – 4.76 times the rate of developing TB following infection compared to PLWHIV who are on ART (*increased_develop_tb_rate_HIV1_vs_HIV2*), based on the findings of a systematic review, with widened bounds to reflect variation in CD4 counts[20].

There are few or no data available to inform how HIV and ART affect the rate of transitioning between subclinical and clinical TB, or mortality rates from untreated clinical TB. We therefore assumed that rates of developing TB symptoms caused by TB and TB mortality rates from untreated clinical TB must be higher for people who are HIV+ART- than for people who are HIV+ART+, and higher for people who are HIV+ART+ than for people who are HIV-. Similarly, we assumed that rates

of losing TB symptoms caused by TB must be lower for people who are HIV+ART- than for people who are HIV+ART+, and lower for people who are HIV+ART+ than for people who are HIV-.

1.1.10.2 Calibration targets

The model was calibrated to the estimated male and female adult HIV prevalence in 2000 and 2023, and to the estimated male and female adult ART coverage in 2016 and 2023[21]. The width of the confidence intervals around the estimated prevalence of HIV and ART coverage in South Africa were reduced greatly between the UNAIDS estimates published in 2023 and 2024. It is likely that the reduction in confidence interval widths for 2000 and 2016 reflect reduced uncertainty for more recent years following the 2022 South Africa HIV prevalence survey[22], and assumptions made in generating UNAIDS prevalence estimates, rather than a genuine reduction in uncertainty around the estimates for earlier years. For that reason, we calibrated the model to the wider 2023-generated uncertainty intervals for HIV prevalence in 2000 and ART coverage in 2016.

The model was also calibrated to the predicted male and female adult HIV prevalence in 2030 from the Thembisa model version 4.7[23], with widened uncertainty ranges. Finally, the model was calibrated to ratios of ART in 2023 vs 2019 by sex greater than one, which ensured that any otherwise fitting runs where ART coverage goes down in the future were excluded.

See Section 1.1.11.9 for details of the calibration targets for HIV-related TB.

1.1.11 Tuberculosis

1.1.11.1 Disease states

Each individual in the model was in one of five main TB states (uninfected, latent, subclinical TB (no TB symptoms caused by TB), clinical TB (TB symptoms caused by TB), on treatment), with the latent infection state subdivided by time since infection (Figure 2).

Figure 2 Simulated TB states. Red outlines indicate infectious states.

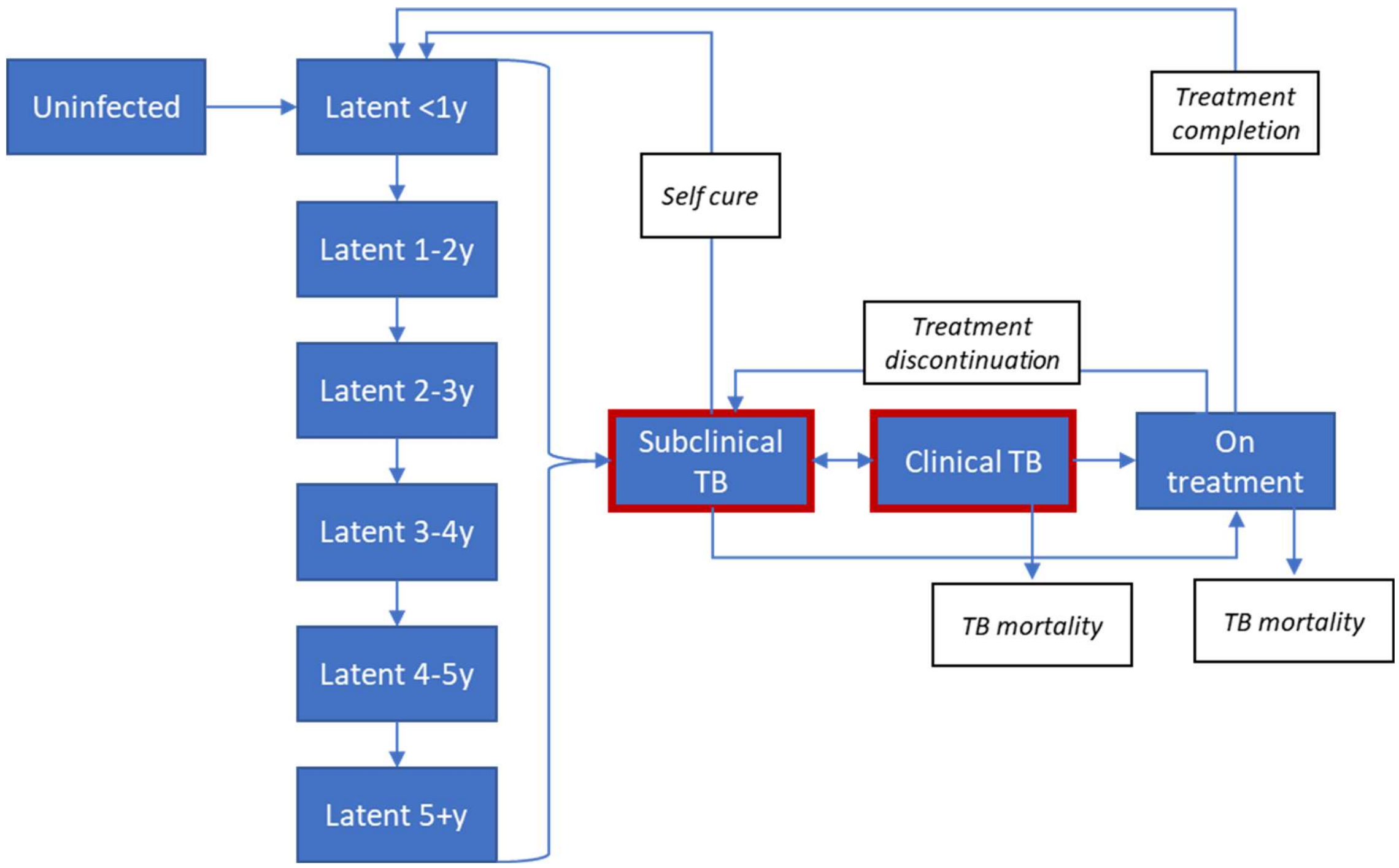

1.1.11.2 Disease progression

The rate of developing tuberculosis disease following infection depended on an individual's time since infection with *Mtb* and their HIV/ART status. The rate was highest in the first year, falling each year over the subsequent five years, and then lowest from five years following infection. The rates in each year were higher for HIV+ART+ people than for HIV- people by a factor of *increased_develop_tb_rate_HIV2_vs_HIV0*, and for HIV+ART- people compared to HIV+ART+ people by a factor of *increased_develop_tb_rate_HIV1_vs_HIV2*. The rates were also higher for people with chronic conditions, by a factor of *increased_TB_development_chronic*, and for men than women by a factor of *increased_TB_development_male*. As an example, rates of developing TB in the first year following infection were set equal to:

*develop_tb_y1_rate_HIV0 * rate_adjust_HIV * rate_adjust_sex * rate_adjust_chronic*

where

- *rate_adjust_HIV* =
  - 1 if HIV-
  - *increased_develop_tb_rate_HIV2_vs_HIV0* if HIV+ART+

    - *increased_develop_tb_rate_HIV2_vs_HIV0 * increased_develop_tb_rate_HIV1_vs_HIV2* if HIV+ART-
- *rate_adjust_sex* =
    - 1 / (*prop_male* + (1 - *prop_male*) / *increased_TB_development_male*) if male
    - 1 / (*prop_male* * *increased_TB_development_male* + 1 - *prop_male*) if female
- *rate_adjust_chronic* =
    - 1 / ((prop_with_symp_hiv0/1/2 * *proportion_symp_chronic_hiv0/12*) + (1 - (prop_with_symp_hiv0/1/2 * *proportion_symp_chronic_hiv0/12*)) / *increased_TB_development_chronic*) for people with a chronic condition
    - 1 / ((prop_with_symp_hiv0/1/2 * *proportion_symp_chronic_hiv0/12*) * *increased_TB_development_chronic* + 1 - (*prop_with_symp_hiv0/1/2* * *proportion_symp_chronic_hiv0/12*) for people without a chronic condition

Rates for subsequent years following infection were calculated in the same way.

The rate of developing disease also depended on the model year, being reduced by a factor of *decreased_tb_rates_late* in *change_TB_parameters_year* (see Section 1.1.11.8).

All simulated individuals who developed TB first developed subclinical TB (i.e. bacteriologically positive TB with no TB symptoms caused by TB, with or without TB symptoms caused by chronic conditions). From subclinical TB, people could progress to clinical TB (i.e. develop TB symptoms caused by TB), be diagnosed with TB and move to the treatment compartment (see Section 1.1.9), or self-cure.

Individuals with subclinical TB and no chronic conditions progress to clinical TB at a rate of:

- *rate_sub_to_clin_hiv1* if HIV+ART-
- *rate_sub_to_clin_hiv1* * *rate_sub_to_clin_hiv2_vs_hiv1* if HIV+ART+
- *rate_sub_to_clin_hiv1* * *rate_sub_to_clin_hiv2_vs_hiv1* * *rate_sub_to_clin _hiv0_vs_hiv2* if HIV-

These rates are increased by a factor of *relative_rate_sub_to_clin_chronic* for individuals with chronic conditions.

Individuals with subclinical TB and no chronic conditions self-cure at a rate of:

- *self_cure_rate_HIV0* if HIV-
- *self_cure_rate_HIV0* * *self_cure_rate_HIV2_vs_HIV0* if HIV+ART+

- *self_cure_rate_HIV0* * *self_cure_rate_HIV2_vs_HIV0* * *self_cure_rate_HIV1_vs_HIV2* if HIV+ART-

These rates are decreased by a factor of *relative_self_cure_rate_chronic* for individuals with chronic conditions.

Upon self-cure, individuals re-entered the first latent stage, resetting their time since infection back to zero. Individuals who progressed to disease again within 12 months of self-cure were not counted as having incident TB.

From clinical TB, people could regress to the subclinical TB stage, be diagnosed with TB and move to the treatment compartment (see Section 1.1.9), or die of TB.

Individuals with clinical TB and no chronic conditions regress to subclinical TB at a rate of:

- *rate_clin_to_sub_hiv0* if HIV-
- *rate_clin_to_sub_hiv0* * *rate_clin_to_sub_hiv2_vs_hiv0* if HIV+ART+
- *rate_clin_to_sub_hiv0* * *rate_clin_to_sub_hiv2_vs_hiv0* * *rate_clin_to_sub_hiv1_vs_hiv2* if HIV+ART-

These rates are decreased by a factor of *relative_rate_sub_to_clin_chronic* for individuals with chronic conditions.

Few data are available on the mortality rates of untreated clinical TB, particularly for PLWHIV. Using data from historical cohorts, Ragonnet *et al* estimated that the mortality rate of smear+ TB was 0.389 per year (95% credible interval 0.335 – 0.449)[24], with Richards *et al* arguing that the data used and estimated rate may better reflect mortality from clinical TB than smear+ TB[25]. These rates, estimated using data from populations before widespread availability of chemotherapy, are likely to be overestimates of the rates in populations where chemotherapy is available however. This is because the availability of treatment is likely to reduce the average severity of disease in the untreated population, with people with more severe disease having higher rates of starting treatment.

Calibrating the model to data and WHO estimates constrains the TB mortality rates in the calibrated parameter sets, and we therefore use wide, uninformative plausible ranges for the mortality rates.

Individuals with clinical TB and no chronic conditions die of TB at a rate of:

- *TB_mortality_rate_HIV1* if HIV+ART-
- *TB_mortality_rate_HIV1* * *TB_mortality_rate_HIV2_vs_HIV1* if HIV+ART+

- *TB_mortality_rate_HIV1* * *TB_mortality_rate_HIV2_vs_HIV1* * *TB_mortality_rate_HIV0_vs_HIV2* if HIV-

These rates are increased by a factor of *relative_TB_mortality_rate_chronic* for individuals with chronic conditions.

In the community-based intervention scenarios, individuals can also move from the subclinical and clinical TB compartments to the treatment compartment through community active case finding. Details are given in Section 1.1.13.1.

1.1.11.3 Mycobacterium tuberculosis transmission

Mtb transmission occurred on a monthly time step in the model. For each susceptible or latent individual in the model, the probability of infection each month was calculated as:

$$1 - \prod_{c=0}^{1} \prod_{h=0}^{2} (1 - transmission\_prob \times w_c \times v_h \times reinfection\_relative\_risk)^{N_{ch}})$$

Where:

- c indicates the symptom status of individuals with TB (0 = no symptoms caused by TB or chronic conditions, 1 = symptoms caused by chronic conditions only, 2 = symptoms caused by TB), h indicates their HIV/ART class (0 = HIV negative, 1 = HIV positive, not on ART, 2 = HIV positive, on ART), and N_{ch} indicates the number of people with TB with a particular symptom state and HIV/ART status.
- w_c =
  - *reduced_transmission_sub* when c = 0
  - *(transmission_multiplier_chronic * 0.587 + reduced_transmission_sub * (1 – 0.587))* when c = 1
  - 1 when c = 2
- v_h =
  - 1 when h = 0
  - *reduced_transmission_HIV1_vs_HIV2 * reduced_transmission_HIV2_vs_HIV0* when h = 1
  - *reduced_transmission_HIV2_vs_HIV0* when h = 2
- *reinfection_relative_risk* =
  - 1 if the individual was uninfected
  - *reinfection_relative_risk_HIV0* if they were HIV- and latently infected
  - *reinfection_relative_risk_HIV1* if they were HIV+ART- and latently infected
  - *reinfection_relative_risk_HIV2* if they were HIV+ART+ and latently infected.

1.1.11.4 Test sensitivities and specificities

1.1.11.4.1 Symptom screen

We assume that a symptom screen has a sensitivity of 100% for people with TB symptoms caused by TB and/or by chronic conditions, and for people visiting clinics seeking care for TB symptoms caused by transient conditions. We assume that all other people have a probability of reporting TB symptoms when asked (i.e. due to transient conditions) which depends on their HIV/ART status and whether they are a clinic attendee or a community screening participant. For screening conducted in the community, the probability of reporting symptoms due to transient conditions, *prob_transient_comm_hiv0/1/2* is calculated as:

- If HIV-negative: (*prop_with_symp_hiv0* - *prop_with_symp_hiv0* * *proportion_symp_chronic_hiv0*)/(1 - *prop_with_symp_hiv0* * *proportion_symp_chronic_hiv0*)
- If HIV-positive: (*prop_with_symp_hiv1/2* - *prop_with_symp_hiv1/2* * *proportion_symp_chronic_hiv12*)/(1 - *prop_with_symp_hiv1/2* * *proportion_symp_chronic_hiv12*)

For screening conducted in clinic, the probability of reporting symptoms, *prob_transient_clinic_hiv0/1/2* is calculated as:

- If not on ART: (*prob_transient_comm_hiv0/1* * *clinic_other_symp_hiv01* / (*prob_transient_comm_hiv0/1* * *clinic_other_symp_hiv01* + (1 - *prob_transient_comm_hiv0/1*)))
- If on ART: (*prob_transient_comm_hiv2* * *clinic_other_symp_hiv2* / (*prob_transient_comm_hiv2* * *clinic_other_symp_hiv2* + (1 - *prob_transient_comm_hiv2*)))
    - where *clinic_other_symp_HIV2* = (1 + (*clinic_other_symp_HIV01* - 1) * *clinic_other_HIV2_vs_HIV01*)

1.1.11.4.2 Ability to produce sputum

Limited data are available on the proportion of people who are able to produce sputum samples in typical routine screening conditions in clinics, however it is likely to be substantially lower than the proportions achieved by prevalence surveys and research studies. One study in Botswana found that, before an intervention, only 44.1% of people starting ART were able to provide at least one sputum sample[26]. A study of clinic attendees in KwaZulu-Natal, South Africa found that only 50.4% were able to produce sputum (while this was a research study, the sputum collection procedures were in line with those used routinely in the clinics)[15]. The majority of people in these studies were not found to have TB, and we therefore assumed that 44.1 – 50.4% of people without TB are able to produce sputum (*produce_sputum_prob_tb*). A community prevalence survey in the same setting in

South Africa found little difference in the proportion of people able to produce sputum by sex, HIV/ART status or by reported TB symptoms[8], and we therefore assumed that the proportion of people able to produce sputum did not vary by these characteristics.

The proportion of people able to produce sputum in routine clinic conditions may be higher for people with TB. Following an intervention, the proportion of people able to produce sputum in the study in Botswana increased from 44.1% to 58.3%, and the overall yield of TB increased from 9.7% to 12.1%[26]. The prevalence of TB in people able to produce a sputum sample varied little between before and after the intervention. Using these data, we estimated that the proportion of people with TB who are able to produce sputum in routine clinic settings (*produce_sputum_prob_notb*) is at most 79.9% (assuming that all TB was detected following the intervention), and at least 48.4% (assuming that the prevalence of TB in people unable to produce sputum following the intervention was the same as in people able to produce sputum following the intervention). In line with people without TB, we assumed that the ability of people with TB to produce sputum does not vary by sex, HIV/ART status, or symptoms.

We assumed that the proportion of people with TB who are able to produce sputum could not be lower than the proportion of people without TB who are able to produce sputum.

1.1.11.4.3 Xpert Ultra

Two Cochrane systematic reviews of the sensitivity and specificity of Xpert have been conducted, finding the sensitivity of Xpert Ultra to be 90.9% (95% CI 86.2% – 94.7%) in adults with presumptive pulmonary tuberculosis[27] and 69% (95% CI 57% – 80%) in people living with HIV "irrespective of signs or symptoms"[28]. We therefore simulated a range for the sensitivity of Xpert Ultra in people seeking care for TB symptoms (passive case finding) of 86.2% – 94.7%. The second review found no studies of screening in the general population, however the estimated sensitivity varied little between people living with HIV and non-hospitalised people in high risk groups, and we therefore assumed that the sensitivity was the same for HIV- people as for HIV+ people, and simulated a range for the sensitivity of Xpert Ultra used for active case finding in clinics or the community of 57% – 80%.

Systematic reviews have estimated that the specificity of Xpert Ultra is 95.6% (93.0% to 97.4%) in people with presumptive TB[27] and 98% (97% to 99%) in PLWHIV irrespective of signs and symptoms[28], with the latter estimate coming from only a single conference abstract. We simulated a wide plausible range of 93.0% - 99.0% for the specificity of Xpert Ultra in all populations, encompassing the ranges from both reviews.

1.1.11.4.4 Xpert Ultra, saliva samples

A recent systematic review summarising studies estimating the sensitivity and specificity of different nucleic acid amplification tests (including Xpert Ultra) using different oral samples[29]. Only two studies estimated sensitivity for Xpert using saliva samples, finding sensitivities of 90% (95% CI 81% – 95%) using Xpert Ultra[30] and 38.5% (22.4% - 57.5%) using Xpert MTB/RIF[31]. All participants were sputum smear positive in the former study however, and 87% in the latter, and in the latter study participants were people being investigated for presumptive TB. These estimates may therefore be overestimates of the sensitivity when used for passive case finding in people unable to produce sputum. We therefore used the estimate from the systematic review for the sensitivity of Xpert MTB/RIF or Xpert Ultra using any oral specimen type, of 30% (95% CI 8% – 51%).

We assumed that Xpert Ultra using saliva samples has the same specificity as Xpert Ultra using sputum samples, giving a range of 93% - 99%, in line with the overall estimated specificity of nucleic acid amplification tests using oral samples[29]

1.1.11.4.5 Chest X-ray

We assume that chest X-ray has a sensitivity of 94% (95% CI: 92% – 96%) and a specificity of 89% (95% CI: 85% – 92%), in line with WHO estimates of the sensitivity and specificity of chest X-ray (any abnormality) for screening for TB in the general population[32].

1.1.11.5 Initial loss to follow up

We assume that 11 – 18% of people are lost to follow up between diagnosis and starting treatment[33]. We assume that 50% of people diagnosed using Xpert Ultra in clinics or in community screening approaches and lost to follow up will be identified and start treatment at their next clinic visit, provided it occurs with two years of their diagnosis. We assume that the other 50% would need to be diagnosed again, e.g. because they attend a different clinic. We assume that people diagnosed following referral for X-ray or a doctor would need to be diagnosed again if lost to follow up, as we assume that in the majority of cases their local clinic would not be aware of their diagnosis.

1.1.11.6 Treatment

1.1.11.6.1 People with TB (true-positives)

Treatment was assumed to last for 6 months in the model. Individuals successfully finishing treatment re-entered the latent stage with an effective time since infection of 6 months, reflecting the high rates of disease recurrence following treatment[34,35].

Individuals receiving TB treatment died of TB at a rate of *TB_mortality_rate_treatment_hiv0/12* and discontinued treatment at a rate of *TB_treatment_dropout_rate_hiv0/12*. Upon discontinuation,

they returned to the subclinical TB compartment. Values of *TB_mortality_rate_treatment_hiv0/12* and *TB_treatment_dropout_rate_hiv0/12* were chosen to allow the model to match data on TB treatment outcomes for HIV+ and HIV- people in South Africa in 2020[36].

1.1.11.6.2 People without TB (false-positives)

Individuals starting treatment who did not have TB are assumed to have the same rates of treatment discontinuation as people with TB. They are assumed to have no risk of becoming (re)infected with TB or developing disease whilst receiving treatment. After ending treatment (through successfully finishing or discontinuing), it is assumed that they are not at risk of developing TB without (re)infection.

1.1.11.7 Prevalence of infection in 15-year olds

An empirical study has estimated that the prevalence of *Mtb* infection in 12 – 17 year olds in KwaZulu-Natal province, South Africa in 2018 was 20.6%[37]. Upon being created at the age of 15 years, people in the model therefore set their state to latent with probability *prop_infect_15* = 0.206. The remaining people were assumed to be uninfected.

In calculating rates of progression to active disease in individuals with *Mtb* infections at the point of their creation at age 15 in the model, we assigned them a time of infection, *time_of_infection*, from a uniform distribution covering the 15 years before their creation. Their rate of disease progression following creation was then calculated using the same method as was used for people infected at ages ≥15 years. Progression to disease that occurred prior to the age of 15 was not included in the model.

1.1.11.8 Changes in TB parameters over time

To reflect secular trends not captured by other time varying parameters in the model (for instance, improvements in treatment coverage, and improvements in nutrition and housing), a change was modelled between *tb_rates_change_start_year* and *tb_rates_change_end_year*. Starting from *tb_rates_change_start_year*, the values of the simulated rate of *Mtb* transmission (*transmission_prob*) and the simulated rates of progression to TB disease following infection were reduced linearly, reaching values *relative_reduction_rates* times their original values in *tb_rates_change_end_year*.

1.1.11.9 Calibration targets

The model was calibrated to WHO estimates/data[36] for South Africa of:

- Estimated adult TB incidence and HIV+ TB incidence in 2005 and 2023. WHO estimates of overall incidence by year and incidence by age and HIV status in 2023 were used to estimate

adult incidence, assuming that the ratio of TB incidence in adults compared to children was the same in 2005 as in 2023, and that the relative increase in incidence in HIV positive adults compared to HIV negative adults is the same as the relative increase in incidence in HIV positive children compared to HIV negative children.

- Estimated TB mortality and HIV+ TB mortality in 2005 and 2023. WHO estimates of overall (adult and child) mortality were adjusted to give estimated mortality in adults only, assuming that the proportion of people (overall and HIV positive) who develop TB who die of the disease is the same for adults as for children
- TB notification rates in adults in 2018 and 2023. We fitted the model to calibration targets ranging from the reported rates -10% to +10% in 2023, and -10% to +30% in 2018. A higher upper bound was used for 2018, as we did not explicitly model the scale up of active case finding in people on ART in clinics, and therefore numbers of false positive diagnoses in the model in 2018 may be too high.
- The ratio of number of people starting TB treatment to estimated TB incidence in 2023
- The proportion of adults starting TB treatment in 2023 who were living with HIV. Plausible bounds were calculated assuming that all adults who were not tested were or were not HIV positive.
- The proportion of TB deaths that occur in people who are on TB treatment in 2022, by HIV status. Calculated from TB treatment outcome data and estimated TB mortality numbers in 2022. Ranges were widened by ±30% for HIV- people, due to the unrealistically low uncertainty around HIV- TB mortality in WHO estimates.

The model was also calibrated to data from the 2017-2019 South Africa TB prevalence survey[3], fitting model output in 2018 to estimates of:

- The prevalence of bacteriologically positive TB in adult men and women
- The estimated prevalence of TB in HIV- and HIV+ adults
- The proportion of HIV- and HIV+ adults with prevalent bacteriologically positive TB who report TB symptoms
- The proportion of all HIV+ adults who report TB symptoms

Published estimates of TB prevalence in both HIV+ and HIV- adults (1734 and 900 per 100,000 respectively) are higher than the overall published estimate (852 per 100,000), due to a low TB prevalence in people with unknown HIV status. We therefore decreased the lower bounds estimated prevalence in HIV- and HIV+ adults, by multiplying them by the ratio of the adjusted overall TB prevalence in the population to the adjusted prevalence of TB in people with known HIV status.

The prevalence of TB symptoms in all participants and participants with bacteriologically positive TB in the South Africa national TB prevalence survey broken down by HIV status are shown in **Error! Reference source not found.**. The prevalence of reported symptoms was lower in people with unknown HIV status than in participants with known HIV status, both in all participants and in participants with bacteriologically positive TB. We therefore adjusted the lower bounds for symptom prevalence in HIV+ and HIV- people, assuming that all participants with unknown HIV status were HIV+ or HIV- respectively. The ranges may also be overestimates of the true proportions of people with TB symptoms due to selection bias, as only 66% of eligible people participated in the prevalence survey, and it is likely that participation was higher among people with symptoms. A community TB prevalence survey conducted in Kwa-Zulu Natal also found a much lower prevalence of TB symptoms: 8.5% among all participants, and 18.2% among participants with bacteriologically positive TB[8,15]. We therefore reduced the lower bounds by a further 40%. The reduction in the lower bounds for the estimates for people with bacteriologically positive TB also allows for the potential underestimation of the proportion who are symptom-screen negative resulting from only people with a positive chest X-ray or reporting symptoms being tested[7].

Table 1 Prevalence of reported symptoms by HIV status, overall and in people with bacteriologically positive TB, in the South Africa TB prevalence survey, and the ranges used to calibrate the model

	HIV status	Prevalence of TB symptoms		
		Crude (95% CI)	Range adjusted for unknown HIV status	Final range used to calibrate model
All participants	HIV+	22.2% (21.0 – 23.4%)	15.0 – 23.4%	9.0 – 23.4%
	HIV-	14.2% (13.7 – 14.6%)	13.2 – 14.6%	7.9 – 14.6%*
	HIV unknown	12.0% (11.3 – 12.7%)		
Participants with bacteriologically positive TB	HIV+	56.4% (42.3 – 69.7%)	35.0 – 69.7%	18.2 – 69.7%
	HIV-	39.0% (30.7 – 47.7%)	30.0 – 47.7%	21.5% – 47.7%
	HIV unknown	32.6% (19.1 – 48.5%)		

*The prevalence of TB symptoms in HIV- people in the overall population is used as an input parameter, not a calibration target. The prevalence of TB symptoms in HIV+ people in the model is a calibration target, as it is a weighted average of the prevalence in HIV+ people who are and are not on ART.

1.1.12 Infection prevalence in child household contacts

Data were available from the AHCoS study on the prevalence of positive IGRA tests in child household contacts of people diagnosed with TB, and children living in households where no adults had been diagnosed with TB during the children's lifetimes ('controls')[38]. The mean age of children in the study/control households was 9 years.

The community force of infection each month over each the preceding nine years is tracked in the model in the form of a list *FOI_list* with 9 × 12 elements. The proportion of control children infected in 2023, *prop_infected_control*, is estimated in the model as:

$$1 - \prod_{y \in FOI\_list} 1 - child\_transmission\_multiplier \times FOI_y$$

Where *child_transmission_multiplier* is a parameter to account for the fact that 1) children may be exposed to a lower or higher force of infection from community exposure than adults, and 2) the FOI may be different in the study community than in South Africa as a whole.

People with bacteriologically positive TB are assumed to expose their child household contacts to an additional household force of infection. This is tracked each month over a period of up to nine years, using a list (*FOI_hh_list*) for each person with TB. Each month, the risk of household transmission is calculated as:

$$1 - (1 - transmission\_prob \times w_c \times v_h)^{infectiousness \times hh\_transmission\_multipler}$$

Where:

- *c* indicates their symptom status (0 = no symptoms caused by TB or chronic conditions, 1 = symptoms caused by chronic conditions only, 2 = symptoms caused by TB) and *h* indicates their HIV/ART class (0 = HIV negative, 1 = HIV positive, not on ART, 2 = HIV positive, on ART).
- w_c =
    - *reduced_transmission_sub* when c = 0
    - *transmission_multiplier_chronic * 0.587 + reduced_transmission_sub * (1 – 0.587)* when c = 1
    - 1 when c = 2
- v_h =
    - 1 when h = 0
    - *reduced_transmission_HIV1* when h = 1
    - *reduced_transmission_HIV2* when h = 2

- infectiousness is an individual-level parameter, sampled at the start of the TB episode from a gamma distribution with mean 1 and dispersion parameter (*infectiousness_var*) k = 0.15[39,40].
- *hh_transmission_multiplier* reflects the increased exposure to the person with TB experienced by household members

When someone is diagnosed with TB in the model, the mean proportion of their child household contacts infected is calculated as:

$$1 - \prod_{y \in FOI\_list} 1 - child\_transmission\_multiplier \times FOI_y \prod_{y \in FOI\_hh\_list} 1 - FOI\_hh_y$$

The model was calibrated to the data from the AHCoS study on:

- The proportion of children infected in control households
- The proportion of children infected in child household contacts of HIV- people diagnosed with TB
- The relative risk of infection in child household contacts of people diagnosed with TB who are HIV+ART+ vs HIV-
- The relative risk of infection in child household contacts of people diagnosed with TB who are HIV+ART- vs HIV-
- The relative risk of infection in child household contacts of HIV+ART+ people diagnosed with TB who had symptom-screen negative vs symptom-screen positive TB at the time of diagnosis

1.1.13 Interventions

All intervention approaches were simulated from the start of 2026, and run for a total of 10 years.

1.1.13.1 Community-based

Three community-based active case finding intervention approaches were simulated. We assumed that the interventions would be targeted at areas thought to have a high prevalence of TB. No data are available on how TB prevalence varies between communities across South Africa. We therefore used district level notification data from 2022[13] to provide a rough indication of how much higher prevalence may be in areas targeted for screening (compared to the country as a whole), and how this may vary with the proportion of the population targeted (**Error! Reference source not found.**). Based on this relationship, we simulated scenarios covering 10%, 20%, 40%, 60%, and 80% of the

adult population of South Africa, targeted at areas with true prevalences of TB 2.27, 1.91, 1.56, 1.35, and 1.20 times higher respectively than the prevalence in the country as a whole.

Figure 3 Relationship between the proportion of the population of South Africa, and the notification rate in that group relative to the notification rate in South Africa as a whole The leftmost point indicates the proportion of the population living in the district with the highest notification rate in 2022 and the notification rate in that district compared to the notification rate in South Africa as a whole. The second point shows data for the two highest notification rate districts, the third the three highest, etc.

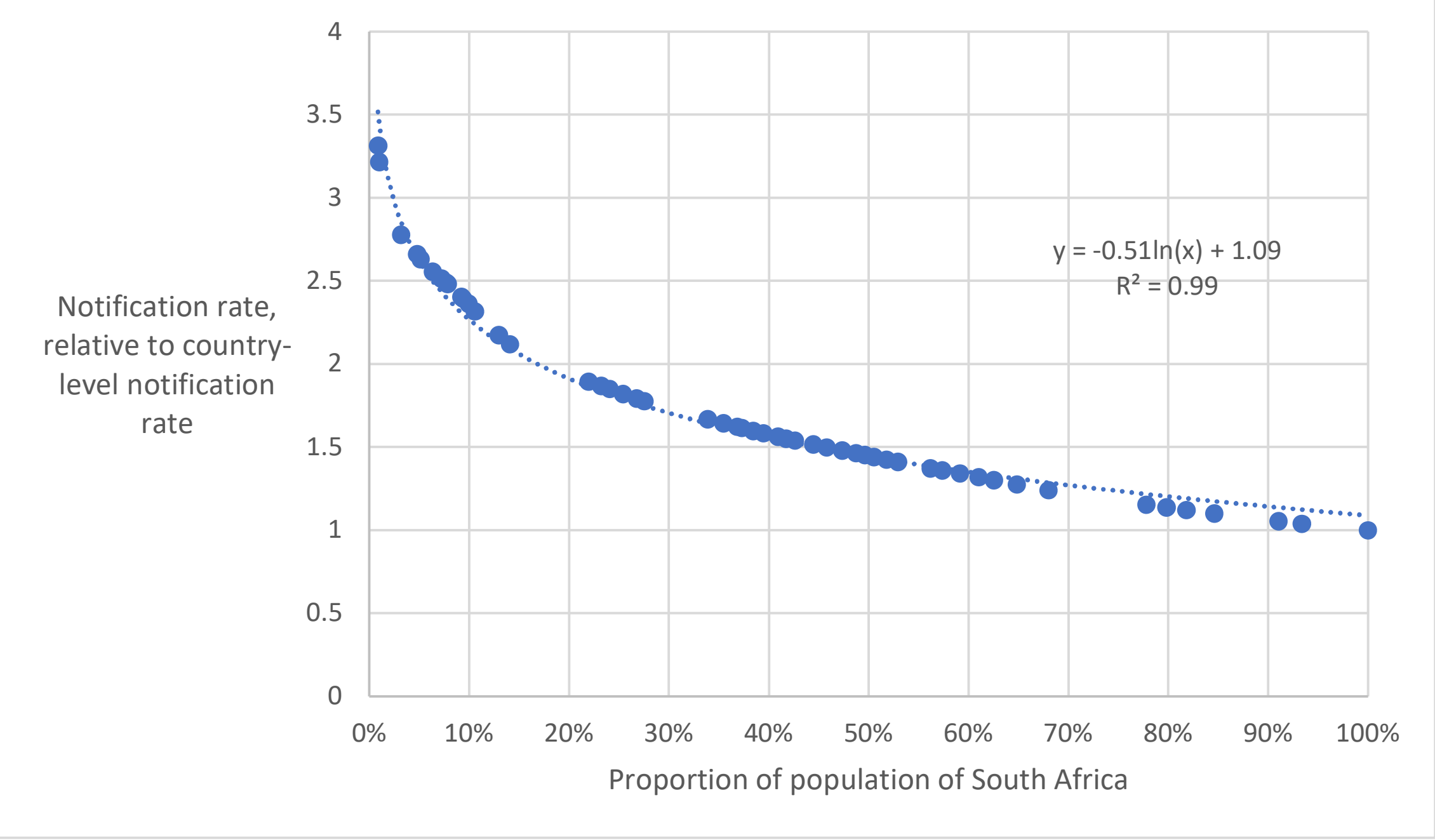

Each simulated individual was assigned a screening month at random at birth. Each month from the start of the intervention, people with the corresponding assigned screening month would be screened with probability:

- (*comm_int_cov* / (*TB_prevalence* + (1 - *TB_prevalence*) / *comm_RR_TB*))
  If they have TB and are not on TB treatment
- (*comm_int_cov* / (*comm_RR_TB* * *TB_prevalence* + 1 - *TB_prevalence*))
  If they do not have TB and are not on TB treatment

Where *TB_prevalence* is the prevalence of untreated TB in the model at the start of that month, *comm_int_cov* is the proportion of the population reached each year by the intervention, and *comm_RR_TB* is the true prevalence of TB in people screened.

Details of the simulated screening algorithms for the community-based approaches are shown in Supplementary material 2.

1.1.13.2 Clinic-based

Three clinic-based active case finding intervention approaches were simulated. These all included modifications to improve the active-screening pathway for HIV+ people on ART, and included the addition of active screening in some or all HIV- people and HIV+ people who are not on ART. The probability of diagnosis through passive case finding for people seeking care for TB symptoms remained the same as baseline, and was assumed to occur after any screening through active case finding had occurred. Full details of the simulated screening algorithms for the clinic-based approaches are shown in Supplementary material 2.

1.1.13.2.1 Intensified targeted universal testing and treatment (TUTT)

TUTT is an approach where screening with Xpert Ultra is offered to all clinic attendees in three key risk groups: people living with HIV (PLWHIV), people reporting recent TB (within prior two years) and people reporting recent close contact with a person with TB (within past year)[41].

We considered it plausible that most PLWHIV who are not on ART either do not know their status, or would be reluctant to disclose it to healthcare workers as part of TB screening. We therefore assumed in the model that screening was limited to PLWHIV who are on ART only, unless they were also part of one of the other risk groups. We assumed that 95% of people on ART would report that they were HIV positive, with the remaining 5% not receiving screening. We tracked the date at which people last started TB treatment, with people who started treatment within the past two years (and who were no longer on treatment) eligible for testing. We assumed that 100% of people with recent TB would report their recent TB and receive screening.

Contacts of people with TB were not explicitly modelled, and were therefore selected at random from people visiting a clinic who did not meet the criteria for recent TB, with the probability of selection varying over time and with their true TB status. In line with the findings from a trial of the TUTT intervention, we assumed that the true prevalence of TB in recent close contacts is 40% lower than the true prevalence in people with recent TB[42]. Eight times as many people reported recent contact as reported recent TB in the trial, which we considered implausible in a scaled-up intervention. We therefore assumed that twice as many people would report being a recent contact as would report having recent prior TB.

We modelled a minimum time between receiving an Xpert through active screening of six months.

We assumed that people not on ART would be tested for HIV as part of the screening process, and would start ART if they tested positive.

1.1.13.2.2 Intensified TUTT allowing saliva samples

This approach is the same as the intensified TUTT approach described above, except that the pathway for people unable to produce a sputum sample was different. We assumed that people unable to produce a sputum sample would produce a saliva sample, which would be tested with Xpert Ultra, with a reduced sensitivity as described in Section 1.1.11.4.4.

1.1.13.2.3 Clinic-based symptom screening

The probability of symptom screening occurring for people on ART who had an Xpert Ultra attempted within the past year was increased from *symptom_screen_prob* (with baseline values of 0.5 – 0.7) to 0.75. The probability of symptom screening occurring for people not on ART was increased from 0 to 0.75. The proportion of symptomatic people on ART who have received an Xpert Ultra 6 months to 1 year ago and who are unable to produce sputum who see a doctor is increased from 65% to 75%.

1.1.14 Calibration

The model was calibrated using history matching and emulation, using the R package *hmer*[43]. As we were modelling an entire country, the model was treated as deterministic, with results for each parameter set averaged over 200 runs. Calibration was performed iteratively as the model was developed, with the ranges used for parameters that are not informed by data or expert opinion (i.e. those where the only constraints are those imposed by calibrating the model to data) modified each time the model was recalibrated to ensure that a) ranges were wide enough to contain all possible full fitting points and b) ranges were not unnecessarily wide, as this would increase the number of waves required for calibration and increase the risk of full-fitting space being incorrectly ruled out.

A number of input parameters were transformed, to allow the distribution of the values of other input parameters or calibration targets in the fitting points to better reflect the desired distributions.

The model used the parameter *transmission_prob* as the baseline rate of transmission between each infectious and susceptible person (see Section 1.1.11.3), with *transmission_prob* multiplied by *reduced_transmission_sub* for infectious people with subclinical TB and no chronic condition. The value of *transmission_prob* was calculated from the input parameter *baseline_infection_rate* as *transmission_prob* = 1 / *baseline_infection_rate*. This transformation was used because, when all else is equal, there is a non-linear relationship between the values of *transmission_prob* and the values of *reduced_transmission_sub* that give rise to any particular average rate of transmission

between each infectious and susceptible person (**Error! Reference source not found.**a). As *hmer* treats the distributions of all input parameters as uniform, this would result in the proportion of the multi-dimensional fully fitting parameter space that contains smaller values of *reduced_transmission_sub* being considerably larger than the proportion that contains larger values, and in smaller values of *reduced_transmission_sub* being greatly overrepresented in the final full fitting parameter sets. Using the transformed variable results in a linear relationship between the values of *transmission_prob* and the values of *reduced_transmission_sub* that give rise to any particular average rate of transmission between each infectious and susceptible person (**Error! Reference source not found.**b), and prevents this source of bias.

Figure 4 Relationship between the values of a) transmission_prob and reduced_transmission_sub and b) baseline_infection_rate and reduced_transmission_sub that, all else being equal, give rise to any particular average rate of transmission between each infectious

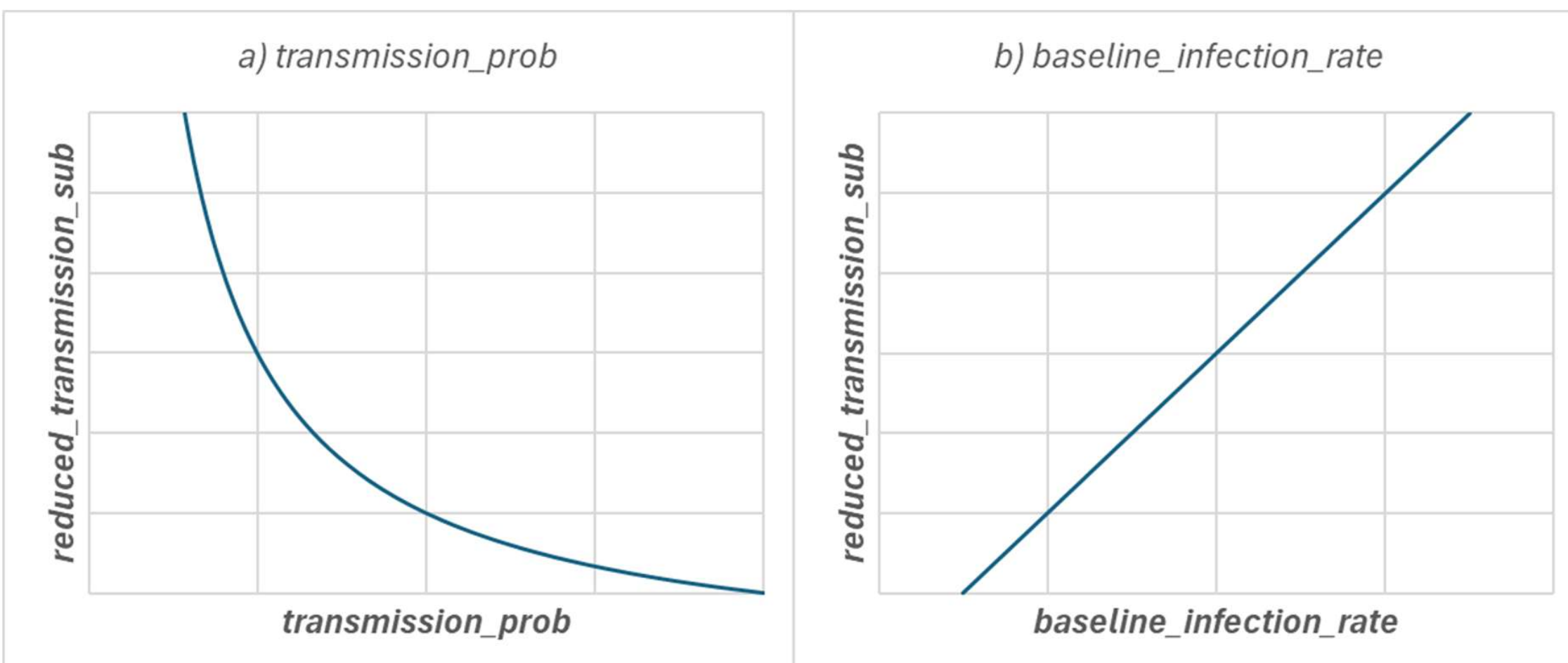

The parameters controlling the rates of starting ART in men and women in different time periods were also transformed. This was because the relationship between the ART start rates and the level of ART coverage was non-linear, with small changes in the values of the rates having a larger impact on ART coverage at the bottom of the ranges of the rates than at the top. This resulted in ART coverage being concentrated at the tops of the ranges in initial calibration attempts. The relationships between the start rates and ART coverage were determined using the fitting points from those early calibration attempts and used to identify transformations that would result in improved distributions of ART coverage in the final calibration. The transformations used were:

ART_start_rate_early_m_annual = 0.054 * exp(1.068 * ART_start_rate_early_m_annual_input)

ART_start_rate_early_f_annual = 0.062 * exp(1.61 * ART_start_rate_early_f_annual_input)

ART_start_rate_late_m_annual = 0.021 * exp(2.86 * ART_start_rate_late_m_annual_input)

ART_start_rate_late_f_annual = 0.0193 * exp(3.44 * ART_start_rate_late_f_annual_input) – 0.019

A total of 64 input parameters were varied during final calibration (**Error! Reference source not found.**), with the model calibrated to 42 calibration targets (**Error! Reference source not found.**). A total of 10 waves of calibration were run, with the first full fitting points found from wave 4. The yield of full fitting points was sufficiently high in wave 10 (18% full fitting), so we generated new points from wave 10 in sets of 2000 until a minimum of 1000 full fitting points were found. This gave us a total of 1041 full fitting points that were used in the analysis.

The model was run at 10 × the number of input parameters = 640 parameter sets to provide the training data for the emulators each wave. A further 5 × the number of input parameters = 320 parameter sets were used to validate and adjust the emulators, using the *hmer* package function *diagnostic_pass*. Finally, the emulators were validated using a further 9 hand fitted points near the edges of the full fitting space (i.e. at the lower or upper ends of the ranges for one or more input parameter, and/or at the lower or upper ends of the ranges for one or more calibration target). If any of the emulators ruled out any of these full fitting points, then their uncertainties (sigmas) were iteratively increased by 5% until the point was no longer ruled out.

1.1.15 Input parameters

1.1.15.1 Fixed input parameters

Table 2 Descriptions and values of input parameters that were not varied

**Parameter name**	**Description**	**Value**	**Source**
**Demography**			
min_age	Minimum age in model	15	Majority of *Mtb* transmission from people aged 15+, and interventions are focused on screening adults
max_age	Maximum age in model	80	<1.3% of adult population aged >80 in South Africa in 2021
min_background_mort_age_m	Minimum age from which non-TB and non-HIV mortality occurs in men in the model	40	Values chosen to give an approximate fit to the age distribution by sex in South Africa in 2021[2]
min_background_mort_age_f	Minimum age from which non-TB and non-HIV mortality occurs in women in the model	45	
background_mort_m	Annual non-HIV and non-TB mortality rate in men above min_background_mort_age_m	0.0458	
background_mort_f	Annual non-HIV and non-TB mortality rate in women above min_background_mort_age_f	0.0355	
prop_male	Proportion of newly created people who are male	0.476	Demographic estimates[2]
**HIV and ART**			

hiv_intro_year	Year HIV first introduced into the model	1995	Chosen to allow the model to fit to trends in HIV prevalence and ART coverage, and in TB incidence
art_intro_year	Year ART first introduced into the model	2010	
HIV1_mortality_rate_annual	Annual non-TB HIV mortality rate in HIV+ people not on ART	0.00225	See Section 1.1.10
HIV2_mortality_rate_annual	Annual non-TB HIV mortality rate in HIV+ people on ART	0.00108	
hiv_inc_change_year1_start	Year first change in HIV incidence starts	2000	Chosen to allow the model to fit to trends in HIV prevalence and ART coverage
hiv_inc_change_year1_end	Year first change in HIV incidence ends	2020	
hiv_inc_change_year2_start	Year second change in HIV incidence starts	2023	
hiv_inc_change_year2_end	Year second change in HIV incidence ends	2040	
ART_start_rate_change_year	Year change in ART start rates occurs	2017	
abs_increased_prop_symp_chronic_hiv	Absolute increase in the proportion of TB symptoms that are due to chronic conditions in people who are HIV+	0.1	Expert opinion, see Section 1.1.7.2
**Clinic visiting**			
clinic_visit_alpha_m_hiv01	Parameters controlling the distributions for the individual-level rates at which men and women who are on or not on ART visit clinics, for reasons other than seeking care for TB symptoms	0.149	See section 1.1.9.2.1.1
clinic_visit_lambda_m_hiv01		0.186	
clinic_visit_alpha_m_hiv2		1.75	
clinic_visit_lambda_m_hiv2		0.444	
clinic_visit_alpha_f_hiv01		0.368	
clinic_visit_lambda_f_hiv01		0.198	
clinic_visit_alpha_f_hiv2		1.93	

clinic_visit_lambda_f_hiv2		0.416	
**TB diagnosis and treatment**			
TB_mortality_rate_treatment_hiv0	Rate of dying of TB on TB treatment if HIV- negative, per month	0.0131	Data on TB treatment outcomes for HIV+ and HIV- people in South Africa in 2020[36]
TB_treatment_dropout_rate_hiv0	Rate of stopping TB treatment if HIV- negative, per month	0.0263	
TB_mortality_rate_treatment_hiv12	Rate of dying of TB on TB treatment if HIV+ negative, per month	0.0169	
TB_treatment_dropout_rate_hiv12	Rate of stopping TB treatment if HIV+ negative, per month	0.0180	
**Mtb transmission and TB development**			
reinfection_relative_risk_HIV0	Relative risk of reinfection compared to initial infection, in HIV-, HIV+HIV-, and HIV+ART+ people	0.21	Andrews *et al* 2012[44]
reinfection_relative_risk_HIV1		0.75	Dowdy and Chaisson, 2009[45]
reinfection_relative_risk_HIV2		0.48	Intermediate between protection in HIV- and HIV+ART-
develop_tb_y1_rate_HIV0_annual	Annual rate of developing TB in HIV- people <1 year, 1-2 years, 2-3 years, 3-4 years, 4-5 years, and >5 years following last infection	0.0866	Kasaie 2014[46]
develop_tb_y2_rate_HIV0_annual		0.0355	
develop_tb_y3_rate_HIV0_annual		0.0112	
develop_tb_y4_rate_HIV0_annual		0.0074	
develop_tb_y5_rate_HIV0_annual		0.0024	
develop_tb_reactivation_rate_HIV0_annual		0.0005	
prop_infect_15	Proportion of people with *Mtb* infection at their creation at age 15	0.206	Mzembe 2020[37]

infectiousness_var	Individual-level infectiousness is sampled from a gamma distribution with mean 1 and dispersion parameter k = infectiousness_var	0.15	McCreesh and White 2018[39] and Smith *et al* 2023[40]
infection_seed_proportion	Proportion of people initially seeded with Mtb infection	0.7	NA
tb_seed_proportion	Proportion of people initially seeded with TB disease	0.001	NA

1.1.15.2 Input parameters varied during calibration

Table 3 Descriptions and ranges of input parameters that were varied during calibration

**Parameter name**	**Description**	**Range permitted for fitting points**	**Range (median) in fitted parameter sets**	**Type/source**
**HIV/ART**				
hiv_inc_early_f_annual	Parameters controlling HIV incidence in men and women in different time periods (see Section 1.1.10)	0 – 0.05	0.019 – 0.034 (0.025)	Constrained during calibration
hiv_inc_early_m_annual		0 – 0.05	0.013 – 0.025 (0.019)	
hiv_inc_mid_f_annual		0 – 0.04	0.0042 – 0.011 (0.0072)	
hiv_inc_mid_m_annual		0 – 0.03	0.00069 – 0.0063 (0.0038)	
hiv_inc_f_late_reduction		0.2 – 1	0.205 – 0.98 (0.508)	

hiv_inc_m_late_reduction		0.2 – 1	0.408 – 0.999 (0.665)	
art_intro_year	Year ART is introduced into the model	2005 – 2010	2005.0 – 2010.0 (2007.06)	Chosen to allow the model to fit to data on ART coverage
ART_start_rate_early_m_annual_input	Parameters controlling ART start rates (see Section 1.1.13.2.3 for details)	0 – 1	0.014 – 0.77 (0.44)	Constrained during calibration
ART_start_rate_early_f_annual_input		0 – 1	0.131 – 0.878 (0.53)	
ART_start_rate_late_m_annual_input		0 – 1	0.32 – 0.71 (0.53)	
ART_start_rate_late_f_annual_input		0 – 1	0.51 – 0.83 (0.69)	
***Mycobacterium tuberculosis* transmission**				
baseline_infection_rate	The baseline rate of transmission between each infectious and susceptible person in the model is a function of 1 / baseline_infection_rate. See sections Mycobacterium tuberculosis transmission1.1.11.3 and 1.1.13.2.3 for details	3000 – 30000	8260.7 – 15075 (11450)	Constrained during calibration
reduced_transmission_sub	Rate of transmission from people with subclinical TB and no chronic condition, relative to people with clinical TB	0.2 – 1.3	0.53 – 1.2 (0.83)	Expert opinion
transmission_multiplier_chronic	Parameter controlling rate of transmission from people subclinical TB and a chronic condition (see Section 1.1.7.2)	1.1 – 1.5	1.1 – 1.4 (1.3)	Expert opinion
reduced_transmission_HIV1_vs_HIV2	Rate of *Mtb* transmission from HIV+ART- people relative to HIV+ART+ people	0 – 1	0.31 – 0.93 (0.62)	Assume that HIV+ART- people cannot be more infectious than HIV+ART+ people

reduced_transmission_HIV2_vs_HIV0	Rate of *Mtb* transmission from HIV+ART+ people relative to HIV- people	0 – 1	0.29 – 0.98 (0.68)	Assume that HIV+ART+ people cannot be more infectious than HIV- people
hh_trans_rate_multiplier	Parameter controlling rate of transmission to child household contacts	0 – 2000	97 – 433 (232)	Constrained during calibration
child_transmission_multiplier	Parameter controlling rate of community transmission to children	0 – 0.4	0.097 – 0.32 (0.21)	Constrained during calibration
**Progression to disease**				
increased_develop_tb_rate_HIV2_vs_HIV0	Increased rate of developing TB following infection in HIV+ART+ people relative to HIV- people	1.47 – 10.8	1.66 – 4.25 (2.88)	Gupta *et al* 2012[18] and Gatechompol *et al* 2022[19]
increased_develop_tb_rate_HIV1_vs_HIV2	Increased rate of developing TB following infection in HIV+ART- people relative to HIV+ART+ people	1.89 – 4.76	2.48 – 4.6 (3.47)	Suthar *at al* 2015[20]
increased_TB_development_male	Increased rate of developing TB following infection in men compared to women	1 – 4	1.14 – 2.87 (1.88)	Constrained during calibration
increased_TB_development_chronic	Increased rate of developing TB following infection in people with chronic conditions that cause TB symptoms	1.3 – 1.9	1.33 – 1.83 (1.59)	Expert opinion
tb_rates_change_start_year	Years between which rates of *Mtb* transmission and TB disease development reduce, and magnitude of reduction (see Section 1.1.11.8)	2000 – 2007	2001.9 – 2006.9 (2004.8)	Ranges chosen to allow model to fit to trends in TB incidence over time
tb_rates_change_end_year		2007 – 2015	2008.7 – 2015.0 (2012.4)	
relative_reduction_rates		0.70 – 1.00	0.86 – 0.98 (0.91)	
**Disease progression and regression**				
self_cure_rate_HIV0	Rate of self cure per year for HIV- people with asymptomatic TB	0 – 0.8	0.068 – 0.28 (0.17)	Constrained during calibration

self_cure_rate_HIV1_vs_HIV2	Rate of self cure for HIV+ART- people with asymptomatic TB, relative to HIV+ART+ people	0 – 1	0.006 – 0.92 (0.46)	Assumed that rate of self cure must be lower in HIV+ART- people than HIV+ART+ people
self_cure_rate_HIV2_vs_HIV0	Rate of self cure for HIV+ART+ people with asymptomatic TB, relative to HIV- people	0 – 1	0.063 – 0.96 (0.54)	Assumed that rate of self cure must be lower in HIV+ART+ people than HIV- people
relative_self_cure_rate_chronic	Rate of self cure in people with chronic conditions that cause TB symptoms, relative to people with no chronic conditions that cause TB symptoms	0.53 – 0.77	0.54 – 0.76 (0.65)	Expert opinion
TB_mortality_rate_HIV0_vs_HIV2	TB mortality rate in HIV- people relative to HIV+ART+ people	0 – 1	0.24 – 0.86 (0.55)	Assumed that TB mortality rate must be lower in HIV- people than HIV+ART+ people
TB_mortality_rate_HIV1	TB mortality rate per year in HIV+ART- people	0.3 – 4	0.97 – 2.7 (1.7)	Constrained during calibration
TB_mortality_rate_HIV2_vs_HIV1	TB mortality rate in HIV+ART+ people relative to HIV+ART- people	0 – 1	0.17 – 0.69 (0.41)	Assumed that TB mortality rate must be lower in HIV+ART+ people than HIV+ART- people
relative_TB_mortality_rate_chronic	TB mortality rate in people with chronic conditions that cause TB symptoms, relative to people with no chronic conditions that cause TB symptoms	1.3 – 1.9	1.3 – 1.8 (1.5)	Expert opinion
rate_sub_to_clin_hiv0_vs_hiv2	Rate of developing TB symptoms caused by TB in HIV- people relative to HIV+ART+ people	0 – 1	0.27 – 0.8 (0.53)	Assume that rate must be lower in HIV- people relative to HIV+ART+ people
rate_sub_to_clin_hiv1	Rate of developing TB symptoms caused by TB, per year, in HIV+ART- people	0 – 15	4.8 – 13.1 (8.0)	Wide plausible range, as few data available (see Section 1.1.7.1)
rate_sub_to_clin_hiv2_vs_hiv1	Rate of developing TB symptoms caused by TB in HIV+ART+ people relative to HIV+ART- people	0 – 1	0.37 – 0.97 (0.6)	Assume that rate must be lower in HIV+ART+ people relative to HIV+ART- people
relative_rate_sub_to_clin_chronic	Rate of developing TB symptoms caused by TB in people with chronic conditions that cause TB symptoms relative to people without chronic conditions that cause TB symptoms	1.3 – 1.9	1.3 – 1.9 (1.6)	Expert opinion

rate_clin_to_sub_hiv0	Rate of losing TB symptoms caused by TB, per year, in HIV- people	0 – 12	2.95 – 11.7 (7.84)	Wide plausible range, as few data available (see Section 1.1.7.1)
rate_clin_to_sub_hiv2_vs_hiv0	Rate of losing TB symptoms caused by TB in HIV+ART+ people relative to HIV- people	0 – 1	0.32 – 0.97 (0.65)	Assume that rate must be lower in HIV+ART+ people relative to HIV- people
rate_clin_to_sub_hiv1_vs_hiv2	Rate of losing TB symptoms caused by TB in HIV+ART- people relative to HIV+ART+ people	0 – 1	0.059 – 0.95 (0.53)	Assume that rate must be lower in HIV+ART- people relative to HIV+ART+ people
relative_rate_clin_to_sub_chronic	Rate of losing TB symptoms caused by TB in people with chronic conditions that cause TB symptoms relative to people without chronic conditions that cause TB symptoms	0.526 – 0.769	0.54 – 0.74 (0.63)	Expert opinion
**Symptoms and clinic visiting**				
prop_with_symp_hiv0	Proportion of HIV- people reporting TB symptoms	0.079 – 0.146	0.08 – 0.12 (0.098)	South Africa TB prevalence survey[3], adjusted as described in Section 1.1.11.9
prop_with_symp_input_hiv1	Parameter controlling the proportion of HIV+ART- people reporting symptoms (see Section 1.1.9.2.2)	0 – 1	0.015 – 0.26 (0.1)	Constrained during calibration
prop_with_symp_input_hiv2	Parameter controlling the proportion of HIV+ART+ people reporting symptoms (see Section 1.1.9.2.2)	0 – 1	0.028 – 0.65 (0.27)	Constrained during calibration
proportion_symp_chronic_hiv0	Proportion of TB symptoms in people without TB that result from chronic conditions (opposed to transient conditions)	0.3 – 0.7	0.30 – 0.54 (0.42)	Expert opinion
clinic_TBsymp_HIV0	Rate of seeking care for TB symptoms caused by TB in HIV- people	0 – 6	2.2 – 4.6 (3.2)	Constrained during calibration
clinic_TBsymp_HIV1	Rate of seeking care for TB symptoms caused by TB in HIV+ART- people	0 – 6	1.4 – 4.2 (2.4)	Only partially constrained during calibration, as most calibration targets not broken down by ART status and people on ART can also start treatment through active case finding. Maximum rate of 6 per year therefore set
clinic_TBsymp_HIV2	Rate of seeking care for TB symptoms caused by TB in HIV+ART+ people	0 – 6	0.45 – 5.6 (2.4)	

clinic_TBsymp_adjust_chronic	Rate of seeking care for TB symptoms in people whose TB symptoms are caused by chronic conditions only, compared to people with TB symptoms caused by TB	0.5 – 2	0.51 – 1.5 (0.93)	Expert opinion
clinic_TBsymp_adjust_transient	Rate of seeking care for TB symptoms in people whose TB symptoms are caused by transient conditions only, compared to people with TB symptoms caused by chronic conditions	0 – 1	0.01 – 0.97 (0.4)	Considered plausible that the rate of seeking care for TB symptoms caused by transient conditions would be lower than the rate of seeking care for TB symptoms caused by chronic conditions
clinic_visit_adjust	Parameter scaling the rates of clinic visiting for reasons other than seeking care for TB symptoms	0.5 – 1.5	0.88 – 1.1 (1.0)	Constrained during calibration
clinic_other_symp_HIV01	Increased rate of clinic visiting for reasons other than seeking care for TB symptoms, in people not on ART with TB symptoms	1 – 3	1.0 – 2.1 (1.5)	Constrained during calibration
clinic_other_tb_HIV01	Increased rate of clinic visiting for reasons other than seeking care for TB symptoms, in people not on ART with TB	1 – 15	1.1 – 4.9 (3.1)	Constrained during calibration
clinic_other_HIV2_vs_HIV01	Reduction in increased rates of clinic visiting for reasons other than seeking care for TB symptoms, for people on ART compare to people not on ART	0 – 1	0.048 – 0.77 (0.41)	Considered plausible that it would be lower in people on ART, due to the higher rate of clinic visiting for routine HIV care
**Diagnosis**				
passive_diagnosis_prob	Probability of diagnosis through passive case finding for people seeking care for TB symptoms	0.25 – 0.75	0.36 – 0.71 (0.49)	McCarthy *et al* 2018[47] and Foster *et al* 2015[48]
produce_sputum_prob_tb	Probability that a person with TB will be able to produce sputum	0.48 – 0.80	0.56 – 0.75 (0.64)	See Section 1.1.11.4.2
xpert_sens_passive	Sensitivity of Xpert Ultra in people seeking care for TB symptoms (passive case finding)	0.862 – 0.947	0.58 – 0.8 (0.69)	Zifodya 2021[27]

xpert_sens_active	Sensitivity of Xpert Ultra used for active case finding	0.57 – 0.80	0.86 – 0.94 (0.9)	Shapiro 2021[28]
symptom_screen_prob	Probability of symptom screening occurring during active case finding (see Section 1.1.9.4)	0.5 – 0.7	0.51 – 0.7 (0.61)	Expert opinion
xray_sens	Sensitivity of chest X-ray	0.92 – 0.96	0.92 – 0.95 (0.94)	World Health Organization 2021[32]
iltfu	Proportion of people lost to follow up between diagnosis and starting treatment	0.11 – 0.18	0.11 – 0.17 (0.13)	Mwansa-Kambafwile 2020[33]
produce_sputum_prob_notb	Probability that a person without TB will be able to produce sputum	0.44 – 0.50	0.44 – 0.5 (0.47)	Mathebula 2020[26]
xpert_spec	Specificity of Xpert Ultra	0.93 – 0.99	0.966 – 0.99 (0.983)	Zifodya 2021[27] and Shapiro 2021[28]
xray_spec	Specificity of chest X-ray	0.85 – 0.92	0.85 – 0.92 (0.88)	World Health Organization 2021[32]

1.1.15.3 Input parameters varied in future projections

Table 4 Descriptions and ranges of input parameters that were varied during the intervention runs

**Parameter name**	**Description**	**Range and distribution**	**Source**
xpert_saliva_sens	Sensitivity of Xpert Ultra with a saliva sample	Uniform distribution 0.08 – 0.51	Wang 2023[29]

1.1.16 Calibration targets table

Table 5 Calibration targets, ranges, and fitting values

Target description	Range	Range (median) in fitting points	Source
**HIV prevalence and ART coverage**			
Male adult HIV prevalence in 2000	0.061 – 0.13	0.061 – 0.11 (0.082)	UNAIDS estimates[21]
Female adult HIV prevalence in 2000	0.087 – 0.17	0.087 – 0.15 (0.12)	
Male adult HIV prevalence in 2023	0.11 – 0.13	0.11 – 0.13 (0.12)	
Female adult HIV prevalence in 2023	0.19 – 0.23	0.19 – 0.23 (0.21)	
Male adult HIV prevalence in 2030	0.077 – 0.14	0.085 – 0.12 (0.1)	Estimates from the Thembisa model[23], with widened ranges (see Section 1.1.10.2)
Female adult HIV prevalence in 2030	0.14 – 0.26	0.17 – 0.23 (0.2)	
Male adult ART coverage in 2016	0.37 – 0.67	0.41 – 0.65 (0.54)	UNAIDS estimates[21]
Female adult ART coverage in 2016	0.46 – 0.82	0.47 – 0.81 (0.68)	
Male adult ART coverage in 2023	0.65 – 0.77	0.66 – 0.77 (0.72)	
Female adult ART coverage in 2023	0.75 – 0.88	0.77 – 0.88 (0.84)	
Ratio of ART coverage in 2019 vs 2023 in adult men	0 – 1	0.8 – 0.97 (0.89)	Target used to exclude otherwise fitting runs where ART coverage declines following 2023
Ratio of ART coverage in 2019 vs 2023 in adult women	0 – 1	0.83 – 0.99 (0.92)	Target used to exclude otherwise fitting runs where ART coverage declines following 2023

TB incidence, mortality, and notifications			
TB incidence/100,000 in 2005	598 – 3423	946 – 1990 (1330)	WHO data and estimates[36]
HIV+ TB incidence/100,000 in 2005	385 – 2450	574 – 1330 (889)	
TB incidence/100,000 in 2023	358 – 841	411 – 598 (508)	
HIV+ TB incidence/100,000 in 2023	190 – 449	190 – 315 (245)	
TB mortality/100,000 in 2005	379 – 934	417 – 893 (628)	
HIV+ TB mortality/100,000 in 2005	275 – 814	348 – 801 (548)	
TB mortality/100,000 in 2023	65 – 187	100 – 187 (155)	
HIV+ TB mortality/100,000 in 2023	19 – 139	65.5 – 136 (102)	
TB notifications/100,000 in 2018	448 – 647	449 – 615 (511)	
TB notifications/100,000 in 2023	379 – 464	381 – 464 (445)	
Ratio of TB notifications to incidence in 2023	0.54 – 1.30	0.73 – 1.1 (0.88)	
Proportion of people starting TB treatment in 2023 who are HIV+	0.45 – 0.61	0.47 – 0.61 (0.58)	
Proportion of HIV+ people starting TB treatment in 2023 who are on ART	0.80 – 0.95	0.8 – 0.89 (0.83)	
Proportion of TB mortality in HIV- people in 2022 that occurs in people on TB treatment	0.17 – 0.35	0.17 – 0.35 (0.25)	

Proportion of TB mortality in HIV+ people in 2022 that occurs in people on TB treatment	0.14 – 0.91	0.14 – 0.25 (0.15)	
**Data from 2018 South African TB prevalence survey**			
TB prevalence/100,000 in men 2018	835 – 1352	835 – 1350 (1018)	South Africa TB prevalence survey[3] (see Section 1.1.11.9 for details)
TB prevalence/100,000 in women 2018	494 – 855	495 – 842 (565)	
TB prevalence/100,000 in HIV+ people	996 – 2249	997 – 1918 (1213)	
TB prevalence/100,000 in HIV- people	565 – 1108	565 – 1007 (684.8)	
Prevalence of self-reported TB symptoms in HIV- people with TB	0.22 – 0.48	0.22 – 0.38 (0.27)	
Prevalence of self-reported TB symptoms in HIV+ people with TB	0.18 – 0.70	0.39 – 0.68 (0.55)	
Proportion of HIV+ people reporting TB symptoms	0.090 – 0.23	0.090 – 0.23 (0.14)	
**Clinic visiting**			
Mean number of clinic visits per adult in 2022	1.50 – 1.90	1.51 – 1.87 (1.68)	See Section 1.1.9.5
Prevalence of symptoms in clinic attendees in 2022	0 – 0.30	0.16 – 0.30 (0.24)	

Increased prevalence of TB in clinic attendees relative to the general population in 2022	0.66 – 4.63	1.81 – 4.63 (3.42)	
**Household contact study data**			
Prevalence of infection in children in control households	0.046 – 0.13	0.060 – 0.13 (0.10)	See Section 1.1.12
Prevalence of infection in child household contacts of people diagnosed with TB who are HIV-	0.19 – 0.31	0.19 – 0.31 (0.24)	
Relative prevalence of infection in child household contacts of people diagnosed with TB who are HIV+ART+ compared to HIV-	0.50 – 0.99	0.50 – 0.79 (0.61)	
Relative prevalence of infection in child household contacts of people diagnosed with TB who are HIV+ART- compared to HIV-	0.41 – 0.87	0.41 – 0.73 (0.52)	
Relative prevalence of infection in child household contacts of HIV+ART+ people diagnosed with symptom-screen negative TB	0.35 – 2.69	0.76 – 0.98 (0.92)	

compared to symptom-screen positive TB			

1.2 Costing methods

We conducted primary data collection from Feb 7, 2024 to Feb 15, 2024 at six public health facilities (district hospitals, primary care clinics), including a district hospital laboratory. We observed workflows and interviewed TB nurses, district TB coordinators/managers, hospital managers, and laboratory managers to map TB service processes for cost parameterisation. We developed process maps for TB-related services at each level of the health system to enable parameterization of cost models to reflect a range of potential diagnostic algorithms. We estimated costs for all direct and ancillary services which could be included in a diagnostic algorithm; services and their cost estimates are listed in **Error! Reference source not found.**.

For each service we identified all resources used to produce the service, including capital (building, equipment and furniture, vehicles, and training) and recurrent (staff, supplies, maintenance, utilities, fuel, food and other recurrent) inputs. Resource quantities were obtained from facility and government documentation and in-person interviews. In the case of services not currently in operation at-scale in South Africa, we identified the potential resources involved using in-person interviews and data from pilot projects. Commodity resource prices were estimated using the National Health Laboratory Services (NHLS) state price list [49]. Staff salaries were estimated using the average salary across all grades per cadre (1), and using public service pay scales for non-health care workers [50]. Prices for other resources, as well as overheads and other fixed costs, were sourced from published and grey literature. We assumed a relative standard error of 20% for costs collected as primary data.

We supplemented primary data collection with comprehensive searches of the literature to identify suitable unit cost estimates for TB-related services, with a prioritization on the study having taken place within the last 5 years and the study having taken place in South Africa. We adapted recent unit cost estimates for symptom screening and sputum collection [51], lab-based Xpert MTB/RIF Ultra [52], mobile chest X-ray units, ward-based outreach teams [53], TB treatment [54], and HIV treatment [55], to our setting. Overhead costs for all health facility-based services were adapted from a study in Georgia to reflect the South African setting [56].

All costs from secondary data were classified as tradeable, non-tradeable, and staff costs. Tradeable goods included things that can easily be traded in the international market, such as supplies. To adjust these we converted the price to USD using the exchange rate for the year of study, and then inflated to 2023 USD using the US Consumer Price Index [57] [58]. Non-tradeable goods cannot be traded in international markets and are generally consumed in the country where they are produced; these include for example buildings and utilities. Non-tradeable and staff costs were adjusted by first inflating the price to 2023 Rand using the South Africa Consumer Price Index [58], and then converting to 2023 USD using the average official exchange rate for the year 2023 [59]. Where the 'reference' prices used were not from South Africa (e.g. overhead costs), we

applied an adjustment factor to staff and non-tradeable costs representing the relative GDP per capita expressed in current international dollars, converted by purchasing power parities [60].

Costs were parameterized using a mechanistic cost function approach, which accounted for variations in unit costs at different scales of implementation. We disaggregated costs into fixed programme costs (including the costs of running Ward-Based Outreach Teams, trainings, and transport of lab samples), fixed facility- and lab-level costs (including building, utilities, management, and overhead costs), and variable costs (including supplies, staff time and other costs) [61]. Fixed costs assumed no variation with scale and were set according to the national number of health facilities, labs, and Ward-Based Outreach Teams. We assumed a total of 4206 public clinics, 71 public hospitals, and 162 district labs [62], and a total of 7800 Ward-Based Outreach Teams in South Africa [63]. We assumed 80% of each of these would participate in active case finding activities regardless of the number of people screened/tested. We assumed that costs of TB treatment and ART were fully variable, as we were unable to find recent estimates reported by cost ingredient. Lifetime costs of ART were estimated according to total life expectancy by age.[16]

In all scenarios, we assumed that symptom screening occurring at the health facility would take place in a private room, conducted by an enrolled nurse. Similarly, we assumed that TUTT eligibility screening was conducted by a nurse in a private space. We assumed mobile chest radiography results were interpreted by a radiographer, and assumed some community mobilization would occur in each location to recruit participants. We assumed WBOTs worked in areas within 5 kilometres of a health facility, and that all 'high prevalence' targeting would take place within these areas. For the community-level approaches, we assumed some additional training would be required for WBOTs, and assumed a small cost for cool bags where sputum samples were collected at the community level. We assumed that sputum samples for all people who could produce sputum would be sent to a district laboratory for testing with Xpert Ultra. We assumed sputum samples collected by WBOTs would not require any extra investment for delivery to the district lab, as they would likely be delivered to local health facilities by WBOTs on foot, and would then included in existing shipments from the health facility to the district lab. We assumed each mobile radiography unit would have a maximum annual throughput of 12,000 tests [64]. We assumed that the national number of mobile radiography units would scale with the proportion of the population that was targeted, in increments of five.

Table 6 Costs of TB-related services (2023 USD)

Service	Fixed equipment costs	Fixed staff costs	Fixed overhead costs	Fixed transport costs	Variable consumables costs	Variable other costs	Variable staff costs	Total variable costs	Total fixed costs	Fixed unit definition
Symptom Screening - Static Sites	2.15	60.43	253.30	-	0.00	-	0.45	0.46	315.88	per clinic per year (n = 4206)
LTFU - Phone Call - Static Sites	48.21	249.38	436.89	-	-	0.34	0.57	0.90	734.48	per clinic per year (n = 4206)
Sputum collection attempt - Static Sites	58.22	1,349.19	3,264.35	-	1.90	-	1.13	3.04	4,671.76	per clinic per year (n = 4206)
Doctor Visit - Static Site	3,652.69	737.54	1,411.40	-	0.07	-	9.24	9.31	5,801.63	per clinic per year (n = 4206)
Results Notification - SMS	-	-	-	-	-	0.37	-	0.37	-	per hospital per year (n = 71)
Chest X-ray - Static Sites	293.79	581.15	1,127.26	-	0.13	-	8.36	8.50	2,002.20	per hospital per year (n = 71)
GXP Sample transport - District Laboratory	-	-	-	2,993.39	0.26	-	-	0.26	2,993.39	per lab per year (n = 162)
Full haemogram	434.05	561.99	1,227.51	-	3.52	-	0.68	4.20	2,223.55	per lab per year (n = 162)
GXP - District Laboratory	25,788.30	-	2,284.49	-	8.31	-	10.37	18.68	28,072.80	per lab per year (n = 162)
Chest X-ray - Mobile	8,077.08	15,335.61	803.35	17,772.75	0.13	-	3.31	3.45	41,988.80	per unit per year (n = 162)
Fixed costs - WBDOT pair	-	-	242.90	-	-	-	-	-	242.90	per team per year (n = 7800)
Symptom screen - community	-	-	-	-	0.03	-	1.19	1.23	-	per team per year (n = 7800)
Sputum collection attempt - community	135.16	2,840.16	-	-	1.25	-	3.35	4.60	2,975.32	per team per year (n = 7800)
ART treatment (per patient per year)	-	-	-	-	-	-	-	241.77	-	
Outpatient treatment (DS-TB) - adults	-	-	-	-	-	-	-	250.49	-	
Symptom screen - community - travel	-	-	-	-	0.21	-	7.14	7.34	-	

Table 7 Costs of TB-related services (2023 ZAR)

Service	Fixed equipment costs	Fixed staff costs	Fixed overhead costs	Fixed transport costs	Variable consumables costs	Variable other costs	Variable staff costs	Total variable costs	Total fixed costs	Fixed unit definition
Symptom Screening - Static Sites	39.62	1,115.00	4,673.52	-	0.04	-	8.37	8.41	5,828.14	per clinic per year (n = 4206)
LTFU - Phone Call - Static Sites	889.44	4,601.11	8,060.75	-	-	6.21	10.46	16.67	13,551.30	per clinic per year (n = 4206)
Sputum collection attempt - Static Sites	1,074.15	24,892.88	60,228.06	-	35.08	-	20.92	56.00	86,195.09	per clinic per year (n = 4206)
Doctor Visit - Static Site	67,393.08	13,607.83	26,040.60	-	1.32	-	170.40	171.72	107,041.51	per clinic per year (n = 4206)
Results Notification - SMS	-	-	-	-	-	6.76	-	6.76	-	per hospital per year (n = 71)
Chest X-ray - Static Sites	5,420.57	10,722.32	20,798.25	-	2.43	-	154.32	156.74	36,941.14	per hospital per year (n = 71)
GXP Sample transport - District Laboratory	-	-	-	55,228.78	4.87	-	-	4.87	55,228.78	per lab per year (n = 162)
Full haemogram	8,008.39	10,368.77	22,647.82	-	64.92	-	12.48	77.40	41,024.98	per lab per year (n = 162)
GXP - District Laboratory	475,800.48	-	42,149.47	-	153.32	-	191.32	344.65	517,949.95	per lab per year (n = 162)
Chest X-ray - Mobile	149,024.17	282,945.71	14,822.04	327,911.61	2.43	-	61.14	63.57	774,703.54	per unit per year (n = 162)
Fixed costs - WBDOT pair	-	-	4,481.64	-	-	-	-	-	4,481.64	per team per year (n = 7800)
Symptom screen - community	-	-	-	-	0.63	-	21.98	22.61	-	per team per year (n = 7800)
Sputum collection attempt - community	2,493.75	52,401.71	-	-	23.11	-	61.81	84.92	54,895.46	per team per year (n = 7800)
ART treatment (per patient per year)	-	-	-	-	-	-	-	-	-	
Outpatient treatment (DS-TB) - adults	-	-	-	-	-	-	-	-	-	
Symptom screen - community - travel	-	-	-	-	3.79	-	131.67	135.46	-	

1.3 CEA Outcomes

We used disability-adjusted life years (DALYs) as our primary outcome measure; DALYs were estimated as the sum of years of life lost (YLLs) and years of life lived with disability (YLDs), and were parameterized based on time spent in different health states in the model. We estimated YLLs using the weighted mean life expectancy at age at death by HIV status.[16,19,20] We estimated YLDs using disability weights for each health state, sourced from the most recent Global Burden of Disease study,[21] with expert-informed mapping for states not explicitly defined (e.g., false-positive TB treatment or chronic respiratory symptoms). All disability weights are specified in Table 8. We assumed that TB-related symptoms would subside after the first two months of treatment on average. Combined disability weights used a multiplicative approach.[22] YLDs were parameterized following a beta distribution in the model. All outcomes were discounted at 5% in the base case.[18]

Table 8 Disability weights used in model parameterization

Intervention	Mean	SE	Source and Notes
Full health (no TB)	0.00		
Active TB & HIV	0.41	0.07	[65]
Active TB, no HIV	0.33	0.06	[65]
HIV, no active TB	0.27	0.05	[65]
HIV on ART, no active TB	0.08	0.02	[65]
Post-TB	0.03	0.00	[66]
TB treatment due to a false-positive TB diagnosis	0.05	0.01	[65] mapped to health state "Generic uncomplicated disease: worry and daily medication"
HIV on ART, active TB	0.33	0.06	[65]
Treated TB	0.14	0.01	[65] Assumed 2 months equivalent to health state "Active TB"; 4 months equivalent to health state "Generic uncomplicated disease: worry and daily medication".
Chronic respiratory disease	0.02	0.01	[65] mapped to health state "COPD and other chronic respiratory problems, mild"

2 Supplementary Results

2.1 Model calibration

2.1.1 Fit to data

We identified a total of 1041 full fitting points using history matching and model emulation. See **Error! Reference source not found.** for the range and median values across the 1041 points of each input parameter varied during calibration, and **Error! Reference source not found.** for the range and median values of each calibration target in the 1041 points.

2.1.2 TB mortality rates in people with chronic conditions

The median rate of TB mortality for people with chronic conditions that cause TB symptoms compared to people without chronic conditions that cause TB symptoms in 2024 in the fitting parameter sets was 2.71 (95% range 2.17 – 3.43) in HIV- people, 1.82 (1.48 – 2.23) in HIV+ people who are not ART, and 2.29 (1.82 – 2.88) in HIV+ people who are on ART. These ratios are in line with the plausible ratio we elicited of 1.7 – 3.0 (see Section 1.1.7.2).

2.2 Additional results figures

Figure 5 Number of Xperts/testing coverage as part of active case finding in 2026, by HIV/ART status and scenario

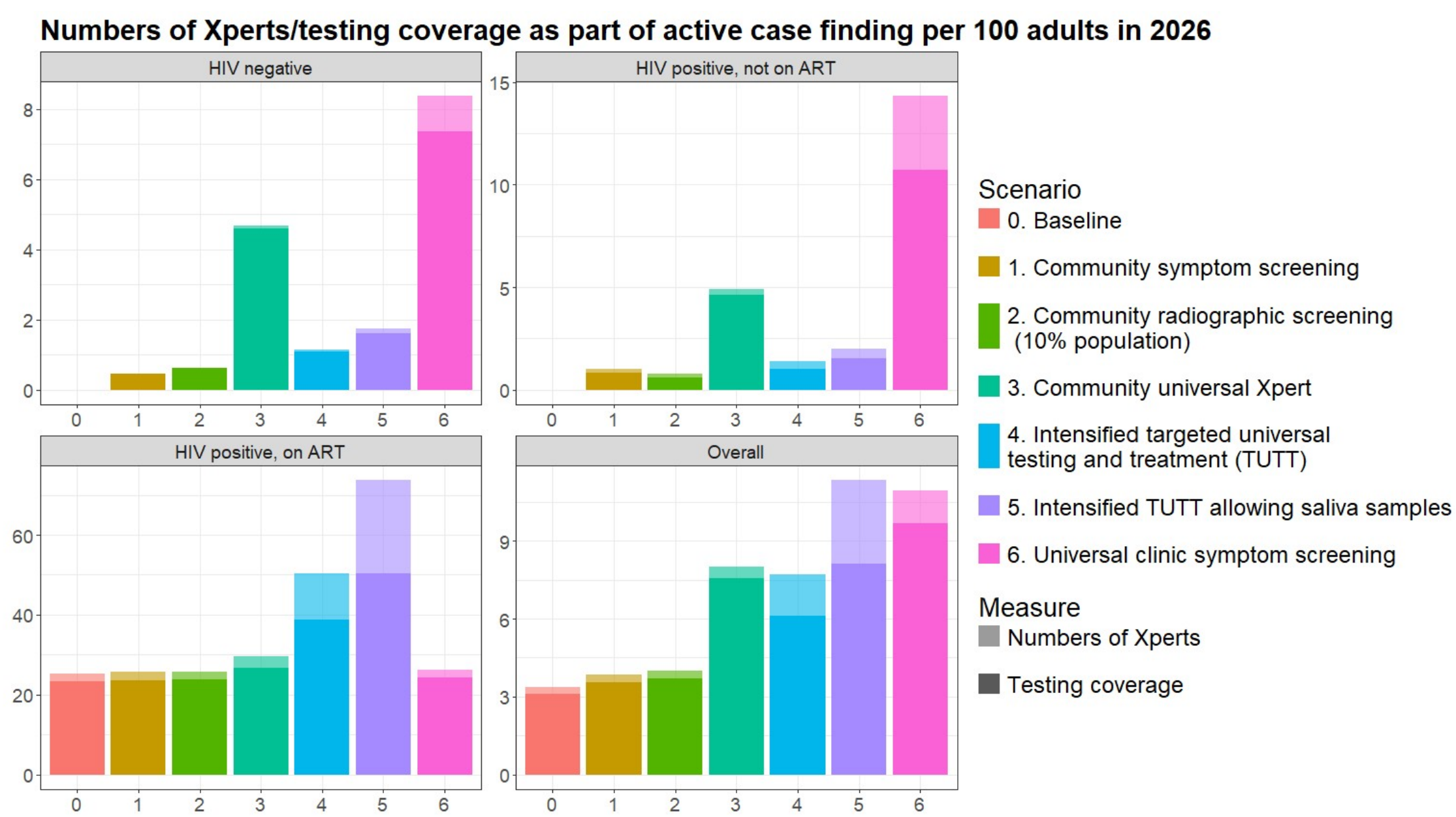

The full height of the bars shows the number of Xperts conducted. The height of the darker bars shows the proportion of the population who received a test. The difference in height between the two bars indicates that

some individuals received more than one Xpert as part of active case finding in 2026. The height of the bars are zero for HIV negative people and HIV positive people who are not on ART in the baseline scenario, as we assume that active case finding is only being conducted for people on ART in the baseline scenario

Figure 6 Notification rate of true positive TB in 2026, by HIV/ART status and scenario

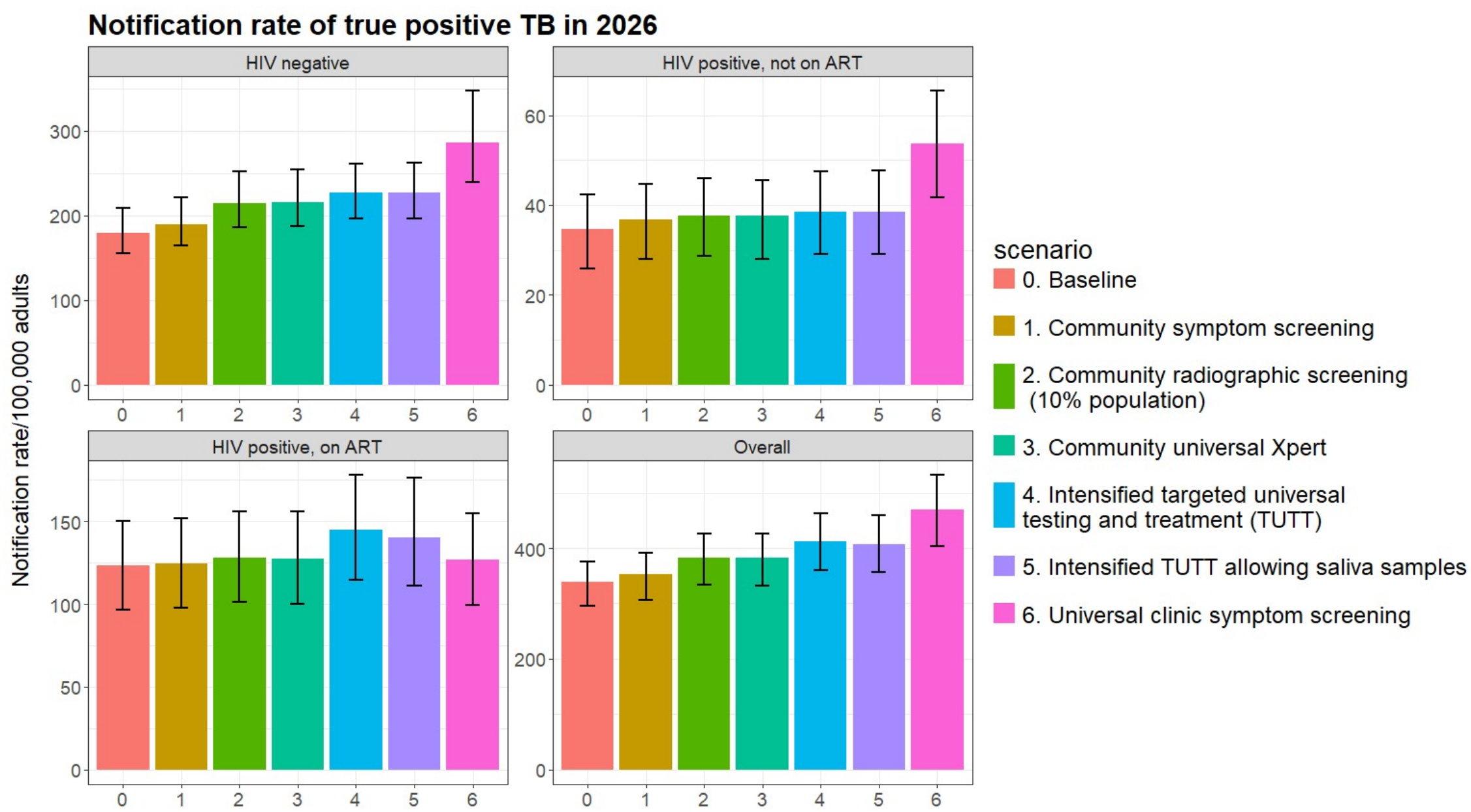

Figure 7 Mean TB episode duration in 2026, by HIV/ART status and scenario

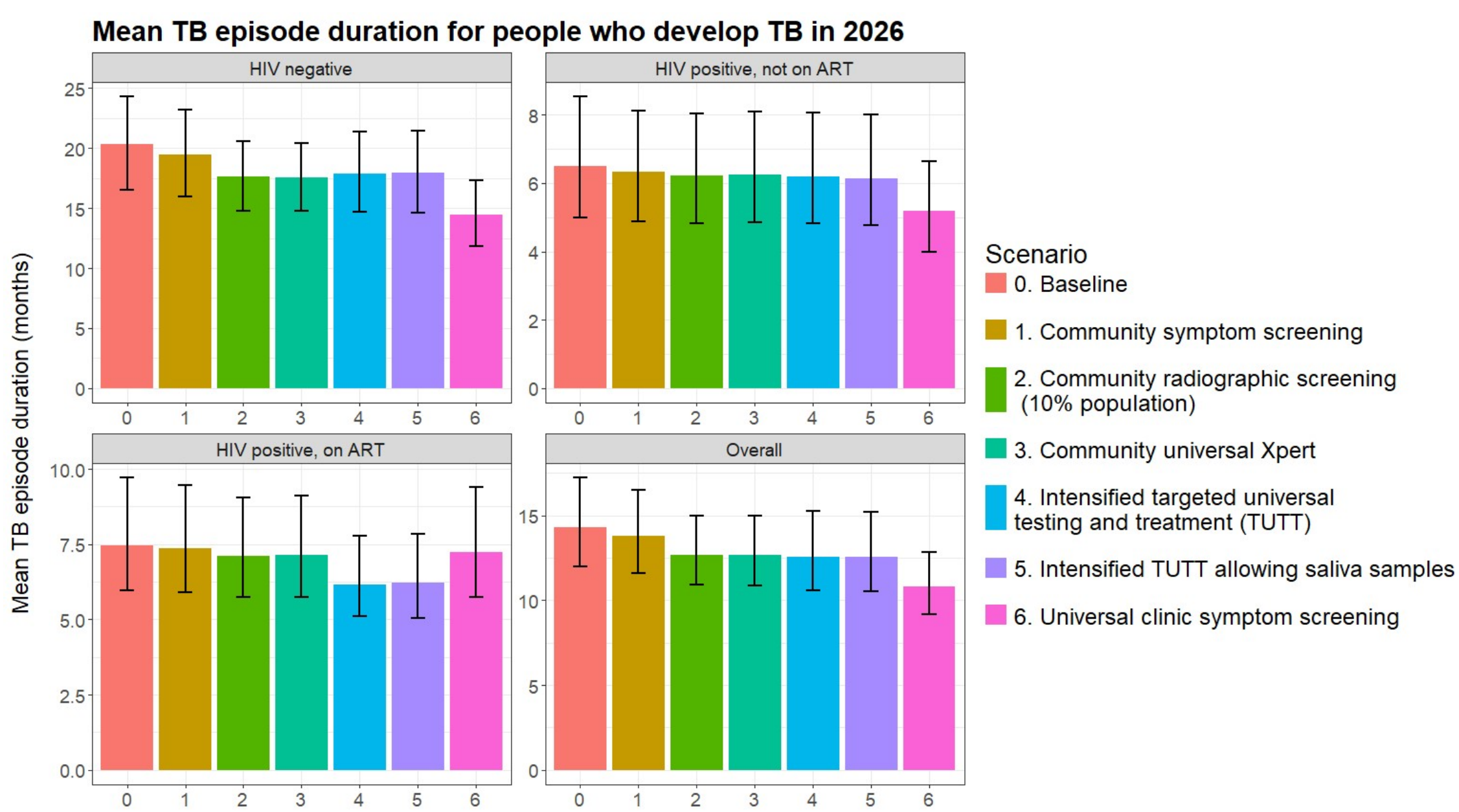

Figure 8 Yield of true positive TB per Xpert test conducted as part of active case finding in 2026, by HIV/ART status and scenario.

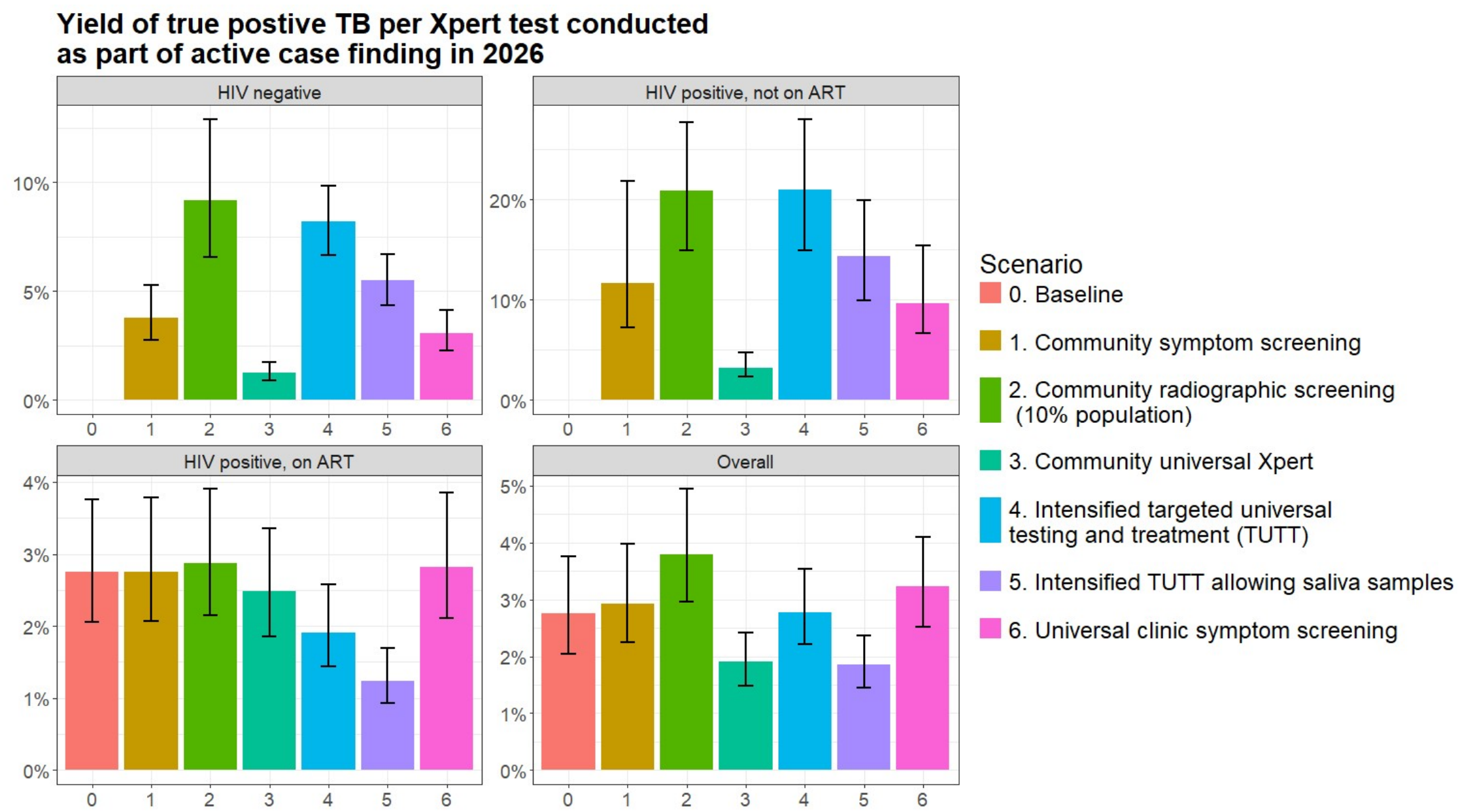

2.3 Partial rank correlation coefficients

Figure 9 Partial rank correlation coefficients plots, for each intervention approach

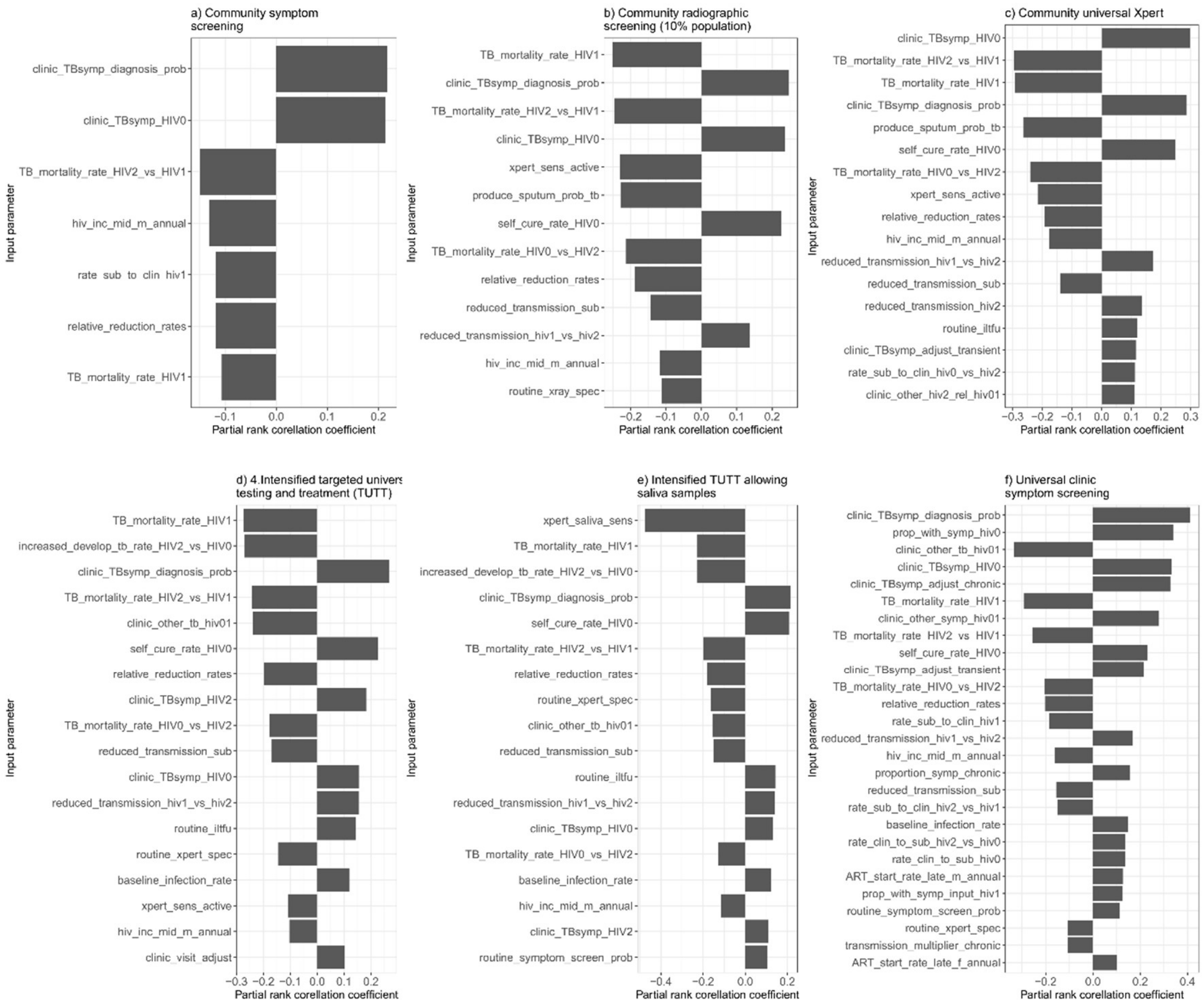

The plots show the strength of the relationship between the values of the input parameters that were varied in the mathematical model runs and the incremental cost effectiveness ratios. Input parameters where the absolute value of the point estimate of the partial rank correlation coefficient was less than 0.1 are not shown.

2.4 Additional cost-effectiveness results

Figure 10 Mean incremental costs and DALYs averted

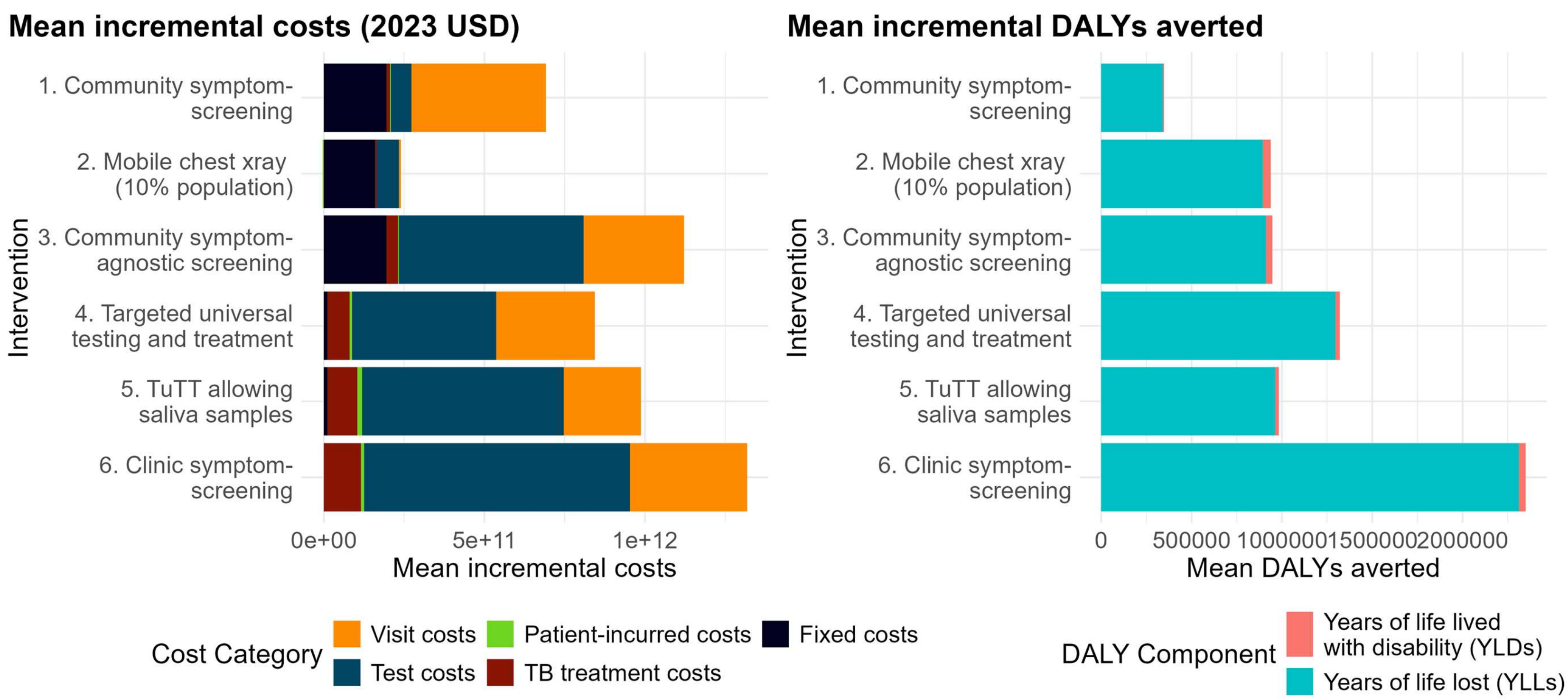

Table 9 Mean total and incremental costs and DALYs (South African Rand)

Variable	Baseline	1. Community symptom screening	2. Community radiographic screening	3. Community universal Xpert	4. Intensified TUTT	5. Intensified TUTT allowing saliva samples	6. Clinic symptom screening
Mean annual incidence of infectious TB in 2026-2035	438	423	391	389	376	385	328
Mean reduction in the incidence of infectious TB in 2026-3035, relative to baseline		3.4%	10.7%	11.2%	14.2%	12.1%	25.1%
Total cost (2023 ZAR)	R299.7B	R311.2B	R306.7B	R319.8B	R315.2B	R317.5B	R324.6B
Total provider- incurred cost	R267.7B	R279.2B	R274.8B	R287.8B	R283.2B	R285.4B	R292.4B
Total patient- incurred cost	R32.0B	R32.0B	R31.9B	R32.0B	R32.1B	R32.1B	R32.2B
Total DALYs	137.6M	137.3M	136.7M	136.7M	136.3M	136.3M	135.3M
Years of life lost	152.3M	151.9M	151.1M	151.1M	150.6M	150.7M	149.3M
Years of life lived with disability	19.1M	19.1M	19.1M	19.1M	19.1M	19.1M	19.1M
Mean incremental cost (2023 ZAR)		R11.5B	R7.1B	R20.1B	R15.5B	R17.8B	R24.9B
Mean incremental DALYs averted		0.3M	0.9M	0.9M	1.3M	1.3M	2.4M
ICER (2023 ZAR)		R35,055	R7,708	R21,851	R12,102	R14,375	R10,863

Table 10 Base intervention approaches proportion cost-effective by threshold

Approach	Mean ICER	95% Confidence Interval	Proportion of runs where the approach is cost-effective WTP = $618 / DALY averted	WTP = $985 / DALY averted	WTP = $3,1279 / DALY averted
1. Community symptom screening	$1,903	$1,134 - $3,389	0%	0%	96%
2. Community radiographic screening	$418	$287 - $613	98%	100%	100%
3. Community universal Xpert	$1,182	$829 - $1,779	0%	20%	100%
4. Intensified TUTT	$655	$428 - $994	45%	97%	100%
5. Intensified TUTT allowing saliva samples	$1,003	$602 - $1,601	3%	52%	100%
6. Clinic symptom screening	$586	$388 - $870	65%	99%	100%

Table 11 Full expansion path analysis results

Approach	Mean total costs	Mean total DALYs	Mean incremental costs	Mean incremental DALYs averted	Mean ICER
0. Baseline	16,243,176,136	137,628,110	-	-	-
1. Community symptom screening	16,865,500,413	137,279,860	622,324,278	348,250	1,899.98
2. Community radiographic screening	16,625,578,806	136,687,806	382,402,671	940,304	417.75
3. Community universal Xpert	17,333,952,359	136,680,056	1,090,776,223	948,054	1,184.33
4. Intensified targeted universal testing and treatment (TUTT)	17,084,446,546	136,304,837	841,270,410	1,323,273	655.92
5. Intensified TUTT allowing saliva samples	17,208,981,554	136,341,852	965,805,418	1,286,258	779.13
6. Universal clinic symptom screening	17,594,105,352	135,275,995	1,350,929,216	2,352,115	588.78
7. Community radiographic screening (20% population)	16,999,538,699	136,129,165	756,362,563	1,498,945	516.47
8. Community radiographic screening (40% population)	17,741,359,118	135,393,593	1,498,182,983	2,234,518	685.26

9. Community radiographic screening (60% population)	18,479,312,567	134,914,034	2,236,136,431	2,714,076	841.27
10. Community radiographic screening (80% population)	19,215,796,057	134,570,079	2,972,619,921	3,058,031	992.02
12. Universal clinic symptom screening, differentiated frequencies	17,724,267,665	135,113,269	1,481,091,529	2,514,841	603.39
13. Universal clinic symptom screening, every 2 months	18,017,252,489	134,994,481	1,774,076,353	2,633,629	690.55
14. TuTT, differentiated frequencies	17,257,252,308	135,891,947	1,014,076,172	1,736,163	603.58
15. TuTT, every 2 months	17,611,127,483	135,786,882	1,367,951,348	1,841,229	767.23
16. TuTT with saliva, differentiated frequency	17,742,644,015	135,820,366	1,499,467,879	1,807,744	858.77
17. TuTTs, every 2 months	18,146,794,667	135,665,769	1,903,618,531	1,962,341	1,003.64
18. Community universal Xpert + Universal clinic symptom screening	18,190,120,043	134,780,644	1,946,943,908	2,847,466	698.03
19. Community Community universal Xpert 10% + Intensified TUTT	17,374,145,422	135,530,219	1,130,969,287	2,097,891	551.20
20. Community universal Xpert 10% + Intensified TUTT with saliva	17,499,641,866	135,563,822	1,256,465,731	2,064,288	623.08
21. Community universal Xpert 20% + Universal clinic symptom screening	18,786,265,698	134,486,404	2,543,089,562	3,141,706	825.52
22. Community universal Xpert 20% + Intensified TUTT	17,981,989,953	135,081,714	1,738,813,817	2,546,396	697.43
23. Community universal Xpert 20% + Intensified TUTT with saliva	18,111,044,144	135,115,255	1,867,868,009	2,512,855	759.72
24. Community symptom screening 10% + Universal clinic symptom screening	17,722,386,944	135,104,766	1,479,210,809	2,523,344	600.32
25. Community symptom screening 10% + Intensified TUTT	16,902,699,649	136,012,940	659,523,514	1,615,170	419.06
26. Community symptom screening 10% + Intensified TUTT with saliva	17,025,146,881	136,053,562	781,970,745	1,574,548	511.13
27. Community symptom screening 20% + Universal clinic symptom screening	17,849,651,120	134,990,439	1,606,474,984	2,637,671	623.19
28. Community symptom screening 20% + Intensified TUTT	17,036,752,942	135,818,470	793,576,806	1,809,640	449.25
29. Community symptomscreening 20% + Intensified TUTT with saliva	17,159,835,355	135,863,776	916,659,219	1,764,334	533.76
30. Community radiographic screening 10% + Universal clinic symptom screening	17,967,494,698	134,779,728	1,724,318,562	2,848,382	617.87
31. Community radiographic screening 10% + Intensified TUTT	17,141,652,398	135,528,398	898,476,263	2,099,712	437.28

32. Community radiographic screening 10% + Intensified TUTT with saliva	17,265,204,395	135,565,462	1,022,028,259	2,062,648	506.90
33. Community radiographic screening 20% + Universal clinic symptom screening	18,336,557,786	134,478,926	2,093,381,651	3,149,184	677.48
34. Community radiographic screening 20% + Intensified TUTT	17,512,251,696	135,081,445	1,269,075,560	2,546,665	508.56
35. Community radiographic screening 20% + Intensified TUTT with saliva	17,636,930,366	135,119,888	1,393,754,230	2,508,222	567.25
36. Community radiographic screening 40% + Universal clinic symptom screening	19,072,624,152	134,074,340	2,829,448,016	3,553,770	810.67
37. Community radiographic screening 40% + Intensified TUTT	18,250,489,601	134,499,899	2,007,313,466	3,128,211	654.20
38. Community radiographic screening 60% + Universal clinic symptom screening	19,807,684,814	133,798,831	3,564,508,679	3,829,279	947.29
39. Community radiographic screening 60% + Intensified TUTT	18,987,178,879	134,124,956	2,744,002,744	3,503,154	798.05
40. Community radiographic screening 80% + Universal clinic symptom screening	20,542,012,364	133,601,116	4,298,836,229	4,026,994	1,086.23
41. Community radiographic screening 80% + Intensified TUTT	19,722,220,165	133,859,267	3,479,044,029	3,768,843	940.23
44. Intensified TUTT + Universal clinic symptom screening, every 2 months	18,639,206,884	134,497,055	2,396,030,748	3,131,055	782.18
45. Intensified TUTT + Universal clinic symptom screening, every 6 months	18,051,514,691	134,878,931	1,808,338,555	2,749,179	672.43

Figure 11 Cost-effectiveness acceptability curves for intervention approaches on the expansion pathway

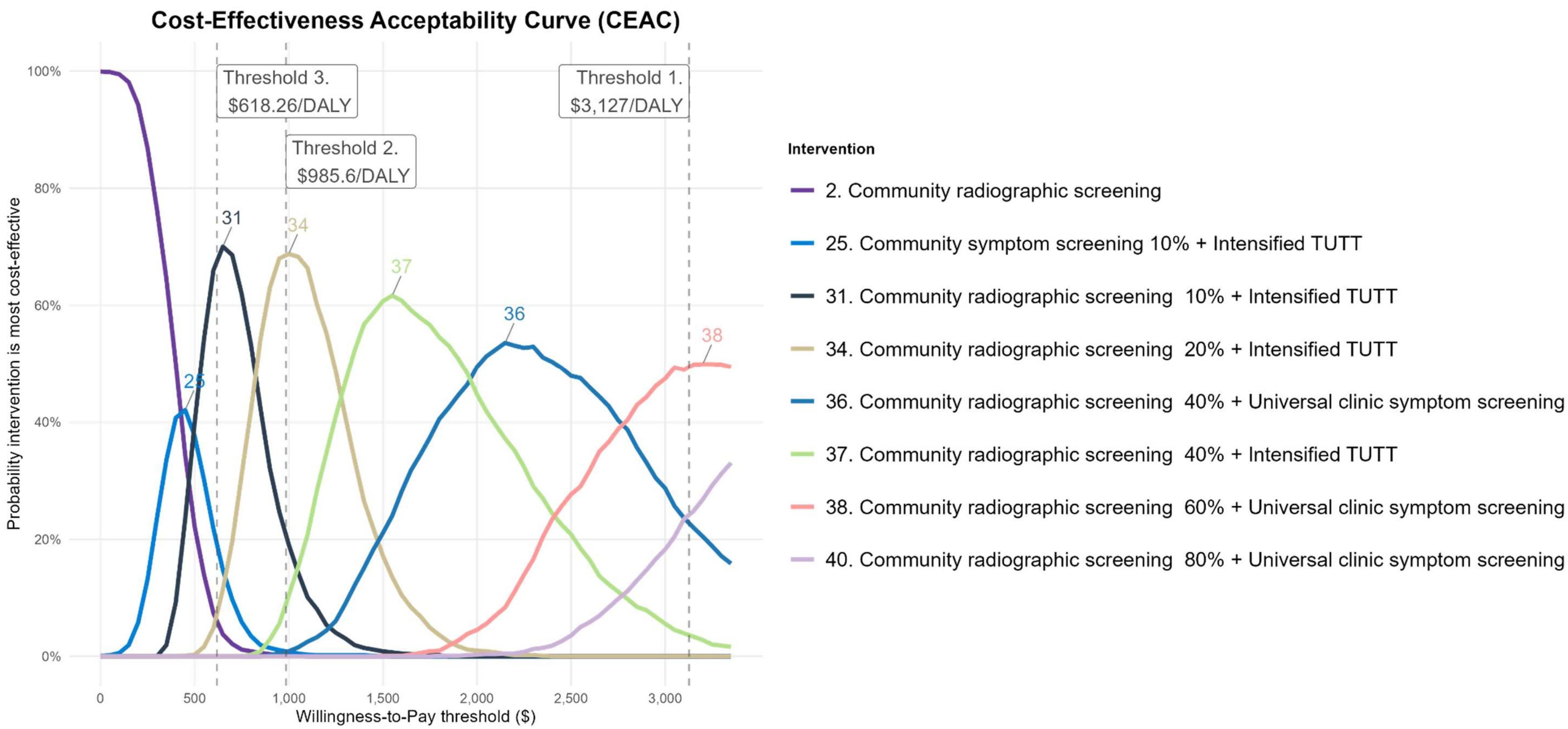

Figure 12 One-way scenario sensitivity analysis

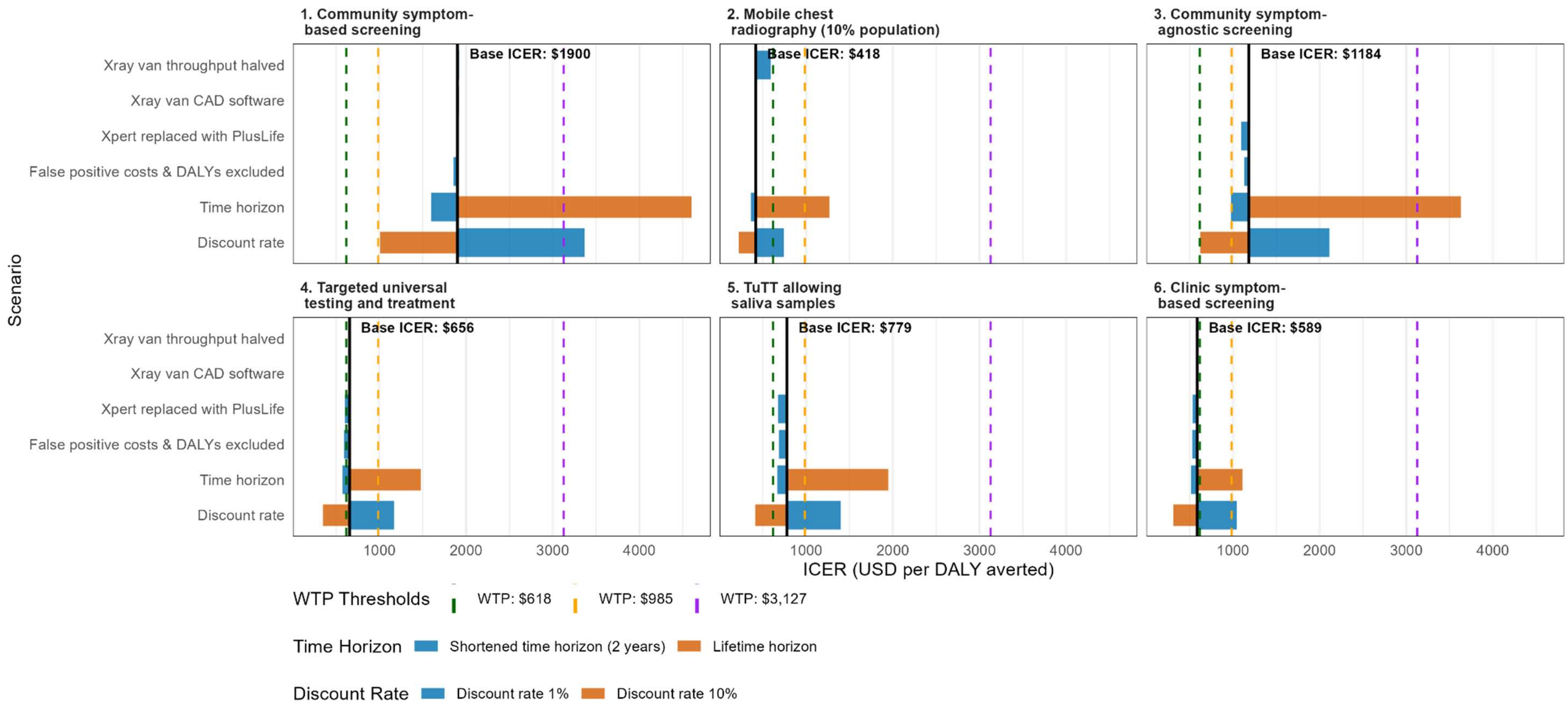

Figure 13 One-way sensitivity analysis expansion pathways

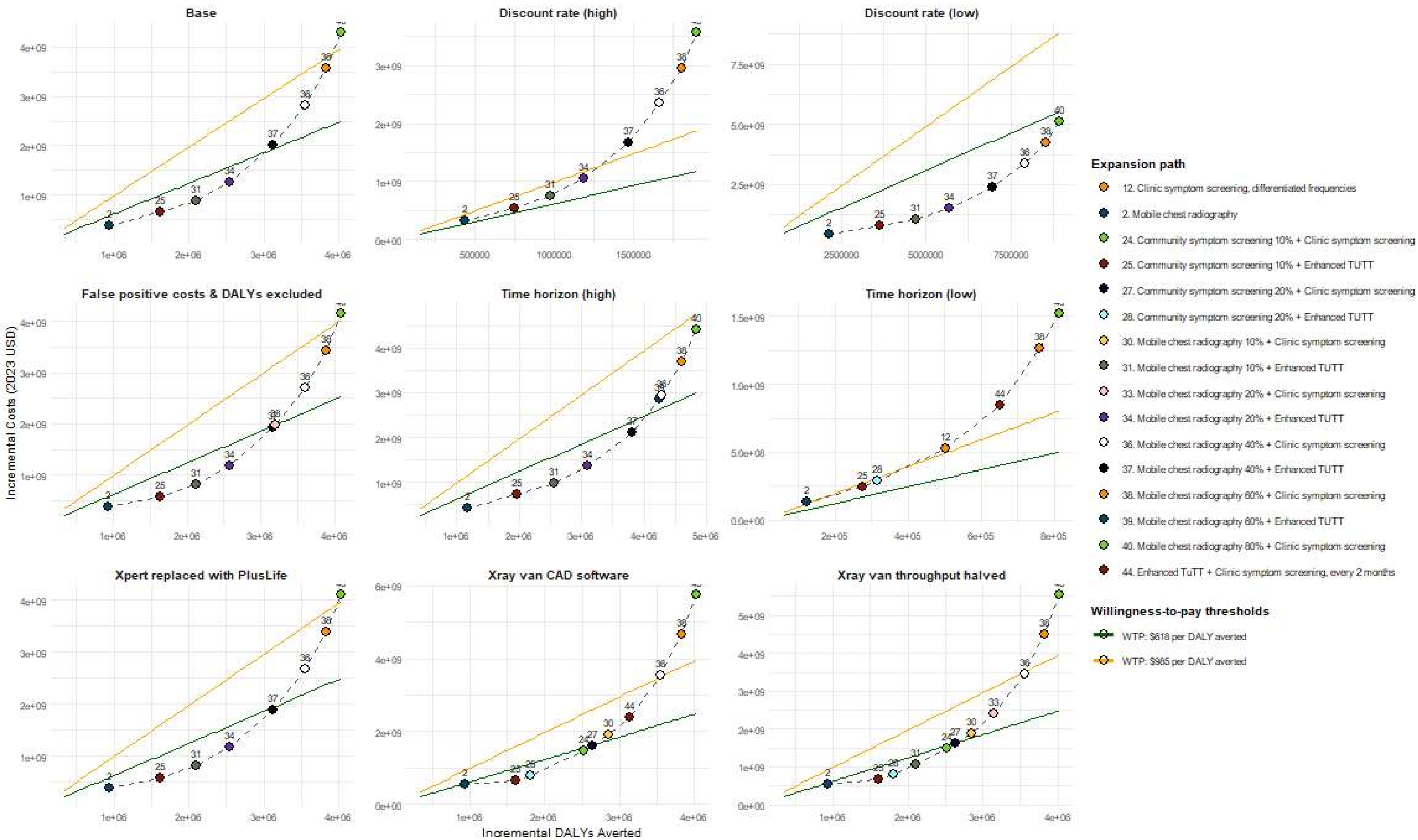

Figure 14 Community radiographic screening throughput analysis

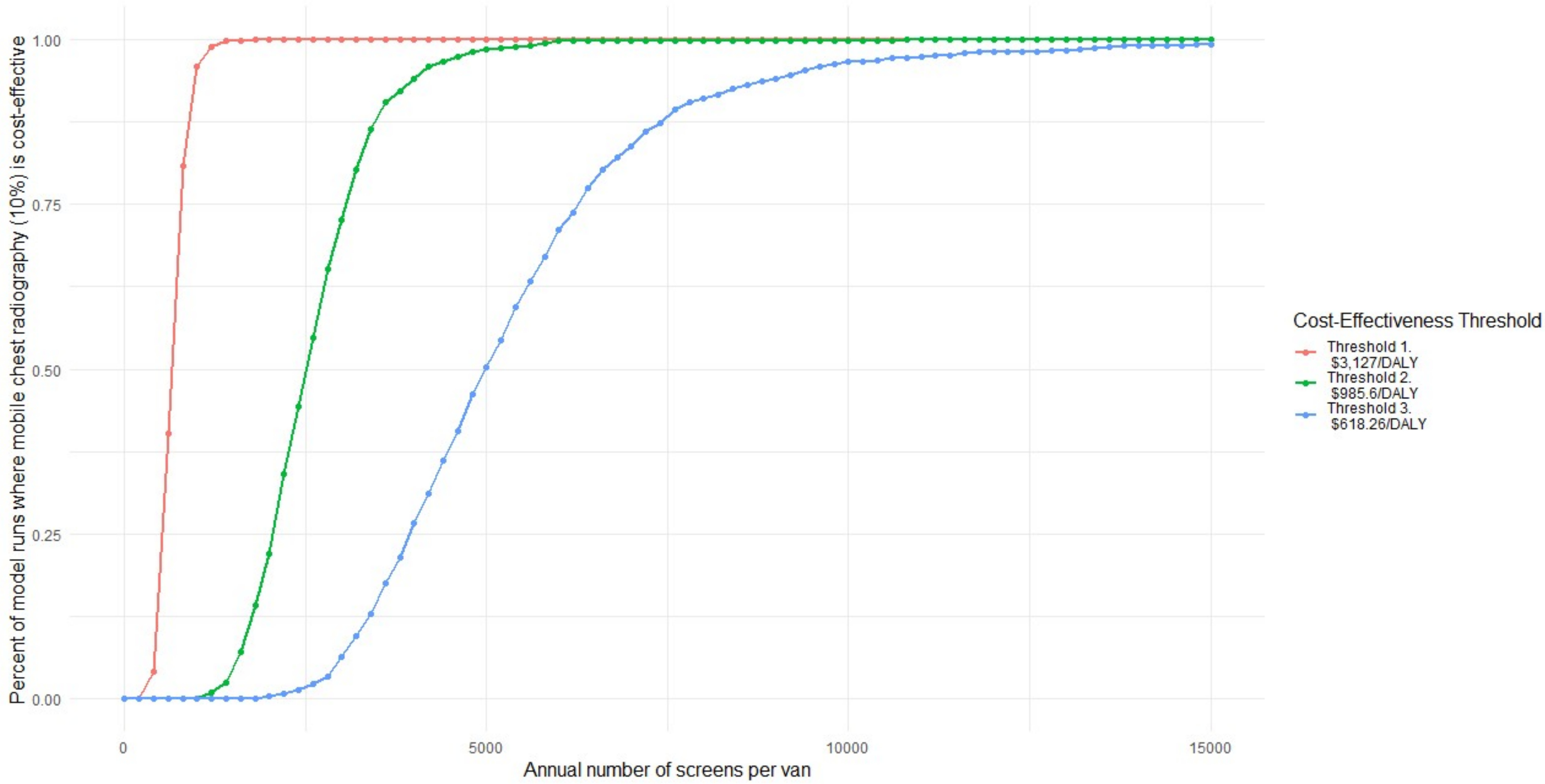

3 Additional discussion

We assumed that chest X-ray and Xpert have the same sensitivity in people with subclinical and clinical TB (with Xpert sensitivity higher in people who are passively seeking care in clinics only), and that the ability to produce sputum does not vary with reported symptoms. In practice, sensitivity and the ability to produce sputum are likely to be higher in people with clinical TB. The magnitude of the differences may be small however, due to the lack of clear distinction between people who do or do not report symptoms on a symptom screen[67]. In contrast, there may be more substantial differences in chest X-ray and Xpert sensitivity between people with less and more advanced TB, however it is unclear how such states should be defined or parameterised in models. In addition, the sensitivity and specificity of individual tests are likely to vary when they are used following a screening test[68], something not included in the model due to the lack of data to inform parameterisation. Finally, we used a wide plausible range for the specificity of Xpert Ultra, informed by the estimates of two systematic reviews[27,28], which may underestimate the true specificity of Xpert in community settings[69,70]. Our findings are likely to be robust to this limitation however, as our sensitivity analysis shows that excluding costs and DALYS associated with false positive diagnoses has little impact on the estimated ICERs for any approach (**Error! Reference source not found.**).

The model was calibrated to the prevalence of TB as estimated by the South Africa TB prevalence survey. As the survey only tested people who were screen-positive (on symptom screen and/or chest X-ray), and as a single Xpert and single culture have imperfect sensitivity for bacteriologically positive TB, we are likely to be underestimating the true prevalence of TB in South Africa in the model, and the true cost-effectiveness of the approaches.

The model was also calibrated to WHO estimates of TB incidence and mortality. The 2023 WHO TB report clarified that, in countries that have conducted national TB prevalence surveys, TB incidence estimates are based on "*on an assumed natural history in which most people with asymptomatic TB ultimately develop symptoms*". Incidence estimates for 2022 and 2023 are approximately equal to the sum of notifications and estimated TB deaths – suggesting that the assumed natural history may assume not only that most people who develop TB develop symptoms, but that they go on to either receive treatment or die of TB. We know that in practice TB can resolve without treatment however ('self-cure'), and allow self-cure to occur in the model. This means that, in theory, we could be underestimating TB incidence in the model. In practice, the confidence intervals around WHO incidence estimates are wide, and no fitting points were found near the upper limits of the ranges,

and so it is unlikely that increasing the upper limits of the ranges would have had any effect on the results.

We did not model self-clearance of infection, assuming instead that, once infected, people retain a small lifelong risk of reactivation. This means that we may be underestimating the impact and cost-effectiveness of the approaches[71]. The magnitude of this effect will depend on the rate at which risk of reactivation decreases with time since infection, and will be higher for approaches that result in greater reductions in TB incidence. The proportion of incident TB in the model that results from an infection occurring over 10 years ago was 8.0% (95% CI 6.8 – 9.7%) in 2025, 8.4% (95% CI 6.9 – 11.3%) in 2035 in the baseline scenario, and 16.0% (95% CI 12.5 – 22.8%) in 2035 in the clinic symptom screening scenario.

Screening coverage by sex was explicitly accounted for in the model in the clinic-based approaches, as rates of clinic visiting were parameterised by sex. In the community approaches, we assumed that the coverage of screening would be the same in men as for women. As men have a higher prevalence of TB than women in South Africa[3], and coverage of screening tends to be higher in women in practice[8], we may have overestimated the impact of the community screening approaches. This is particularly the case for the universal Xpert and symptom-based community screening approaches, which we assumed would be delivered door-to-door. The community radiographic screening approach would rely on people visiting a screening van, and could therefore be targeted at locations that men tend to visit.

Community case finding is likely to be targeted at areas of the country where it is thought that the prevalence of TB is above that of the country as a whole. We parameterised the relationship between intervention coverage and prevalence in people screened using data on TB notification rates by district in South Africa. Prevalence does not correspond perfectly with notification rates however, potentially leading us to overestimate how successful an approach would be at targeting the highest prevalence areas. On the other hand, targeting could be more successful than we simulate, as it is likely that it would be done at a finer resolution than district level, and force of infection may be higher from people in high prevalence areas than low prevalence areas (e.g. due to more crowded housing). We may therefore be under- or over-estimating how the impact and cost-effectiveness of the community approaches scales with their level of coverage.

We assumed in the community radiographic screening approach that people who were able to produce sputum would only be treated if they tested positive on Xpert Ultra. If some empirical treatment occurred of people with negative Xpert results by signs of TB on chest X-ray, then we may be underestimating the impact of community radiographic screening.

We assumed that, in routine implementation of active case finding, 44.1 – 50.4% of people without TB and 48.4 – 79.9% of people with TB would be able to produce a sputum sample for testing. These are pragmatic estimates, and many studies have achieved higher proportions (e.g. the Vukuzazi community health survey[8]). If a higher proportion of people with TB produced a sputum sample in practice, then the reductions in incidence and DALYs averted would be higher.

4 CHEERS 2022 Checklist

	Item	Guidance for Reporting	Reported in section
**TITLE**			
Title	1	Identify the study as an economic evaluation and specify the interventions being compared.	Title
**ABSTRACT**			
Abstract	2	Provide a structured summary that highlights context, key methods, results and alternative analyses.	Abstract
**INTRODUCTION**			
Background and objectives	3	Give the context for the study, the study question and its practical relevance for decision making in policy or practice.	Introduction
**METHODS**			
Health economic analysis plan	4	Indicate whether a health economic analysis plan was developed and where available.	Methods, subheading: Cost-effectiveness and expansion paths
Study population	5	Describe characteristics of the study population (such as age range, demographics, socioeconomic, or clinical characteristics).	Methods, subheading: Mathematical model
Setting and location	6	Provide relevant contextual information that may influence findings.	Methods, subheading: Mathematical model
Comparators	7	Describe the interventions or strategies being compared and why chosen.	Method, subheading: Baseline and intervention scenarios
Perspective	8	State the perspective(s) adopted by the study and why chosen.	Methods, subheading: Cost-effectiveness and expansion paths
Time horizon	9	State the time horizon for the study and why appropriate.	Methods, subheading: Baseline and intervention scenarios
Discount rate	10	Report the discount rate(s) and reason chosen.	Methods, subheadings: Costing Approach; Outcomes
Selection of outcomes	11	Describe what outcomes were used as the measure(s) of benefit(s) and harm(s).	Methods, subheading: Outcomes
Measurement of outcomes	12	Describe how outcomes used to capture benefit(s) and harm(s) were measured.	Methods, subheading: Outcomes

Valuation of outcomes	13	Describe the population and methods used to measure and value outcomes.	Methods, subheading: Outcomes
Measurement and valuation of resources and costs	14	Describe how costs were valued.	Methods, subheading: Costing Approach
Currency, price date, and conversion	15	Report the dates of the estimated resource quantities and unit costs, plus the currency and year of conversion.	Methods, subheading: Costing Approach. Supplement, Section 1.2
Rationale and description of model	16	If modelling is used, describe in detail and why used. Report if the model is publicly available and where it can be accessed.	Methods, subheading: Mathematical model. Supplement, section 1.1
Analytics and assumptions	17	Describe any methods for analysing or statistically transforming data, any extrapolation methods, and approaches for validating any model used.	Supplement, Section 1.1.13.2.3
Characterizing heterogeneity	18	Describe any methods used for estimating how the results of the study vary for sub-groups.	Methods, subheading: Mathematical model.
Characterizing distributional effects	19	Describe how impacts are distributed across different individuals or adjustments made to reflect priority populations.	Not done
Characterizing uncertainty	20	Describe methods to characterize any sources of uncertainty in the analysis.	Methods, subheading: Sensitivity Analysis
Approach to engagement with patients and others affected by the study	21	Describe any approaches to engage patients or service recipients, the general public, communities, or stakeholders (e.g., clinicians or payers) in the design of the study.	Methods, subheading: Cost-effectiveness and expansion paths
**RESULTS**			
Study parameters	22	Report all analytic inputs (e.g., values, ranges, references) including uncertainty or distributional assumptions.	Supplement
Summary of main results	23	Report the mean values for the main categories of costs and outcomes of interest and summarise them in the most appropriate overall measure.	Results
Effect of uncertainty	24	Describe how uncertainty about analytic judgments, inputs, or projections affect findings. Report the effect of choice of discount rate and time horizon, if applicable.	Results
Effect of engagement with patients and others affected by the study	25	Report on any difference patient/service recipient, general public, community, or stakeholder involvement made to the approach or findings of the study	Methods, subheading: Cost-effectiveness and expansion paths
**DISCUSSION**			
Study findings, limitations, generalizability, and current knowledge	26	Report key findings, limitations, ethical or equity considerations not captured, and how these could impact patients, policy, or practice.	Discussion
**OTHER RELEVANT INFORMATION**			

Source of funding	27	Describe how the study was funded and any role of the funder in the identification, design, conduct, and reporting of the analysis	Methods, subheading: Role of the funding source
Conflicts of interest	28	Report authors conflicts of interest according to journal or International Committee of Medical Journal Editors requirements.	Author information Subheading: Conflict of interest statement

5 References

1. Newton SM, Brent AJ, Anderson S, Whittaker E, Kampmann B. Paediatric tuberculosis. *The Lancet infectious diseases* 2008; **8**(8): 498-510.
2. United Nations DoEaSA, Population Division. World Population Prospects 2022, 2022.
3. Moyo S, Ismail F, Van Der Walt M, et al. Prevalence of bacteriologically confirmed pulmonary tuberculosis in South Africa, 2017–19: a multistage, cluster-based, cross-sectional survey. *The Lancet Infectious Diseases* 2022; **22**(8): 1172-80.
4. National Department of Health South Africa. The First National TB Prevalence Survey - South Africa 2018, 2021.
5. World Health Organization. Global tuberculosis report 2024, 2024.
6. Coussens AK, Zaidi SM, Allwood BW, et al. Classification of early tuberculosis states to guide research for improved care and prevention: an international Delphi consensus exercise. *The Lancet Respiratory Medicine* 2024.
7. Stuck L, Klinkenberg E, Abdelgadir Ali N, et al. Prevalence of subclinical pulmonary tuberculosis in adults in community settings: an individual participant data meta-analysis. *The Lancet Infectious Diseases* 2024.
8. Wong EB, Olivier S, Gunda R, et al. Convergence of infectious and non-communicable disease epidemics in rural South Africa: a cross-sectional, population-based multimorbidity study. *The Lancet Global Health* 2021; **9**(7): e967-e76.
9. Moyo S, Ismail F, Mkhondo N, et al. Healthcare seeking patterns for TB symptoms: Findings from the first national TB prevalence survey of South Africa, 2017–2019. *PLOS ONE* 2023; **18**(3): e0282125.
10. Claassens MM, Jacobs E, Cyster E, et al. Tuberculosis cases missed in primary health care facilities: should we redefine case finding? *The International journal of tuberculosis and lung disease* 2013; **17**(5): 608-14.
11. Chihota VN, Ginindza S, McCarthy K, Grant AD, Churchyard G, Fielding K. Missed Opportunities for TB Investigation in Primary Care Clinics in South Africa: Experience from the XTEND Trial. *PloS one* 2015; **10**(9): e0138149.
12. Randera-Rees S, Safari WC, Gareta D, Herbst K, Baisley K, Grant AD. Can we find the missing men in clinics? Clinic attendance by sex and HIV status in rural South Africa. *Wellcome Open Research* 2021; **6**(169): 169.
13. Health Systems Trust. HST Indicator Tool. https://indicators.hst.org.za/ (accessed 15/2/23 2021).
14. Alaba OA, McIntyre D. What do we know about health service utilisation in South Africa? *Development Southern Africa* 2012; **29**(5): 704-24.
15. Govender I, Karat AS, Olivier S, et al. Prevalence of Mycobacterium tuberculosis in sputum and reported symptoms among clinic attendees compared to a community survey in rural South Africa. *Clinical Infectious Diseases* 2021.
16. UNAIDS. AIDSInfo. http://www.unaids.org/en/dataanalysis/datatools/aidsinfo (accessed 20/10/23.
17. Ray M, Logan R, Sterne JA, et al. The effect of combined antiretroviral therapy on the overall mortality of HIV-infected individuals. *Aids* 2010; **24**(1): 123-37.
18. Gupta A, Wood R, Kaplan R, Bekker L-G, Lawn SD. Tuberculosis Incidence Rates during 8 Years of Follow-Up of an Antiretroviral Treatment Cohort in South Africa: Comparison with Rates in the Community. *PLOS ONE* 2012; **7**(3): e34156.
19. Gatechompol S, Sophonphan J, Ubolyam S, et al. Incidence and factors associated with active tuberculosis among people living with HIV after long-term antiretroviral therapy in Thailand: a competing risk model. *BMC Infectious Diseases* 2022; **22**(1): 346.
20. Suthar AB, Granich RM, Kato M, Nsanzimana S, Montaner JS, Williams BG. Programmatic implications of acute and early HIV infection. *Journal of Infectious Diseases* 2015: jiv430.

21. UNAIDS. AIDSInfo. http://www.unaids.org/en/dataanalysis/datatools/aidsinfo (accessed 31/08/23.
22. Zuma K, Zungu N, Moyo S, et al. The Sixth South African National HIV, Behavioural and Health Survey, 2024.
23. Johnson LF, May MT, Dorrington RE, et al. Estimating the impact of antiretroviral treatment on adult mortality trends in South Africa: A mathematical modelling study. *PloS Med* 2017; **14**(12): e1002468.
24. Ragonnet R, Flegg JA, Brilleman SL, et al. Revisiting the Natural History of Pulmonary Tuberculosis: A Bayesian Estimation of Natural Recovery and Mortality Rates. *Clinical Infectious Diseases* 2020.
25. Richards AS, Sossen B, Emery JC, et al. The natural history of TB disease-a synthesis of data to quantify progression and regression across the spectrum. *medRxiv* 2021: 2021.09.13.21263499.
26. Mathebula U, Emerson C, Agizew T, et al. Improving sputum collection processes to increase tuberculosis case finding among HIV-positive persons in Botswana. *Public Health Action* 2020; **10**(1): 11-6.
27. Zifodya JS, Kreniske JS, Schiller I, et al. Xpert Ultra versus Xpert MTB/RIF for pulmonary tuberculosis and rifampicin resistance in adults with presumptive pulmonary tuberculosis. *Cochrane Database of Systematic Reviews* 2021; (2).
28. Shapiro AE, Ross JM, Yao M, et al. Xpert MTB/RIF and Xpert Ultra assays for screening for pulmonary tuberculosis and rifampicin resistance in adults, irrespective of signs or symptoms. *Cochrane Database of Systematic Reviews* 2021; (3).
29. Wang Y, He Y, Wang L, Zhang Y-A, Wang M-S. Diagnostic Yield of Nucleic Acid Amplification Tests in Oral Samples for Pulmonary Tuberculosis: A Systematic Review and Meta-analysis. *Open Forum Infectious Diseases* 2023; **10**(3): ofad082.
30. Byanyima P, Kaswabuli S, Musisi E, et al. Feasibility and Sensitivity of Saliva GeneXpert MTB/RIF Ultra for Tuberculosis Diagnosis in Adults in Uganda. *Microbiology Spectrum* 2022; **10**(5): e00860-22.
31. Shenai S, Amisano D, Ronacher K, et al. Exploring Alternative Biomaterials for Diagnosis of Pulmonary Tuberculosis in HIV-Negative Patients by Use of the GeneXpert MTB/RIF Assay. *Journal of clinical microbiology* 2020; **51**(12): 4161-6.
32. World Health Organization. WHO consolidated guidelines on tuberculosis. Module 2: screening – systematic screening for tuberculosis disease. Geneva, 2021.
33. Mwansa-Kambafwile JRM, Jewett S, Chasela C, Ismail N, Menezes C. Initial loss to follow up of tuberculosis patients in South Africa: perspectives of program managers. *BMC Public Health* 2020; **20**(1): 622.
34. Glynn JR, Murray J, Bester A, Nelson G, Shearer S, Sonnenberg P. High rates of recurrence in HIV-infected and HIV-uninfected patients with tuberculosis. *The Journal of infectious diseases* 2010; **201**(5): 704-11.
35. Verver S, Warren RM, Beyers N, et al. Rate of Reinfection Tuberculosis after Successful Treatment Is Higher than Rate of New Tuberculosis. *American Journal of Respiratory and Critical Care Medicine* 2005; **171**(12): 1430-5.
36. World Health Organization. Global tuberculosis report 2023, 2023.
37. Mzembe T. Prevalence of Mycobacterium tuberculosis infection among adolescents in rural KwaZulu-Natal, South Africa: London School of Hygiene & Tropical Medicine; 2020.
38. Khan PY, Govender I, McCreesh N, et al. Africa Health Research Institute (AHRI) Household Contact study: a study protocol, statistical analysis and modelling plan. *Wellcome Open Research* 2024; **9**: 622.
39. McCreesh N, White RG. An explanation for the low proportion of tuberculosis that results from transmission between household and known social contacts. *Scientific reports* 2018; **8**(1): 5382.

40. Smith JP, Cohen T, Dowdy D, Shrestha S, Gandhi NR, Hill AN. Quantifying Mycobacterium tuberculosis Transmission Dynamics Across Global Settings: A Systematic Analysis. *American Journal of Epidemiology* 2023; **192**(1): 133-45.

41. Martinson NA, Nonyane BAS, Genade LP, et al. Evaluating systematic targeted universal testing for tuberculosis in primary care clinics of South Africa: A cluster-randomized trial (The TUTT Trial). *PloS Med* 2023; **20**(5): e1004237.

42. Berhanu RH, Lebina L, Nonyane BA, et al. Yield of Facility-based Targeted Universal Testing for Tuberculosis With Xpert and Mycobacterial Culture in High-Risk Groups Attending Primary Care Facilities in South Africa. *Clinical Infectious Diseases* 2023; **76**(9): 1594-603.

43. Iskauskas A, Vernon I, Goldstein M, et al. Emulation and History Matching Using the hmer Package. *Journal of Statistical Software* 2024; **109**(10): 1 - 48.

44. Andrews JR, Noubary F, Walensky RP, Cerda R, Losina E, Horsburgh CR. Risk of progression to active tuberculosis following reinfection with Mycobacterium tuberculosis. *Clinical infectious diseases* 2012; **54**(6): 784-91.

45. Dowdy DW, Chaisson RE. The persistence of tuberculosis in the age of DOTS: reassessing the effect of case detection. *Bulletin of the World Health Organization* 2009; **87**(4): 296-304.

46. Kasaie P, Andrews JR, Kelton WD, Dowdy DW. Timing of Tuberculosis Transmission and the Impact of Household Contact Tracing: An Agent-Based Simulation Model. *American Journal of Respiratory and Critical Care Medicine* 2014; **189**(7): 845-52.

47. McCarthy K, Fielding K, Churchyard GJ, Grant AD. Empiric tuberculosis treatment in South African primary health care facilities - for whom, where, when and why: Implications for the development of tuberculosis diagnostic tests. *PLOS ONE* 2018; **13**(1): e0191608.

48. Foster N, Vassall A, Cleary S, Cunnama L, Churchyard G, Sinanovic E. The economic burden of TB diagnosis and treatment in South Africa. *Social Science & Medicine* 2015; **130**: 42-50.

49. National Health Laboratory Service. NHLS Price List 2023-2024. 2023; 2023.

50. Department Public Service and Administration South Africa. Hourly rates payable to contract employees (persons appointed on a sessional basis) in the identified occupations in the Public Health and Social Development Sectors, 2023.

51. Martinson NA, Nonyane BAS, Genade LP, et al. Evaluating systematic targeted universal testing for tuberculosis in primary care clinics of South Africa: A cluster-randomized trial (The TUTT Trial). *PLOS Medicine* 2023; **20**(5): e1004237.

52. Cassim N, Coetzee LM, Makuraj AL, Stevens WS, Glencross DK. Establishing the cost of Xpert MTB/RIF mobile testing in high-burden peri-mining communities in South Africa. *African Journal of Laboratory Medicine* 2021; **10**(1).

53. Daviaud E, Besada D, Budlender D, Sanders D, Kerber K. Saving lives, saving costs: investment case for community health workers in South Africa. Samrc; 2018. p. 49-63.

54. Siapka M, Vassall A, Cunnama L, et al. Cost of tuberculosis treatment in low- and middle-income countries: systematic review and meta-regression. *The International Journal of Tuberculosis and Lung Disease* 2020; **24**(8): 802-10.

55. Meyer-Rath G, van Rensburg C, Chiu C, Leuner R, Jamieson L, Cohen S. The per-patient costs of HIV services in South Africa: systematic review and application in the South African HIV investment case. *PloS one* 2019; **14**(2): e0210497.

56. Chikovani I, Shengelia N, Marjanishvili N, et al. Cost of TB services in the public and private sectors in Georgia (No 2). *The International Journal of Tuberculosis and Lung Disease* 2021; **25**(12): 1019-27.

57. Turner HC, Lauer JA, Tran BX, Teerawattananon Y, Jit M. Adjusting for Inflation and Currency Changes Within Health Economic Studies. *Value in Health* 2019; **22**(9): 1026-32.

58. The World Bank. Consumer price index. International Financial Statistics database; 2023.

59. The World Bank. Official exchange rate (LCU per US$, period average). International Financial Statistics database; 2023.

60. The World Bank. GDP per capita, PPP (current international $). 2023.

61. d'Elbee M, Terris-Prestholt F, Briggs A, et al. Estimating health care costs at scale in low- and middle-income countries: Mathematical notations and frameworks for the application of cost functions. *Health Econ* 2023; **32**(10): 2216-33.
62. National Institute For Communicable Diseases.
63. Schneider H, Sanders D, Besada D, Daviaud E, Rohde S. Ward-based primary health care outreach teams in South Africa: developments, challenges and future directions. South African Health Review 2018; 2018. p. 59-66.
64. Proaño A, Bravard MA, López JW, et al. Dynamics of Cough Frequency in Adults Undergoing Treatment for Pulmonary Tuberculosis. *Clinical Infectious Diseases* 2017; **64**(9): 1174-81.
65. Evaluation IfHMa. Global Burden of Disease Study Disability Weights. 2019.
66. Tomeny EM, Nightingale R, Chinoko B, et al. TB morbidity estimates overlook the contribution of post-TB disability: evidence from urban Malawi. *BMJ Glob Health* 2022; **7**(5).
67. McCreesh N, MacPherson P, Bampi JVB, Engel N, Kranzer K, Khan PY. Reported Tuberculosis Symptoms: An Inadequate Classifier of Disease State. *Clinical Infectious Diseases* 2025: ciaf611.
68. Kendall EA, Denkinger CM, Cattamanchi A, Dowdy DW, Andrews JR. Whom tuberculosis tests detect and why it matters: implications for diagnostic algorithms. *The Lancet Microbe* 2025; **6**(12).
69. McCreesh N, Govender I, Grant AD, Khan PY. Tuberculosis Prevalence in People Tested Is a Strong Predictor of Xpert Specificity in Community and Risk Group Screening. *The Journal of Infectious Diseases* 2025: jiaf418.
70. Veeken LD, Schwalb A, Horton KC, et al. Mind the clinic-community gap: how concerned should we be about false positive test results in mass tuberculosis screening? *The Journal of Infectious Diseases* 2025: jiaf268.
71. Ryckman TS, Shrestha S, Fojo AT, Kasaie P, Dowdy DW, Kendall EA. Modeling the impact of case finding for tuberculosis: The role of infection dynamics. *medRxiv* 2025: 2025.04.15.25325877.
