# Supplementary material for: Projected population level impact and cost-effectiveness of clinic and community-based tuberculosis screening approaches
Source: PLOS Glob Public Health. 2026 Sep 11;6(9):e0006738. doi: 10.1371/journal.pgph.0006738 (PMC13567835; doi:10.1371/journal.pgph.0006738)
Supplement: Supplementary file 2 [file pgph.0006738.s002.pdf]

Projected population level impact and cost-effectiveness of clinic and community-based tuberculosis screening approaches

S2 Appendix

Baseline and intervention approach flowcharts

Baseline

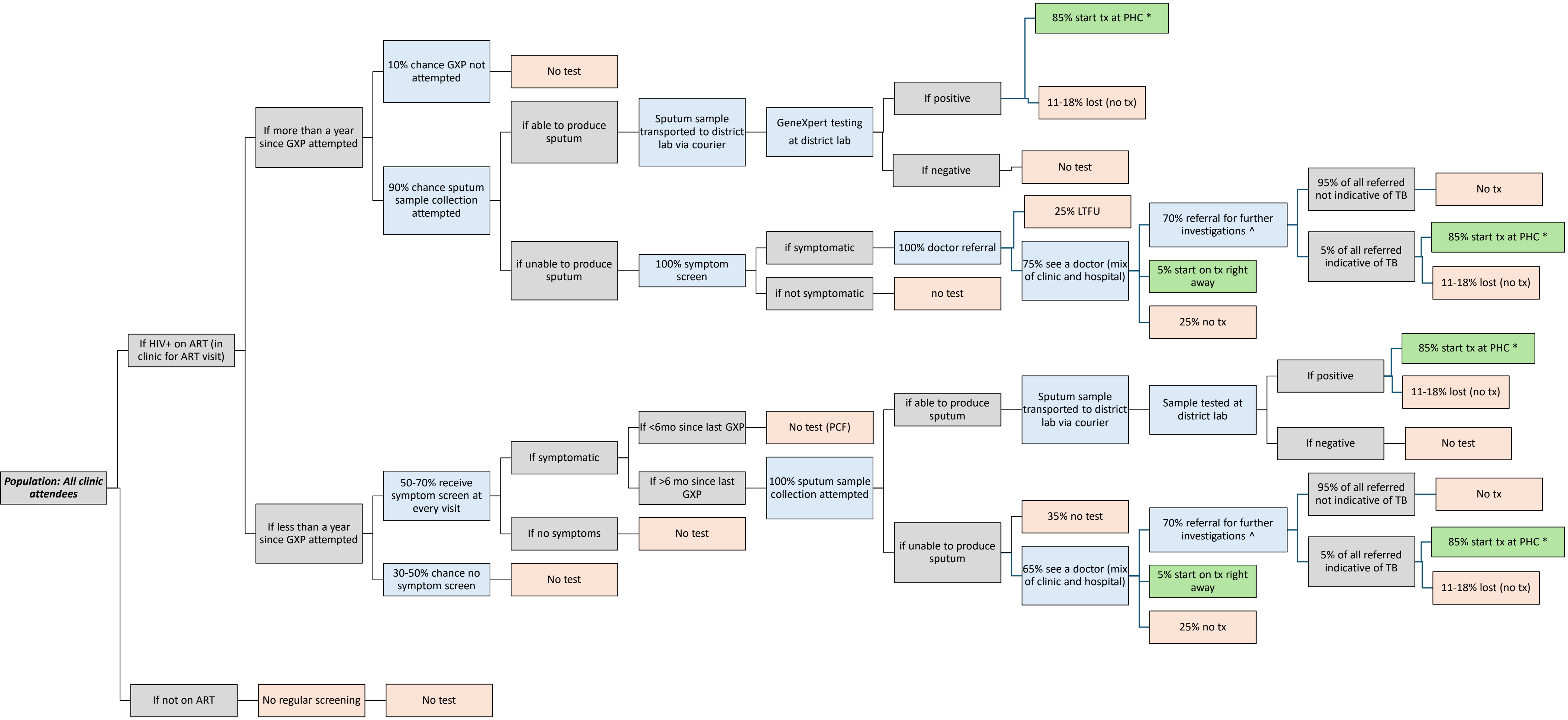

* Linkage to care activities: 50% of people testing positive called by nurse, 40% of people receiving GXP notified of result via automated text message by lab

^ Further investigations: 100% chest xray, 50% sputum attempt, 25% GeneXpert, 10% blood test FBC

1. Community symptom screening

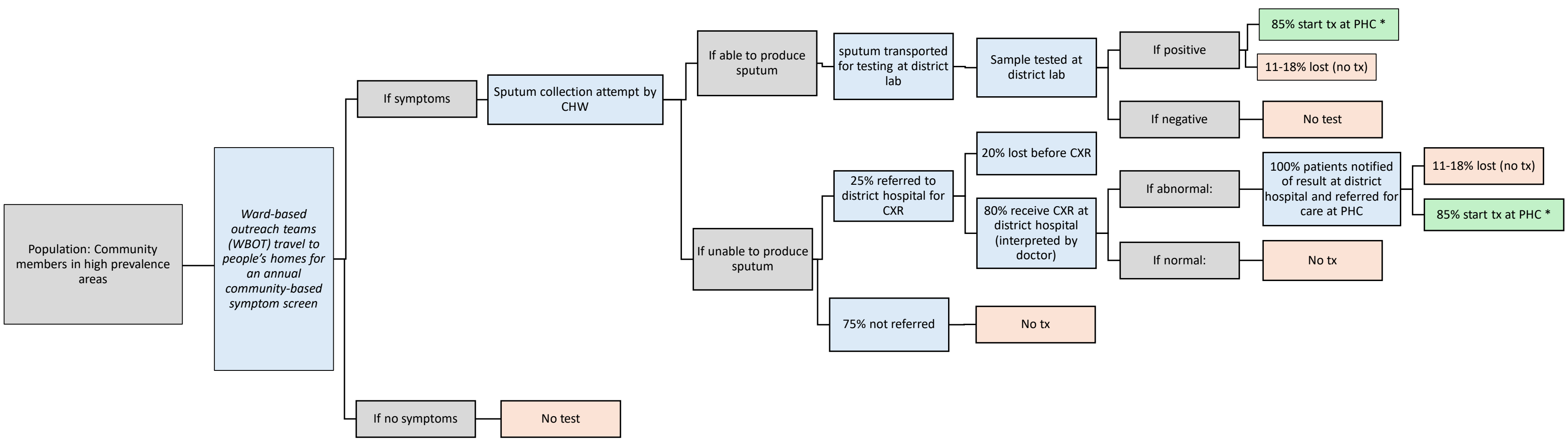

* Linkage to care activities: 50% of people testing positive called by nurse, 40% of people receiving GXP notified of result via automated text message by lab
^ Further investigations: 100% chest xray, 50% sputum attempt, 25% GeneXpert, 10% blood test FBC

2. Community radiographic screening

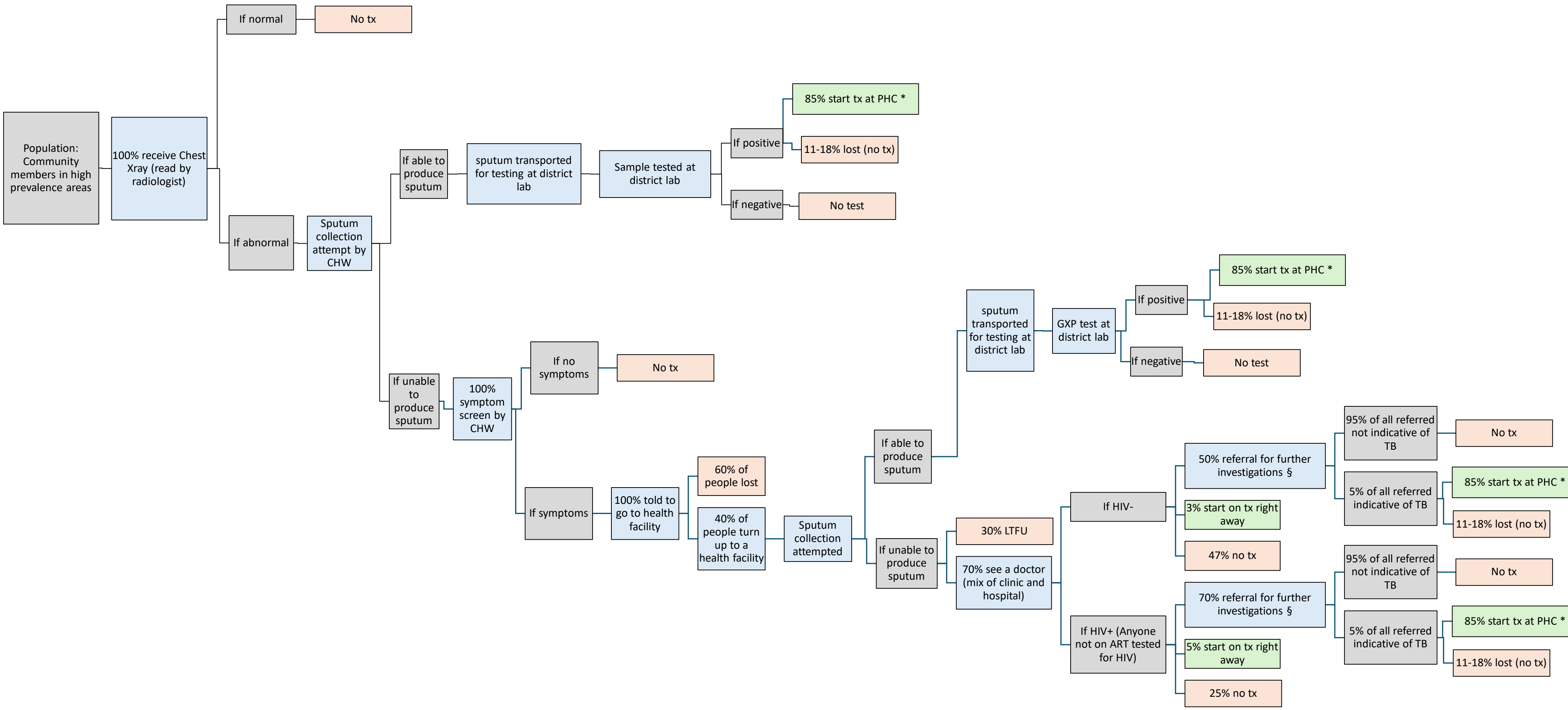

* Linkage to care activities: 50% of people testing positive called by nurse, 40% of people receiving GXP notified of result via automated text message by lab

§ Further investigations: 50% sputum attempt, 25% GeneXpert, 10% blood test FBC

3. Community universal Xpert

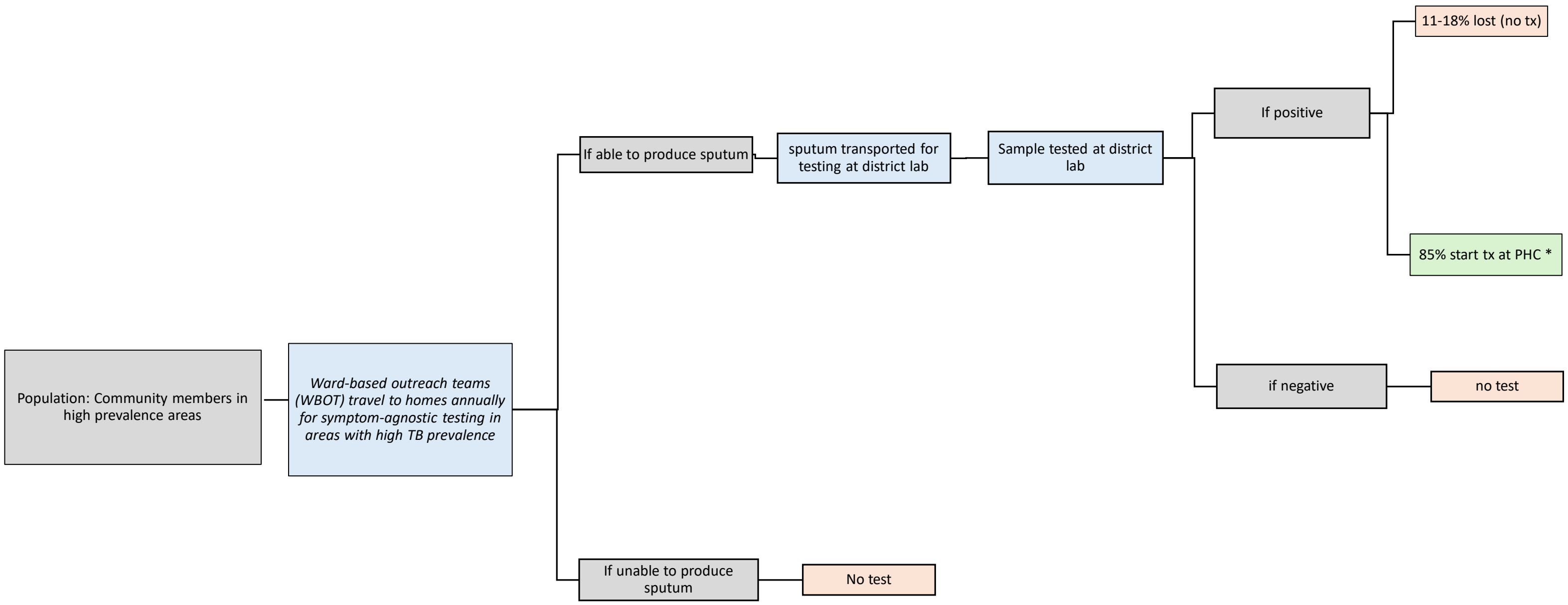

* Linkage to care activities: 50% of people testing positive called by nurse, 40% of people receiving GXP notified of result via automated text message by lab
^ Further investigations: 100% chest xray, 50% sputum attempt, 25% GeneXpert, 10% blood test FBC

4. Intensified targeted universal testing and treatment (TUTT)

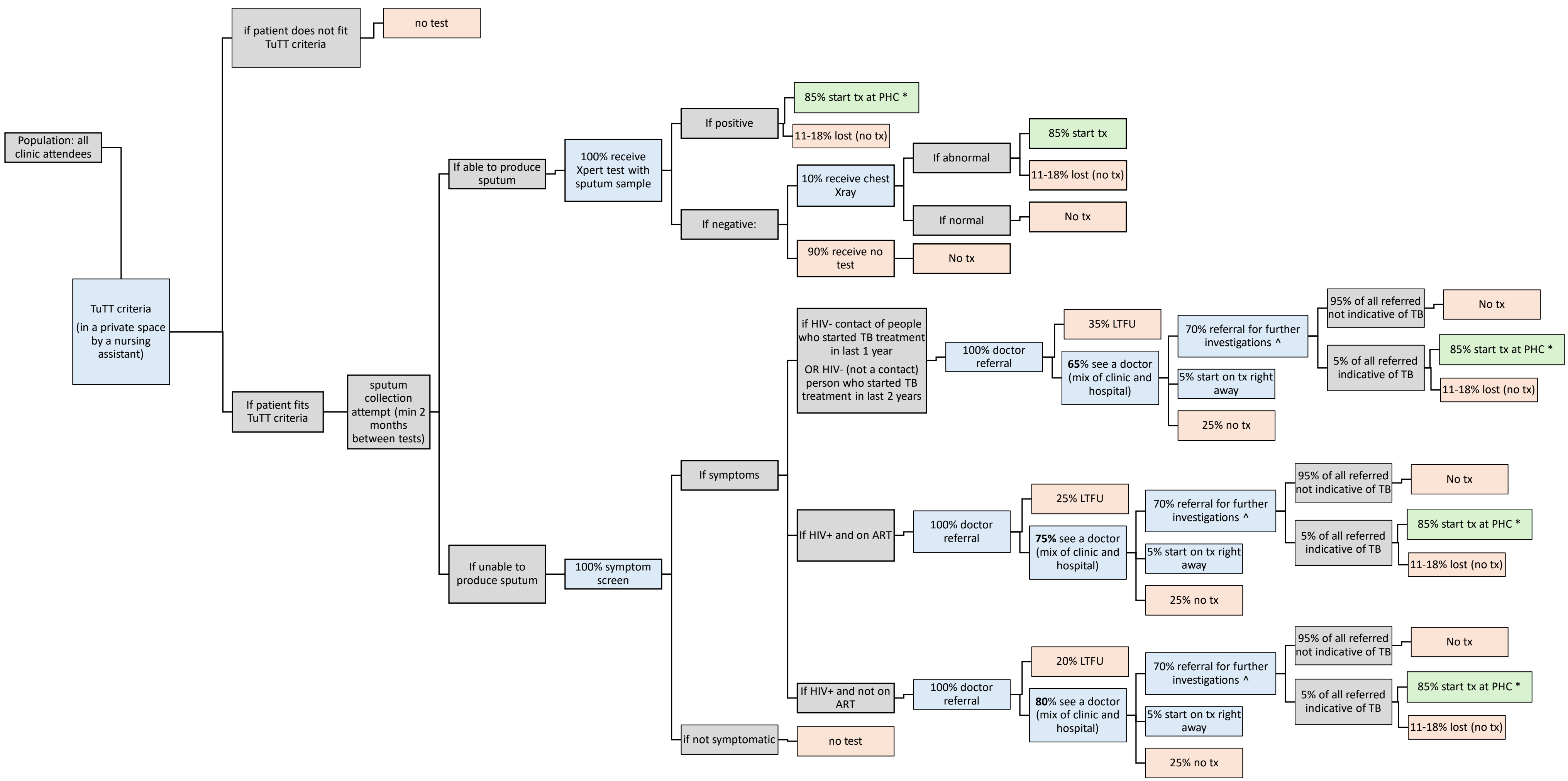

* Linkage to care activities: 50% of people testing positive called by nurse, 40% of people receiving GXP notified of result via automated text message by lab
^ Further investigations: 100% chest xray, 50% sputum attempt, 25% GeneXpert, 10% blood test FBC

5. Intensified TUTT allowing saliva samples

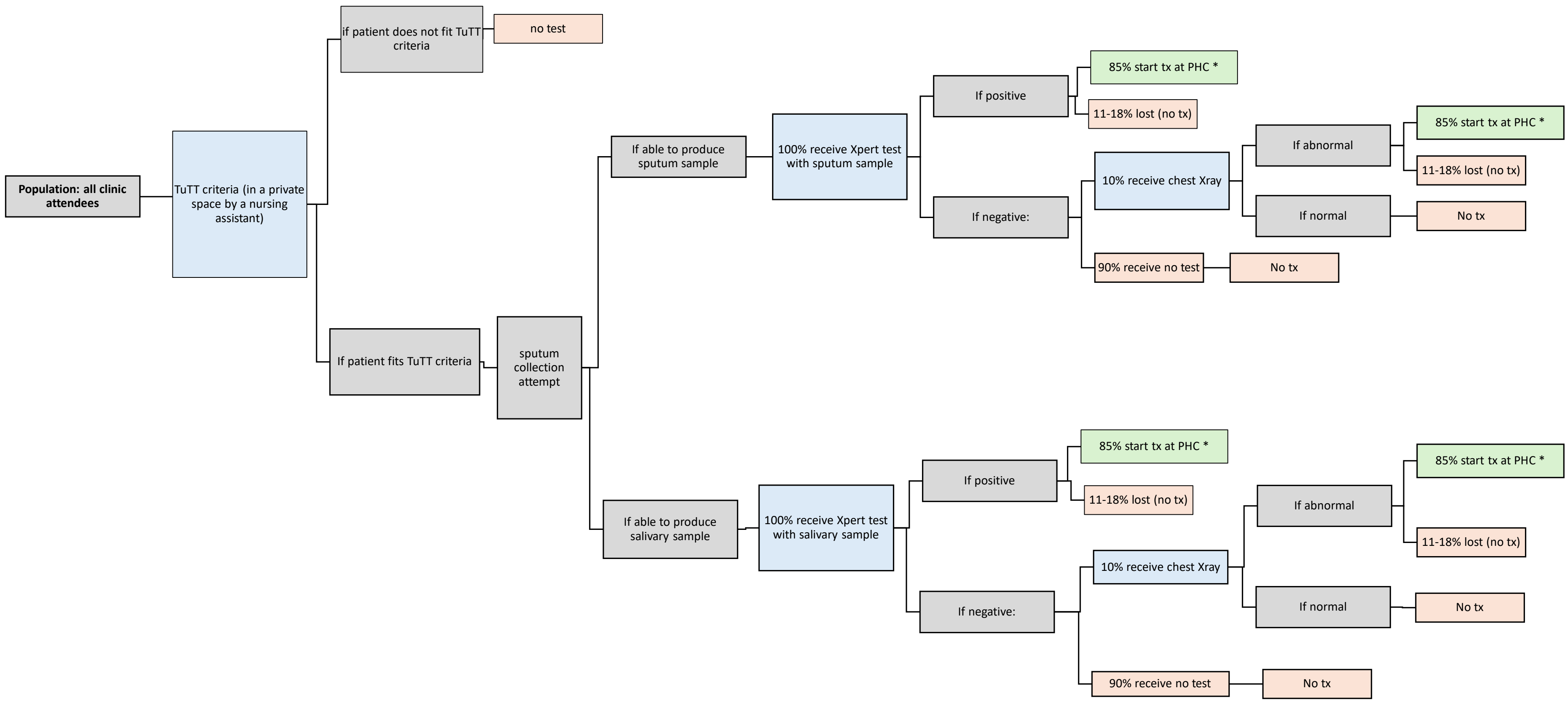

* Linkage to care activities: 50% of people testing positive called by nurse, 40% of people receiving GXP notified of result via automated text message by lab
^ Further investigations: 100% chest xray, 50% sputum attempt, 25% GeneXpert, 10% blood test FBC

6. Universal clinic symptom screening

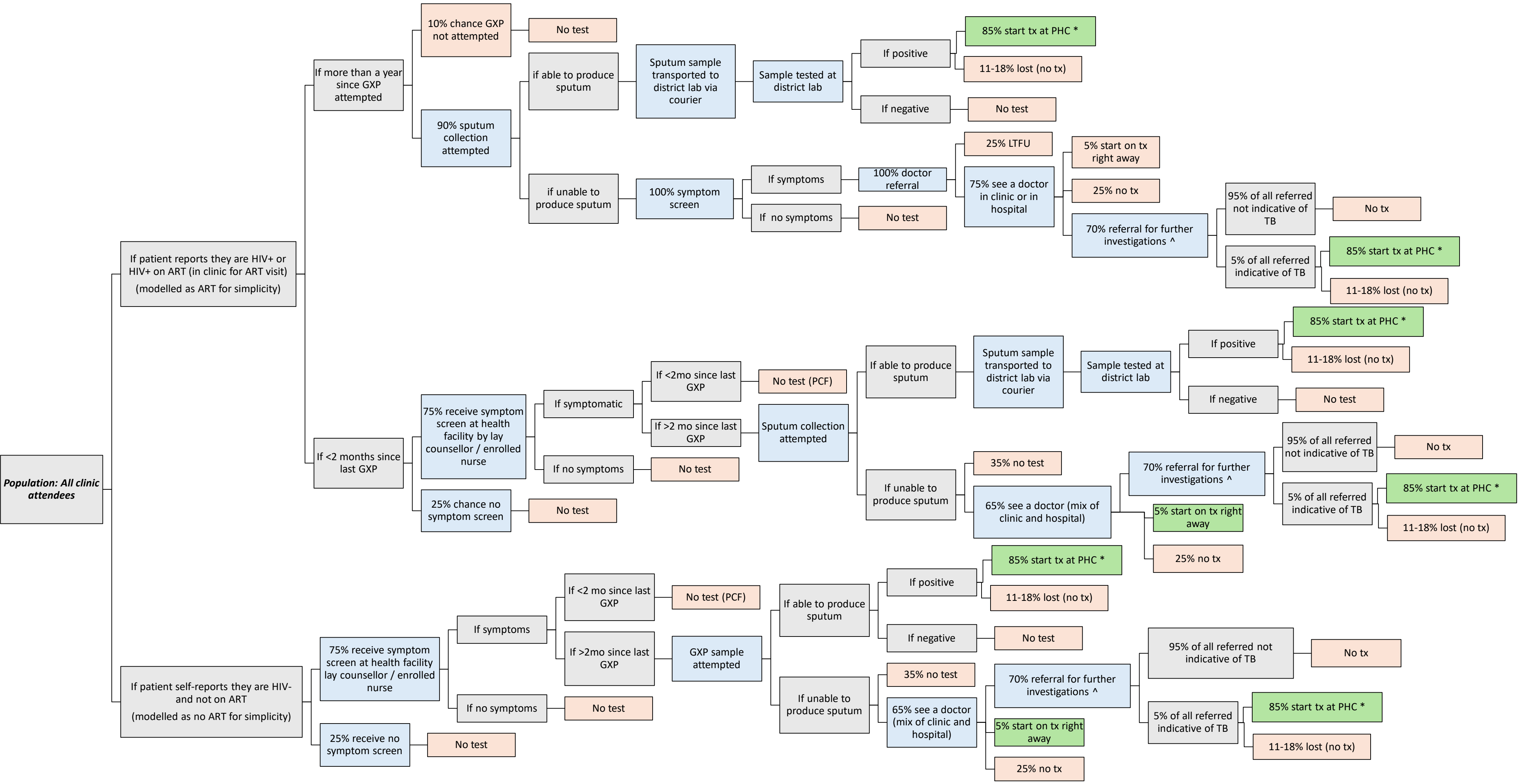

* Linkage to care activities: 50% of people testing positive called by nurse, 40% of people receiving GXP notified of result via automated text message by lab

^ Further investigations: 100% chest xray, 50% sputum attempt, 25% GeneXpert, 10% blood test FBC
