# Supplementary material for: Projected population level impact and cost-effectiveness of clinic and community-based tuberculosis screening approaches
Source: PLOS Glob Public Health. 2026 Sep 11;6(9):e0006738. doi: 10.1371/journal.pgph.0006738 (PMC13567835; doi:10.1371/journal.pgph.0006738)
Supplement: Supplementary file 3 [file pgph.0006738.s003.pdf]

Reviewer Report

Overall Assessment

This manuscript presents a comprehensive mathematical modelling and cost-effectiveness analysis comparing clinic- and community-based TB screening strategies in South Africa. The work is timely, given the current funding constraints and provides policy-relevant evidence for TB screening prioritisation. The manuscript is logically organised, scientifically rigorous, and clearly written. The modelling methodology is appropriate and appears robust, while the economic evaluation follows accepted standards. Results are presented clearly and the discussion appropriately interprets the findings within the existing literature.

Only minor revisions are recommended to improve language, consistency, and clarification of several methodological assumptions.

Synopsis of typographical errors to address:

Line	Issue	Suggested correction
19	South Africa National Department	South African National Department
56	that that	remove duplicate
76	South African	South Africa
120	Feb	February
149	Ses	See
163	depts	departments
Table 1	Intesified	Intensified

Minor Section Comments

1. Method Section Suggestions

Lines 95–106

Community screening assumes higher prevalence in targeted populations at lower coverage.

The assumption is reasonable, but could introduce optimistic estimates. Recommend adding one sentence explaining how this assumption was parameterised and whether alternative targeting assumptions were explored.

Lines 124–128

The mechanistic costing approach is innovative. However, it would help readers if one sentence briefly explained what distinguishes this approach from other costing methods.

Lines 161–165

The statement that no formal health economic analysis plan was developed is transparent. However, the authors should briefly explain whether this could introduce analytical bias or whether all analyses were prespecified before model runs.

Results Section Suggestions

Lines 179–184

The explanation for why clinic symptom screening performs best is good. However, adding one sentence explicitly linking increased Xpert coverage to reduced diagnostic delay would improve readability.

Discussion Section Suggestions

1. The discussion appropriately highlights why community radiography is cost-effective. It would be useful to include one sentence discussing practical implementation barriers such as staffing, maintenance and mobile unit availability, which are only briefly mentioned later.
2. The discussion on emerging diagnostic technologies is valuable. Potentially consider acknowledging that future reductions in diagnostic costs may alter expansion pathways, even if relative rankings remain similar.
3. The limitations are well discussed, but one additional limitation could be acknowledged: The model assumes relatively stable behavioural responses and does not consider potential changes in health-seeking behaviour following expanded screening programmes.
4. In the discussion, several sentences are relatively long (>40 words). Consider breaking them into shorter sentences, which should improve readability.

Recommendation: Minor Revision

This is a high-quality manuscript addressing an important policy question. The modelling framework and economic evaluation are rigorous, the results are well presented, and the discussion is balanced and appropriately acknowledges limitations. Only minor revisions are recommended to improve clarity, correct typographical errors, and provide additional explanation of several methodological assumptions. There are no major scientific concerns.
