# Supplementary material for: Projected population level impact and cost-effectiveness of clinic and community-based tuberculosis screening approaches
Source: PLOS Glob Public Health. 2026 Sep 11;6(9):e0006738. doi: 10.1371/journal.pgph.0006738 (PMC13567835; doi:10.1371/journal.pgph.0006738)
Supplement: Supplementary file 4 [file pgph.0006738.s005.docx]

**We thank the editor and reviewers for their comments. Please see our point-by-point responses below. Line numbers refer to the version with tracked changes.**

In addition to the reviewer's comments, please note that WBOT is explained in line 105 even though the acronym is first used in line 97.

**Thanks for flagging this – we have corrected it.**

In the online submission form, you indicated that your data will be submitted to a repository upon acceptance. We strongly recommend all authors deposit their data before acceptance, as the process can be lengthy and hold up publication timelines. Please note that, though access restrictions are acceptable now, your entire minimal dataset will need to be made freely accessible if your manuscript is accepted for publication. This policy applies to all data except where public deposition would breach compliance with the protocol approved by your research ethics board. If you are unable to adhere to our open data policy, please kindly revise your statement to explain your reasoning and we will seek the editor's input on an exemption.

**Thank you for highlighting this requirement.**

**Data from the household contact study have been made available on LSHTM Data Compass (**[**https://datacompass.lshtm.ac.uk/id/eprint/4932/**](https://datacompass.lshtm.ac.uk/id/eprint/4932/)**)**

**We have made the full dataset underlying the provider-incurred cost estimates available through LSHTM Data Compass (DOI:** [**https://doi.org/10.17037/DATA.00005321**](https://doi.org/10.17037/DATA.00005321)**).**

**The patient-incurred cost estimates used in this manuscript are derived from a separate study that is currently being prepared for submission as an independent manuscript. To avoid compromising that planned publication, we have not yet deposited the full underlying dataset in a public repository. We intend to make these data publicly available upon submission of the companion manuscript, and certainly before publication of the present manuscript if it is accepted.**

**If this approach is not compatible with the journal's data availability policy, we would be grateful if the editor could advise whether an exemption or alternative arrangement would be appropriate.**

| **Synopsis of typographical errors to address: Line** | **Issue** | **Suggested correction** |
| --- | --- | --- |
| 19 | South Africa National Department | South African National Department |
| 56 | that that | remove duplicate |
| 76 | South African | South Africa |
| 120 | Feb | February |
| 149 | Ses | See |
| 163 | depts | departments |
| Table 1 | Intesified | Intensified |

**We thank the reviewer for their careful attention to detail, and have made all the suggested corrections**

Lines 95–106 Community screening assumes higher prevalence in targeted populations at lower coverage. The assumption is reasonable, but could introduce optimistic estimates. Recommend adding one sentence explaining how this assumption was parameterised and whether alternative targeting assumptions were explored.

**Thanks for this suggestion. We have added some more detail to the methods:**

***“For all community screening scenarios, we modelled a range of annual population coverages, assuming a higher prevalence in areas targeted when coverage was lower, and parameterised using data on district-level variation in notification rates as a proxy for geographical variation in prevalence.” (lines 96 and 97)***

**We also discuss this assumption in more detail in the discussion section:**

**“*Community case finding is likely to be targeted at areas of the country where it is thought that there is a higher prevalence of TB. To model the relationship between intervention coverage and the prevalence among those screened, we used district-level variation in notification rates as a proxy—recognising that this is an imperfect measure. We may therefore be under- or over-estimating how the impact and cost-effectiveness of the community approaches scales with their coverage level. We also did not include the additional programme costs of covering larger population shares, which may require extra mobilisation, logistics, and governance resources, potentially raising ICERs at higher coverage. Given the geographic clustering of TB, simple geographic targeting would capture much of the burden, and subnational TB managers are often already aware where the highest prevalence lies.”* (lines 291 - 299)**

Lines 124–128 The mechanistic costing approach is innovative. However, it would help readers if one sentence briefly explained what distinguishes this approach from other costing methods.

**Thanks for this suggestion. We’ve added some further detail about this in the discussion:**

***“Another strength is our use of a mechanistic cost function allowing for distinction between fixed and variable costs. Costing analyses alongside transmission modelling often adopt a simplistic “price times quantity” approach, assuming constant marginal costs regardless of scale. This runs counter to economic theory and ignoring ignores important economies of scale and scope. By scaling fixed costs at a different rate than variable cost, our approach allows for consideration of both economies of scale and scope and therefore provides a more robust estimate of national-level costs and cost-effectiveness at scale.”* (lines 275-281)**

Lines 161–165 The statement that no formal health economic analysis plan was developed is transparent. However, the authors should briefly explain whether this could introduce analytical bias or whether all analyses were prespecified before model runs.

**This is a good point. All our analyses were prespecified and based on established best-practice methodologies. We have added a couple lines to this effect:**

**“*As this is an exploratory modelling exercise, no explicit prior health economics plan was developed. However, all analytic choices (including the application of a mechanistic cost model, cost-effectiveness thresholds, and estimation of expansion paths) were pre-specified and guided by established best practice methodology.*” (lines 161-165)**

Lines 179–184 The explanation for why clinic symptom screening performs best is good. However, adding one sentence explicitly linking increased Xpert coverage to reduced diagnostic delay would improve readability.

**We have added extra words, explicitly linking increased Xpert testing to reduced diagnostic delays:**

**“*This reflects the higher population-level coverage of Xpert testing compared with other approaches (median coverage of 9.7% in 2026, compared to 3.6% - 8.1%, Figure S5), and an increased probability of Xpert testing and diagnosis per clinic visit for people with TB seeking care for TB symptoms, reducing diagnostic delays.*” (lines 184-187)**

1. The discussion appropriately highlights why community radiography is cost-effective. It would be useful to include one sentence discussing practical implementation barriers such as staffing, maintenance and mobile unit availability, which are only briefly mentioned later.

**We agree this is an important point and have expanded on this a bit in the discussion:**

**“*Similarly, there could be limitations in implementation of mobile radiographic screening due to issues with staffing, maintenance, and mobile unit availability.*” (lines 285-287).**

2. The discussion on emerging diagnostic technologies is valuable. Potentially consider acknowledging that future reductions in diagnostic costs may alter expansion pathways, even if relative rankings remain similar.

**Thanks for the suggestion – we have added a sentence to this effect:**

**“*Future changes in technology prices could similarly change the order of expansion paths, even if the overall strategy rankings remain similar*” (lines 264 -265)**

3. The limitations are well discussed, but one additional limitation could be acknowledged: The model assumes relatively stable behavioural responses and does not consider potential changes in health-seeking behaviour following expanded screening programmes.

**Thanks for this suggestion. We have added the following sentence to the discussion:**

***“We may also be underestimating the impact of the clinic-based approaches, if expanded screening programmes in clinics lead to increases in health-seeking behaviour.” (lines 299-301)***

4. In the discussion, several sentences are relatively long (>40 words). Consider breaking them into shorter sentences, which should improve readability.

**Thanks for highlighting this. We have split a number of sentences in the discussion in two.**
